# A Review on the Structural Evolution and Clinical Evidence of Boronic Acid β-Lactamase Inhibitors

**DOI:** 10.3390/molecules31203647

**Published:** 2026-10-13

**Authors:** Wenbo Zou, Li Zhu

**Affiliations:** National Institutes for Drug Control, Beijing 102629, China

**Keywords:** boronic acid, carbapenemase, metallo-β-lactamase, taniborbactam, vaborbactam, xeruborbactam

## Abstract

The spread of carbapenem-resistant Gram-negative bacilli has made β-lactam/β-lactamase inhibitor (BLI) combinations central to therapy. Boronic acids are the only BLI class whose chemistry can in principle reach all four Ambler classes, boron forms a tetrahedral adduct with the catalytic serine and can also coordinate the zinc ions of metallo-β-lactamases. PubMed/MEDLINE and Web of Science were searched to 30 September 2026, together with ClinicalTrials.gov and the Protein Data Bank, for structural, biochemical, surveillance, and clinical data on vaborbactam, taniborbactam, and xeruborbactam. The three generations are compared across enzyme classes, the diazabicyclooctane durlobactam is used as a mechanistic comparator, and the pivotal phase 3 trials are appraised against the requirements of enzyme-guided therapy. The scaffold has advanced from KPC-selective monocyclic to bicyclic compounds that inhibit serine and subclass B1 metallo-β-lactamases in vitro at nanomolar concentrations, but this breadth remains largely unrealized clinically. Reported potencies differ by one to two orders of magnitude between laboratories, coverage of IMP, SPM, and subclass B3 enzymes is incomplete, and no registrational trial has been designed around carbapenemase genotype. Partner β-lactam selection and trial design now constrain the field more than target engagement does.

## 1. Introduction

Carbapenems are still first-line against infections caused by extended-spectrum β-lactamase (ESBL)-producing Enterobacterales, but the global dissemination of carbapenemases is eroding their clinical value. Bush and Bradford mapped the geographic distribution of these enzymes: KPC (*Klebsiella pneumoniae* carbapenemase) enzymes predominate in the Americas, the Mediterranean basin, and China; NDM (New Delhi metallo-β-lactamase) enzymes in the Indian subcontinent, Eastern Europe, and the Middle East. OXA (oxacillinase)-48-like enzymes predominate in Turkey, Spain, and the Mediterranean region [1]. Before the introduction of the newer BLI combinations, mortality from CR-GNB infection was high: with conventional regimens, the 30-day case-fatality rate for bloodstream infections caused by KPC-producing *K. pneumoniae* reached 55.8%, and the risk of death was greatest for MBL-producing strains [2]. The World Health Organization in its 2024 Bacterial Priority Pathogens List (BPPL) has classified carbapenem-resistant *Acinetobacter baumannii* (CRAB), carbapenem-resistant *Pseudomonas aeruginosa* (CRPA), and carbapenem-resistant Enterobacterales (CRE) as priority pathogens, marking the arrival of the post-carbapenem era.

The standard response to carbapenemases is to combine a β-lactam with an inhibitor of the offending enzyme. The first-generation inhibitors, clavulanic acid, sulbactam, and tazobactam, retain activity against many Ambler class A enzymes but are essentially inactive against carbapenemases. The inhibitors developed over the past decade fall into three chemical classes: diazabicyclooctanes (DBOs; e.g., avibactam and relebactam), cyclic boronates (e.g., vaborbactam and taniborbactam), and newer penicillanic acid sulfones related to sulbactam and tazobactam [3,4]. Of these, only the cyclic boronates inhibit both serine β-lactamases (SBLs) and MBLs [5,6,7].

DBOs inhibit SBLs by carbamoylating the active-site serine, forming a reversible covalent adduct that arrests the catalytic cycle [8,9]. Because this mechanism requires a nucleophilic serine, and because the MBL active site is organized around Zn^2+^ ions and contains no such residue, DBOs are inactive against MBLs. Boronic acids act differently: the boron atom forms a covalent tetrahedral adduct with the catalytic serine that mimics the transition state leading to the acyl-enzyme intermediate, and it can also coordinate the Zn^2+^ ions of the MBL active site [10]. In principle this lets one scaffold act across all four Ambler classes.

This review follows three generations of cyclic boronates, vaborbactam, taniborbactam, and xeruborbactam, and asks how structural change has reshaped the inhibitory spectrum and clinical prospects of the class. Each successive scaffold modification has widened the range of enzymes inhibited and lowered MICs against resistant organisms. Clinically, however, taniborbactam still shows a substantial activity gap against IMP (imipenemase)-type MBLs [11], and the partner β-lactam for xeruborbactam has yet to be optimized. The pan-spectrum advantage of the boronic acids therefore remains largely theoretical, and translating it into clinical practice has proved harder than the chemistry.

Several recent reviews have surveyed β-lactamase inhibitors broadly or have covered the entire chemical landscape of boronic acid inhibitors from early phenylboronates to the most recent clinical candidates [12,13]. The present review differs in three respects: (i) it organizes the field as a deliberate three-generation structural evolution, monocyclic vaborbactam, bicyclic taniborbactam, and multidentate xeruborbactam, treating each scaffold change as a testable hypothesis about how enzyme-class coverage is expanded; (ii) it pairs each structural generation with the pivotal clinical trial that did, or did not, test it against the enzyme class it was designed to cover; and (iii) it uses durlobactam as a mechanistic counterpoint to delimit what a serine-only inhibitor can achieve clinically.

## 2. Literature Search and Study Selection

This narrative review was conducted and reported with reference to the PRISMA 2020 statement [14], adapted to the scope of a narrative synthesis. PubMed/MEDLINE (via the NCBI platform) and the Web of Science Core Collection (Clarivate) were searched from database inception to 30 September 2026, using combinations of the terms “boronic acid”, “cyclic boronate”, “vaborbactam”, “RPX7009”, “taniborbactam”, “VNRX-5133”, “xeruborbactam”, “QPX7728”, “durlobactam”, “ETX2514”, “ledaborbactam”, and “VNRX-7145”, each combined with “β-lactamase inhibitor”, “carbapenemase”, and “metallo-β-lactamase”. The complete database-specific search strings, including Boolean operators, are provided in Supplementary Table S1. ClinicalTrials.gov was searched for registered trials of the three boronate inhibitors, and the Protein Data Bank was queried for co-crystal structures of boronate–β-lactamase complexes. The reference lists of all included articles were screened manually for additional eligible studies.

Records were exported and deduplicated in a spreadsheet environment, first by normalized title matching and then verified manually. Titles and abstracts were screened against prespecified eligibility criteria: reports of structural, biochemical, microbiological, pharmacological, or clinical evaluation of boronic acid β-lactamase inhibitors (or the diazabicyclooctane comparator durlobactam) were eligible; conference abstracts without primary data, corrections, patents, and reports outside this scope were excluded. Full texts of potentially eligible reports were assessed, with exclusions recorded and categorized. In addition, eligible reports were cited only when they contributed data or analysis beyond that already covered by the selected sources; otherwise they were excluded as superseded or redundant. Because inhibition constants reported in this field vary between laboratories owing to differences in substrate, enzyme preparation, and pre-incubation conditions, biochemical values are presented as reported rather than pooled. Where several reports described the same dataset, the most complete peer-reviewed publication was cited.

The database searches identified 2284 records (PubMed/MEDLINE *n* = 1000; Web of Science *n* = 1284). After removal of 807 duplicate records and 21 records without retrievable bibliographic data, 1456 records were screened by title and abstract, of which 202 were excluded. Of the 1254 reports assessed for eligibility, 1212 were excluded with reasons (Supplementary Table S2) and 42 studies were included. A further 14 records were identified through citation searching of reference lists, and 18 registered clinical trials were identified in ClinicalTrials.gov, yielding a final evidence base of 56 publications and 18 trial registrations. The selection process is documented in the PRISMA 2020 flow diagram (Figure 1).

## 3. Molecular Basis of β-Lactamase Inhibition by Boronic Acids

### 3.1. Transition-State Mimicry and Reversible Covalent Binding at the Serine Active Site

The key intermediate in the hydrolysis of β-lactams by β-lactamases is a high-energy tetrahedral transition state, and boronic acid inhibitors were designed to mimic it. The boron atom is trigonal planar and sp^2^-hybridized, and its vacant p orbital is readily attacked by the hydroxyl oxygen of the active-site serine, converting boron to sp^3^ and generating a thermodynamically stable tetrahedral boronate adduct. The geometry of this adduct closely reproduces that of the hydrolytic transition state, and the partial negative charge on boron mimics the electron density of the oxyanion formed in that state. This dual match, in geometry and in charge distribution, accounts for the exceptionally tight interaction between boronic acid inhibitors and β-lactamases.

The same principle is visible within a single molecule. In the complex of taniborbactam with the class A enzyme CTX-M-15 (PDB 6SP6), the catalytic Ser70 is covalently bound to boron, one boron hydroxyl occupies the oxyanion hole, and the inhibitor amide is held by hydrogen bonds to Asn104, Asn132, and the Ser237 backbone carbonyl, while the carboxylate hydrogen-bonds to Thr235 of the K234TG motif. In the complex of the same compound with VIM-2 (PDB 6SP7), the tetrahedral boron instead coordinates Zn1 [15]. One scaffold therefore reproduces the tetrahedral intermediate of serine-based and of zinc-based hydrolysis with essentially the same core geometry. What differs between the two settings is the behavior of the side chain: the acylamino substituent makes no contact with the enzyme in the class A structure, whereas in VIM-2 the proximal amine is recruited into the active site [15]. Side-chain optimization thus carries different consequences for the serine enzymes than for the MBLs, which is one structural reason why a scaffold that reaches both classes is difficult to optimize for both at once. The versatility of boron in this role is not limited to a single geometry. In the co-crystal structure of taniborbactam with NDM-1 (PDB 6RMF), the electron density is best explained by a tricyclic species generated by cyclization of the side-chain amide oxygen onto boron, refined at full occupancy in one chain and modeled as a 60:40 mixture with the bicyclic form in the other. Inhibitor binding also widens the Zn1–Zn2 separation from about 3.6 Å to 4.3 Å [11]. The capacity of a boron center to interconvert between sp^2^, sp^3^, bicyclic, and tricyclic states is therefore part of what allows one scaffold to engage active sites as different as those of the serine enzymes and the MBLs [11,15].

The binding chemistry of the boronates differs fundamentally from that of both the first-generation inhibitors and the DBOs. Clavulanic acid and sulbactam acylate the catalytic serine irreversibly to form an ester, but once acylation is complete the inhibitor itself undergoes ring opening and degradation, so inhibitory activity decays over time. DBOs, exemplified by avibactam, bind covalently through reversible carbamoylation of the serine; although the reaction is reversible, the intact strained ring of the inhibitor can still be recognized by β-lactamases as a potential substrate. The mechanism depends strictly on a nucleophilic serine, whereas the MBL catalytic center is organized around Zn^2+^ and offers no serine for carbamoylation, which is why DBOs are inactive against MBLs [8,9]. Boronic acids, by contrast, require no β-lactam ring and undergo no ring-opening degradation; their boronate adduct is reversible but is not hydrolyzed. Because the boronate adduct is intrinsically stable, side-chain and ring-system modifications can be directed at spectrum and permeability without having to re-engineer inhibitor stability at the same time.

In their review of design strategies for boronic acid inhibitors, Plescia and Moitessier identified covalent addition of boron to the catalytic serine as one of the most successful applications of transition-state mimicry in β-lactamase inhibitor design [16]. The co-crystal structure of vaborbactam with KPC-2 (PDB 6V7I) resolves this mechanism at atomic detail: the boronic acid group forms a 1.4-Å B–O covalent bond with the hydroxyl oxygen of Ser70, and its carboxamide group makes hydrogen bonds with Ser130, Thr235, and Thr237 that lock the molecule into the active site [17]. The thiopheneacetyl side chain occupies a hydrophobic pocket formed by Trp105, Cys69, and Pro107 and is further stabilized by π–π stacking and hydrophobic contacts (Figure 2A). Time-resolved crystallography by Prester et al. showed that binding of a boronic acid to the catalytic serine of CTX-M-14 proceeds through a rapid 1,2-diol esterification step: in electron density maps collected 150 ms after mixing, the glycerol boronate diester was already clearly resolved at 51% occupancy, with a geometry corresponding unambiguously to the serine–boronate adduct [18].

### 3.2. From Monocyclic to Bicyclic Scaffolds

Vaborbactam is built on a six-membered monocyclic boronate whose conformation is highly complementary to class A active sites. It potently inhibits class A enzymes, including ESBLs (TEM-10, Ki 0.110 µM; SHV-12, 0.029 µM; CTX-M-15, 0.044 µM), KPC-2 (Ki 0.069 µM), and SME-2, as well as class C cephalosporinases (P99, Ki 0.053 µM; CMY-2, 0.099 µM) [19]. Its clinical value, however, is concentrated in KPC-producing organisms, because it is essentially inactive against class B metallo-β-lactamases (IC50 >160 µM against NDM-1, VIM-1, and IMP-1) and only weakly active against class D enzymes (OXA-48, IC50 6.9 µM; OXA-23, 120 µM) [10].

Early crystallographic work by Brem et al. had already shown that cyclic boronates can inhibit both SBLs and MBLs, but that the spectrum obtained depends on the specific configuration of the boronate ring system [20].

Taniborbactam is built on a bicyclic boronate scaffold in which the conformational constraint imposed by the bridged ring reorients the boronic acid group, allowing it to adapt to a wider range of binding-pocket geometries. The added rigidity reduces the entropic penalty of binding and markedly increases affinity for the class A, C, and D serine enzymes. At the same time, the additional oxygen atom in the bridged ring confers an initial capacity to coordinate Zn^2+^, making taniborbactam the first boronic acid inhibitor with activity against MBLs. Co-crystal data from Krajnc et al. show that one boronate oxygen of taniborbactam bridges Zn1 and Zn2 in the NDM-1 active site, at 1.9 Å and 3.0 Å, respectively (Figure 2B). This bridging mode underlies potent inhibition of subclass B1 MBLs, with IC_50_ values of 0.01, 0.0079, and 0.0005 µM against NDM-1, VIM-1, and VIM-2, respectively [11]. VIM (Verona integron-encoded metallo-β-lactamase) enzymes constitute another major subgroup of the class B MBLs. Molecular dynamics simulations by Lence et al. further indicated that the conformational rigidity of the bicyclic boronate stabilizes binding within MBL active sites [21]. The move from a monocyclic to a bicyclic scaffold, however, raises the molecular weight from roughly 300 to roughly 500 Da and increases the polar surface area, which may compromise penetration of the Gram-negative outer membrane (Figure 2D).

One caveat governs everything that follows: the spectrum of a β-lactam/β-lactamase inhibitor combination is the intersection of the inhibitor’s enzyme coverage with the partner β-lactam’s intrinsic activity, outer-membrane permeability, PBP affinity, and susceptibility to efflux. “Pan-class inhibition” therefore describes the inhibitor alone, never the finished product. Xeruborbactam illustrates the point: its ultrabroad enzyme profile is realized in vitro only with partner β-lactams that themselves reach the target, and pairing a pan-class inhibitor with a weak partner risks selecting for resistance against the inhibitor’s own PBP-binding activity [22].

## 4. Structural Comparison of the Boronic Acid Inhibitors

### 4.1. Vaborbactam: Precise Complementarity with the KPC Active Site

Vaborbactam, approved in 2017, is the only clinically approved boronic acid β-lactamase inhibitor; the clinical evidence below derives from the phase 3 TANGO program (Section 6). The potency of vaborbactam against KPC-2 reflects the close complementarity between the inhibitor and the topology of the KPC-2 active site. The co-crystal structure (PDB 6V7I) shows that the 2-thiopheneacetyl side chain stacks against Trp105 and the acetamido group hydrogen-bonds to Ser70 [17]. Biochemically, vaborbactam inhibits KPC-2 with a Ki of 0.069 µM, compared with 0.029 µM for SHV-12, 0.044 µM for CTX-M-15, 0.110 µM for TEM-10, and 0.053 µM for AmpC [19]. The compound’s superiority against KPC-2 arises not from tighter static binding but from faster association and markedly slower dissociation, conferring an approximately 15-fold higher initial binding affinity than that for CTX-M-15 [17]. Thus, even within class A, small topological differences in the active site measurably change the kinetics of inhibition.

This molecular complementarity is preserved in vivo. Castanheira et al. found that, at a fixed vaborbactam concentration of 8 µg/mL, meropenem-vaborbactam had an MIC_90_ of 1 µg/mL against KPC-producing Enterobacterales [23]. Global surveillance from the same group showed that meropenem-vaborbactam inhibited all 135 KPC-producing isolates at MICs ≤8 µg/mL, with 98.5% (133/135) inhibited at ≤2 µg/mL (MIC_50_/_90_, 0.12/0.5 µg/mL) [24]. The corollary of such high specificity is a narrow spectrum: although vaborbactam retains activity against other class A and class C enzymes, it is essentially inactive against class B and class D carbapenemases (IC_50_ >160 µM against NDM-1, VIM-1, and IMP-1; 6.9 µM against OXA-48 and 120 µM against OXA-23) [10]. In the SMART global surveillance program (2016–2021), Karlowsky et al. found marked geographic clustering of MBL carriage in *P. aeruginosa*, with rates above 5% in Eastern Europe (9.5%), Latin America (7.9%), and the Middle East/Africa, and reported that 76% of isolates resistant to both newer combinations carried an MBL [25], which is part of why pan-class inhibitors are being pursued.

The same structural fit that gives vaborbactam its KPC potency also sets its limits. The monocyclic boronate leaves little room for side-chain elaboration: quinazoline or other aromatic substituents would disrupt the fit to the KPC active site, whereas simple alkyl extensions cannot supply the additional contacts needed for OXA enzymes or MBLs. Precise complementarity also implies sensitivity to perturbation of the active site. In vitro selection experiments by Sun et al. yielded meropenem-vaborbactam-resistant mutants with 4- to 16-fold MIC increases, driven mainly by upregulation of *bla*KPC-2 and loss of the OmpK36 porin [26]. Structural studies of KPC-2 Ω-loop variants indicate that vaborbactam is less affected by substitutions at D179 than avibactam is, and that its binding mode in the D179N variant is essentially identical to that in the wild-type enzyme [27], suggesting that the interaction tolerates certain structural perturbations. Molecular complementarity must also be sustained in vivo if it is to translate into efficacy. In phase 1 studies, vaborbactam had a terminal half-life of about 2 h with more than 80% of the dose excreted unchanged in the urine, supporting an 8-hourly dosing regimen [28], and Rubino et al. confirmed the pharmacokinetic compatibility of meropenem and vaborbactam co-administered to healthy adults [29].

### 4.2. Taniborbactam: A Breakthrough in MBL Inhibition

Taniborbactam has completed phase 3 evaluation in complicated urinary tract infection (CERTAIN-1; NCT03840148); regulatory review is ongoing at the time of writing. Taniborbactam is where the boronic acids crossed from KPC-targeted inhibition into MBL coverage. Compared with the monocyclic boronate of vaborbactam, its bicyclic scaffold, rigidified by the bridged ring, binds the active sites of the class A, C, and D serine enzymes more tightly, while the bridging oxygen provides weak coordination to the Zn^2+^ ions of VIM enzymes. The co-crystal structures of taniborbactam with NDM-1 and with VIM-2 identify the contacts that underlie this activity. In NDM-1, the aryl carboxylate of the inhibitor engages Zn2 together with Lys224 [11]; in VIM-2 (PDB 6SP7), Zn2 is coordinated by the boronate carboxylate and phenolic oxygens, and the proximal amine of the side chain contacts Glu149 [15]. Building on the first of these observations, Ono et al. showed that conformational changes in the K224 side chain govern the stability of Zn^2+^ coordination by taniborbactam [30]. The consequences of this expanded target range, and its limits, are evident in susceptibility surveillance. In a Spanish national survey, Hernández-García et al. reported that cefepime-taniborbactam inhibited 97.6% of carbapenemase-producing Enterobacterales and 67.1% of carbapenem-resistant pseudomonads at ≤8/4 mg/L; all OXA-48-producing isolates were susceptible, whereas VIM-20-producing ST175 *P. aeruginosa* isolates had MICs of 16/4 to >32/4 mg/L [31]. Taniborbactam is considerably less active against IMP (imipenemase) enzymes than against NDM and VIM. Structural work indicates that its positively charged side-chain amine is stabilized by a salt bridge to a negatively charged glutamate in the NDM/VIM active site (E152 in NDM-1, E149 in VIM-2), an electrostatic contact that cannot form efficiently in IMP-type enzymes [10,15,32,33]. Taniborbactam thus achieved a breakthrough in MBL coverage while leaving a structurally defined gap in its spectrum. As a concrete illustration of inter-laboratory variability, the IC50 of taniborbactam against IMP-1 has been reported as 2.51 µM by Krajnc et al. [11] and as 39.8 µM by Liu et al. [15], a 16-fold difference between laboratories using different substrates and assay formats; its NDM-1 IC50 similarly ranges from 0.01 to 0.19 µM across studies. We therefore treat absolute IC50 values as comparable only within a single study and regard the IMP gap as qualitative: in every individual dataset, taniborbactam is at least two orders of magnitude less potent against IMP-1 than against NDM-1 or VIM-2.

### 4.3. Xeruborbactam: Optimizing IMP Coverage

Xeruborbactam (QPX7728) was designed specifically to close the IMP gap [32]. All activity data in this section derive from in vitro and preclinical studies; no phase 2 or 3 efficacy data exist as of 30 September 2026. It adds a further Zn^2+^-coordinating group to the bicyclic boronate scaffold of taniborbactam and optimizes both the coordination distance and the identity of the coordinating atoms. Biochemically, xeruborbactam inhibits IMP-1 with an IC_50_ of 0.61 µM and a Ki of 0.24 µM [10], against reported IC_50_ values for taniborbactam that range from 2.51 to 39.8 µM [11,15]; on either estimate xeruborbactam is the more potent IMP inhibitor. The advantage is not confined to IMP enzymes: NDM-9, in which the E152K substitution abolishes the negatively charged pocket and renders the enzyme insensitive to taniborbactam (IC_50_ > 60 µM), is inhibited by xeruborbactam almost as well as NDM-1 (IC_50_, ~1.2 versus 0.77 µM) [25], indicating that its binding does not depend on the electrostatic contacts that taniborbactam requires. Dhankhar et al. solved the co-crystal structure of xeruborbactam with the class A carbapenemase SME-1 (PDB 9VA4) and reported an apparent Ki of 4 nM, providing atomic-level evidence for high-affinity boronate adduct formation with the catalytic serine [34]. A systematic review by Safavi et al. confirmed that NDM variants remain endemic in the Indian subcontinent and continue to spread globally in *K. pneumoniae* [35]; so activity against NDM variants matters most in precisely the regions where these agents are most needed.

Microbiological data reflect the same generational gap. Combined with meropenem at a fixed 8 µg/mL, xeruborbactam gave an MIC_90_ of 1 µg/mL against MBL-positive CRE, whereas cefepime-taniborbactam had an MIC_90_ of 16 µg/mL against the same isolate set [33]. Sun et al. further showed that xeruborbactam has intrinsic antibacterial activity, with MICs of 16 to 32 µg/mL against *Escherichia coli* and *K. pneumoniae*, arising from binding to PBP1a, PBP1b, PBP2, and PBP3 [22]. Earlier work by Lomovskaya et al. showed that xeruborbactam restores the activity of several oral β-lactams against resistant Enterobacterales [36]. On this basis, Paterson listed xeruborbactam among the most promising Gram-negative agents in the current clinical pipeline [37], although that assessment still rests largely on in vitro and early-phase clinical data. The progressive expansion of the inhibitory spectrum from KPC-selective to near-pan-class coverage across the three generations is summarized in Table 1.

### 4.4. Resistance Mechanisms Beyond the Target

Selection experiments and clinical-genomic studies with meropenem–vaborbactam (MVB) show that resistance rarely involves the inhibitor-binding site itself. In vitro selection of KPC-producing *K. pneumoniae* yielded mutants with 4- to 16-fold MIC increases driven by blaKPC-2 upregulation and porin loss rather than active-site substitutions [26]. In clinical isolates, reduced susceptibility maps predominantly to permeability and gene-dose mechanisms: median MVB MICs were eightfold higher against KPC-producing *K. pneumoniae* with mutant ompK36 than against wild-type isolates (0.25 vs. 0.03 µg/mL; *p* < 0.0001), with IS5 promoter insertions and 134–135 GD insertions in the L3 loop among the recurrent genotypes [40]. Whole-genome analysis of a pre-availability cohort (CRACKLE-1, isolates collected 2012–2018) found that 16% (19/116) of KPC-*K. pneumoniae* already exhibited non-susceptible MVB MICs, encoding truncated OmpK36, the L3-loop GD insertion, or elevated blaKPC copy number (up to ~42 per cell); one isolate with an MIC > 16/8 µg/mL carried a unique EnvZ A225V mutation in the EnvZ–OmpR two-component system [41]. These findings are consistent with the preferential use of OmpK36 by vaborbactam, unlike meropenem, which traverses OmpK35 and OmpK36 with roughly equivalent efficiency, and imply that prior carbapenem exposure can narrow the utility of carbapenem–boronate combinations [41].

For xeruborbactam, the relevant background is both intrinsic and extrinsic. Xeruborbactam has intrinsic antibacterial activity (MICs 16–32 µg/mL against *E. coli* and *K. pneumoniae*) through binding to PBP1a, PBP1b, PBP2, and PBP3 [22], a double-edged property that contributes to efficacy but means partner-drug failure can select for inhibitor resistance. Its potency is attenuated by MexAB-OprM in *P. aeruginosa* and by AdeIJK in A. baumannii [22], and in *K. pneumoniae* the OmpK36 GD insertion and porin loss are associated with MICs ≥ 32 µg/mL.

Target-site mutations are the exception. Cyclic boronates are relatively tolerant of the KPC-2 Ω-loop substitutions (D179N, D179Y) that strongly impair avibactam: the vaborbactam binding mode is essentially unchanged in the D179N variant [27], and cefepime–taniborbactam retains activity against KPC variants that confer ceftazidime–avibactam resistance [13]. Cross-resistance between the DBO and boronate classes is therefore limited. In contrast, mechanisms acting on drug entry, efflux, and enzyme dose (porin loss, efflux upregulation, and blaKPC copy-number amplification) affect any β-lactam/BLI combination and cannot be overcome by broadening the inhibitor’s enzyme spectrum.

## 5. Boronic Acid Inhibitors Versus DBO Inhibitors

We selected durlobactam, rather than avibactam or relebactam, as the mechanistic comparator for three reasons: it is the only DBO whose pivotal phase 3 trial (ATTACK) targeted a class D enzyme-driven pathogen (CRAB) and achieved regulatory approval, providing the cleanest clinical test of what serine-only inhibition can achieve. Its inhibition kinetics are unusually well characterized (kinact/Ki and koff across classes A, C, and D) [42], permitting direct comparison with boronate kinetics; and the sulbactam–durlobactam combination illustrates a complementary clinical niche, intrinsic partner-drug antibacterial activity, that no boronate combination currently replicates. The divergence in spectrum between the DBOs and the boronic acids follows directly from where each inhibitor attacks. Both classes exploit the serine enzymes’ reliance on a nucleophilic serine: DBOs such as avibactam and relebactam carbamoylate that serine, and boronic acids form a tetrahedral adduct with it (Section 3.1). Against the MBLs, the two classes part company. The MBL active site has no catalytic serine, hydrolysis is carried out by a zinc-bound hydroxide, so a DBO has nothing to carbamoylate and is inert, which is why avibactam inhibits KPC-2, AmpC, and OXA-48 yet leaves NDM-1 and VIM-1 untouched. The boronate escapes this trap because its boron does not need a serine at all: the same electron-deficient boron that binds serine can instead accept the zinc-bound hydroxide and bridge the two active-site zinc ions. One inhibitor chemistry thus reaches both catalytic mechanisms; the other, by design, reaches only one (Figure 2C). The key differences in binding chemistry, dependence on β-lactam ring, spectrum of enzyme inhibition, and resistance mutation profiles between the DBOs and boronic acids are summarized in Table 2.

Boronic acids inhibit SBLs through transition-state mimicry and MBLs through Zn^2+^ coordination, and can therefore, in principle, span all four Ambler classes. How far that potential is realized varies considerably among individual molecules: vaborbactam is limited to classes A and C, with no activity against class B or D. Taniborbactam covers the class A, C, and D serine enzymes and has clinically relevant activity against NDM and VIM but insufficient activity against IMP [31]; and xeruborbactam closes the IMP gap yet is less active than taniborbactam against SPM (São Paulo metallo-β-lactamase)-1 [32]. Pan-class coverage is thus a chemical potential of the boronate scaffold rather than a property of every member of the class.

For this reason, the DBOs and the boronic acids are complementary rather than interchangeable, and that complementarity is the central logic of current treatment selection. In KPC-endemic regions, avibactam and vaborbactam compete directly, and the KPC selectivity of vaborbactam, together with its comparatively low rate of resistance-conferring mutation, may offer an advantage. For OXA-mediated CRAB infection, durlobactam is at present the only targeted option. For MBL-producing CRE, the boronates (taniborbactam and xeruborbactam) are the only BLIs with direct inhibitory activity. Where IMP-type enzymes are prevalent, both classes are constrained by insufficient in vitro activity [31,32].

## 6. Clinical Evaluation of the Approved Fixed-Dose Combinations

Four phase 3 trials define the current clinical evidence base for the combinations discussed in this review; their designs, principal results, and main limitations are summarized in Table 3.

### 6.1. Meropenem-Vaborbactam and the TANGO Trials

The registration program for meropenem-vaborbactam comprised two pivotal trials of unequal evidentiary weight. TANGO I enrolled 550 patients with complicated urinary tract infection (cUTI) and compared meropenem-vaborbactam with piperacillin-tazobactam; overall success on the FDA composite endpoint was 98.4% versus 94.0% (difference, 4.5%; *p* < 0.001), meeting the non-inferiority endpoint [43]. In the microbiological intention-to-treat (ITT) population of that trial, however, only about 12% of baseline pathogens were resistant to piperacillin-tazobactam, and the population consisted largely of ESBL rather than KPC infections. The core property of vaborbactam, KPC inhibition, was therefore not fully tested in TANGO I.

That test came from TANGO II, which compared meropenem-vaborbactam monotherapy with best available therapy (BAT) in 77 patients with CRE infections. In the mCRE-MITT population, clinical cure was achieved in 65.6% versus 33.3% (difference, 32.3%; 95% CI, 3.3% to 61.3%), and 28-day all-cause mortality was 15.6% versus 33.3% [44]. The trial was small and dominated by KPC-producing isolates, so its conclusions generalize well to KPC-CRE but provide very limited evidence for OXA-48- or MBL-producing CRE. Global surveillance data likewise show that vaborbactam does not enhance the activity of meropenem against CRE producing OXA-48-like enzymes, against which meropenem-vaborbactam performs much as meropenem alone (MIC_50_/_90_, 16/≥32 µg/mL) and far less well than against KPC-CRE [23]. An in vitro hollow-fiber model reported by Sabet et al. indicated that even against KPC-CRE the bactericidal activity of meropenem-vaborbactam depends on adequate optimization of PK/PD parameters [49].

### 6.2. Cefepime-Taniborbactam and the CERTAIN-1 Trial

CERTAIN-1 randomized 661 patients with cUTI in a 2:1 ratio to cefepime-taniborbactam (2 g/0.5 g every 8 h) or meropenem (1 g every 8 h). In the microbiological ITT population, the composite endpoint at follow-up was met by 70.6% (207/293) versus 58.0% (83/143), a difference of 12.6% (95% CI, 3.1% to 22.2%), establishing superiority in addition to non-inferiority [45]. In outcomes stratified by baseline resistance phenotype and genotype, Moeck et al. found significantly higher rates of microbiological eradication with cefepime-taniborbactam than with meropenem against ESBL- and AmpC-producing Enterobacterales [46]. In prespecified analyses of the extended microITT population, cefepime–taniborbactam achieved composite success in 7 of 8 patients (87.5%) with carbapenem-resistant Enterobacterales and in 8 of 9 (88.9%) with Enterobacterales carrying a carbapenemase gene (five OXA-48-group, two KPC-3, two NDM-1) [46]. Among 16 patients with *P. aeruginosa*, composite and clinical success rates were 50.0% and 81.3%, respectively, including the patient infected with a VIM-2-producing strain. Taniborbactam lowered the cefepime MIC90 by ≥64-fold against carbapenemase-gene-carrying isolates and inhibited 92.3% of them at ≤16 µg/mL [46].

The choice of meropenem as the comparator, however, imposes a key limitation. Most cUTI pathogens are meropenem-susceptible, and the observed superiority derives mainly from incomplete coverage of ESBL-producing strains in the meropenem arm rather than from exceptional anti-CRE activity of taniborbactam. Only 10 patients in the extended microbiological ITT population carried meropenem-resistant pathogens, all in the cefepime-taniborbactam arm (8 of whom met the composite endpoint), too few to support firm conclusions. In addition, taniborbactam is weakly active against IMP-type MBLs. Two independent studies place its IC_50_ against IMP-1 roughly two orders of magnitude above that against NDM-1 (2.51 versus 0.01 µM, and 39.8 versus 0.19 µM) [11,15]. The absolute values differ between laboratories, but the size of the gap does not, and it may limit the value of the combination where these enzymes are common. Matsumura et al. noted that IMP-producing Enterobacterales are concentrated in Asia, with particularly high prevalence in Japan and Taiwan, China [50], and Akeda likewise emphasized the distinctive prevalence of IMP-type MBLs in Japan and Southeast Asia [51].

### 6.3. The Clinical Gap in CRAB Infection and the Lesson from the DBOs

Clinical data for taniborbactam and xeruborbactam are concentrated on cUTI and CRE infections, and neither has been studied in a trial dedicated to CRAB. For the first two generations this gap follows directly from the chemistry. Vaborbactam is essentially inactive against the OXA carbapenemases of *A. baumannii* (IC_50_ 120 µM against OXA-23) [10], and taniborbactam, although it inhibits the class D enzymes OXA-10 (IC_50_ 0.234 µM) and OXA-48 (IC_50_ 0.537 µM) [11], does not inhibit the OXA carbapenemases of *A. baumannii* at all [5,10]. For xeruborbactam, however, that explanation no longer holds. It inhibits OXA-23, OXA-24, and OXA-58, with IC_50_ values of 1 to 2 nM and dissociation constants of 0.58 to 3.2 nM, and inactivates them with second-order rate constants of approximately 1 × 10^6^ M^−1^·s^−1^ [10]—an affinity that the same study describes as comparable to that of durlobactam for these enzymes. This is a biochemical comparison only. Potent inhibition of purified OXA-23 does not by itself predict a clinically effective xeruborbactam-containing regimen for CRAB: partner-β-lactam permeability and PBP affinity in A. baumannii, AdeIJK-mediated efflux, the inhibitor’s own intrinsic antibacterial activity (MICs of 16–32 µg/mL), and PK/PD targets in critically ill patients all remain untested, and no boronate-containing regimen has been evaluated in CRAB infection [22]. The obstacle to a boronate-based option in CRAB infection is therefore no longer target engagement but clinical development: what remains is to identify a partner β-lactam, a dosing strategy, and a trial design.

By contrast, the DBO inhibitor durlobactam has demonstrated the clinical feasibility of targeting OXA-type carbapenemases. In the ATTACK trial, among the 125 patients in the microbiological ITT population with carbapenem-resistant *Acinetobacter baumannii-calcoaceticus* complex (ABC) infections, 28-day all-cause mortality was 19% (12/63) with sulbactam-durlobactam versus 32.3% (20/62) with colistin (difference, −13.2%; 95% CI, −30.0% to 3.5%), meeting the prespecified 20% non-inferiority margin [47]. Clinical cure rates were 62% versus 40% (difference, 22%; 95% CI, 2.9% to 40.3%). Molecular characterization of the ABC isolates from ATTACK by Miller et al. showed that efficacy tracked closely with the baseline OXA enzyme content of the isolates [48].

This comparison does not show that one drug is superior; it shows a natural division of labor between two chemical platforms. Modifications at the C2 methyl and C6 sulfate positions of the DBO scaffold give durlobactam a k_inact_/K_i_ of 5.1 × 10^3^ M^−1^·s^−1^ against OXA-23. Although about two orders of magnitude lower than the value against AmpC (~1 × 10^6^ M^−1^·s^−1^), this is sufficient to protect the intrinsic antibacterial activity of sulbactam in vivo [42]. In global surveillance, Karlowsky et al. reported MIC_50_ and MIC_90_ values of 1 and 2 µg/mL for sulbactam-durlobactam against 5032 clinical ABC isolates, with 98.3% of ABC isolates and 96.9% of CRAB isolates inhibited at the 4 µg/mL breakpoint [52]. This division of labor carries a cost, however: the sulbactam dose used in ATTACK was 4 g daily, well above conventional dosing, and cumulative neurotoxicity is a particular concern in patients with renal impairment. Durlobactam is a broad-spectrum DBO that inhibits class A, C, and D serine β-lactamases (kinact/Ki up to 1.4 × 10^7^ M^−1^·s^−1^ for TEM-1; ~10^6^ M^−1^·s^−1^ for KPC-2, AmpC-family cephalosporinases, and ADC-7), but it does not inhibit class B metallo-β-lactamases [42]. Its differentiating feature relative to avibactam is potent, reversible inhibition of the class D carbapenemases OXA-23, OXA-24/40, and OXA-58 (kinact/Ki 5.1 × 10^3^–2.5 × 10^5^ M^−1^·s^−1^) [42]. Accordingly, a systematic review found that all MBL-producing ABC isolates were resistant to sulbactam–durlobactam and that 12.5% of resistant isolates produced NDM-1, indicating that such infections should not be treated with this combination [53].

Earlier boronates could not close this gap, the monocyclic scaffold of vaborbactam and the first-generation bicyclic scaffold of taniborbactam are both poorly matched to the OXA carbapenemases of *A. baumannii*. Xeruborbactam has now cleared that chemical hurdle, as the nanomolar OXA-23/24/58 inhibition above shows; the barriers that remain for a boronate-based CRAB option are the clinical ones listed above rather than target engagement.

First-in-human phase 1 data for xeruborbactam are now available [39]. In 52 healthy adults (39 active drug, 13 placebo), intravenous xeruborbactam (single doses of 250–2000 mg. Multiple-dose regimens up to 1000 mg/day) showed dose-dependent exposure of total and unbound drug, a terminal half-life of 26.8–32.0 h (total) and 20.7–22.4 h (unbound), renal elimination as the principal clearance route (71.5–85.0% of the dose recovered in urine), concentration-dependent plasma protein binding (mean 88%), and accumulation ratios of 5.0–6.1 with 8-hourly dosing. Doses up to 1000 mg/day for 7–10 days were well tolerated; the most common adverse event was an asymptomatic, self-resolving alanine aminotransferase elevation, and no serious adverse events occurred. These data define the exposure targets that partner-β-lactam selection and dose optimization must now meet.

ABC, *Acinetobacter baumannii-calcoaceticus* complex; CI, confidence interval; CRE, carbapenem-resistant Enterobacterales; cUTI, complicated urinary tract infection; ESBL, extended-spectrum β-lactamase; mCRE-MITT, microbiological carbapenem-resistant Enterobacterales modified intention-to-treat; micro-ITT, microbiological intention-to-treat; q8h, every 8 h.

### 6.4. Safety and Tolerability

Across the pivotal trials, the boronate combinations show safety profiles dominated by class effects of the partner β-lactam. In TANGO I, adverse-event rates with meropenem–vaborbactam were comparable to those with piperacillin–tazobactam [43]; in TANGO II, the profile was consistent with meropenem alone [44]. In CERTAIN-1, adverse events occurred in 35.5% of cefepime–taniborbactam patients versus 29.0% with meropenem. The most frequent were headache (6.1%), diarrhea (4.1%), constipation (3.2%), hypertension (2.3%), and nausea (2.0%), serious adverse events were similar between groups (2.0% vs. 1.8%), and no case met Hy’s-law criteria [45]. In ATTACK, sulbactam–durlobactam was better tolerated than colistin, with substantially less nephrotoxicity [47]. In the first-in-human xeruborbactam study, doses up to 1000 mg/day for 7–10 days were well tolerated, with no serious adverse events; the most common finding was a mild, asymptomatic ALT elevation without bilirubin increase [39]. All boronate inhibitors are eliminated predominantly unchanged by renal excretion, and dose adjustment in renal impairment is required. Formal drug–drug interaction studies for taniborbactam and xeruborbactam are pending, although clinically significant interactions have not been reported to date.

## 7. Enzyme-Guided Precision Therapy

The complementary spectra of the approved boronate combination (meropenem-vaborbactam) and the approved DBO combination (sulbactam-durlobactam) make enzyme-guided precision therapy possible. In KPC-endemic regions (the United States, southern Europe, and parts of China), meropenem-vaborbactam is a reasonable choice, provided that rapid carbapenemase identification excludes co-production of OXA-48-like enzymes or MBLs. Where OXA-producing CRAB predominates in the ICU, sulbactam-durlobactam is currently the only approved targeted option (ATTACK trial, Table 4), but dosing must be individualized in renal impairment to avoid cumulative neurotoxicity from high-dose sulbactam. In VIM-endemic regions (Greece, Italy), cefepime-taniborbactam would be a reasonable choice once approved, with attention to its reduced activity where IMP enzymes are prevalent. In NDM-endemic regions (South Asia, the Middle East, and the Balkans), no single β-lactam/BLI regimen is reliable, and alternatives such as ceftazidime-avibactam plus aztreonam, or cefiderocol, may be considered, guided by local susceptibility data and rapid carbapenemase identification.

Evidence levels are defined by the authors for the purpose of this review: I, supported by randomized controlled trial data; II, supported by observational or non-randomized clinical data; III, supported only by preclinical data or expert opinion. These levels are not a validated grading scale. This table summarizes the authors’ interpretation of the evidence cited in this review. It is not a treatment guideline, and all options are off-label unless regulatory approval is indicated. ‘Approved’ refers to regulatory authorization of the combination in at least one jurisdiction for the indication shown; ‘investigational’ refers to combinations not yet authorized in any jurisdiction. Clinicians should consult local susceptibility data, institutional guidelines, and regional epidemiology before selecting therapy.

The main obstacle to enzyme-guided precision therapy is the lag in carbapenemase identification. In current practice, antibiotic selection for CR-GNB infection still relies largely on empirical treatment, and the uptake of rapid carbapenemase testing remains well below expectations, so that differences in spectrum between agents often lose their value before results become available. The technology is not lacking: Dortet et al. reported a sensitivity above 90% for the Carbapenem Detection Set in detecting carbapenemase-producing Enterobacterales [54], and Cordovana et al. described a complete MALDI-TOF MS workflow for detecting plasmid-encoded KPC-producing *K. pneumoniae* [55]. A more difficult problem is the multiplicity of the enzymes themselves: Qiao et al. found *bla*IMP-4, *bla*NDM-1, and *bla*OXA-1 coexisting with *bla*KPC-2 in a single clinical isolate of *Citrobacter freundii* in China [56], indicating that carriage of multiple carbapenemases is more common than previously assumed and that the one-enzyme-one-drug decision logic must be revised accordingly (Table 4). Genotype-stratified trial design. No formal sample-size calculation exists for a genotype-stratified trial of a pan-class BLI. As an orientation, assuming a 10–15 percentage-point difference in composite success between enzyme subgroups, a trial with three to four prespecified strata (KPC; NDM/VIM; OXA-48-like; IMP) would require on the order of 80–120 microbiologically evaluable patients per stratum, several hundred patients in total, likely necessitating a platform or master-protocol design with region-enriched recruitment. If such a trial were designed today, we would propose enrollment stratified by baseline carbapenemase genotype with prespecified per-stratum analyses, a comparator chosen by regional epidemiology, and an extended-microITT population enriched for meropenem-resistant pathogens, CERTAIN-1 randomized only 10 such patients in a 661-patient trial. We present this as an authorial perspective.

## 8. Conclusions

Over roughly a decade the boronic acid β-lactamase inhibitors have moved from a monocyclic scaffold active mainly against KPC to bicyclic compounds that inhibit serine enzymes and metallo-β-lactamases at nanomolar concentrations. With xeruborbactam, the class now reaches the OXA carbapenemases of *Acinetobacter baumannii* at an affinity comparable to durlobactam [32]. One boundary remains, however: the scaffold excludes the subclass B2 and B3 enzymes CphA and L1, and clinical validation is still pending. Vaborbactam proved the chemistry could work; taniborbactam extended it across serine and metallo enzymes; xeruborbactam has begun closing the long-standing gap in IMP coverage. Clinical development lags behind. Only meropenem-vaborbactam is approved. Cefepime-taniborbactam has completed phase 3 evaluation. Xeruborbactam remains earlier in development with no confirmed partner β-lactam, and no trial in this class has been stratified by carbapenemase genotype.

The partner β-lactam decides the spectrum of the finished product as much as the inhibitor does. Pairing a pan-class inhibitor with a β-lactam that cannot penetrate the organism, or has no activity against it, will not produce pan-class coverage at the bedside. Xeruborbactam also inhibits penicillin-binding proteins directly, which raises the risk of selecting resistance against the inhibitor itself if it is used with a weak partner drug. Finding a workable partner and dosing regimen matters more to its future than any further broadening of its spectrum.

Reported inhibition constants for the same compound against the same enzyme differ by one to two orders of magnitude between laboratories, reflecting differences in substrate, enzyme preparation, and pre-incubation conditions rather than real differences in potency. Rankings built from single-laboratory data, or comparisons mixing data from different labs, are not reliable until testing is done against a common enzyme panel under standardized conditions.

Every registrational trial in this class has been designed around a clinical syndrome, not an enzyme. The case for enzyme-guided therapy rests almost entirely on TANGO II, a trial of 77 patients dominated by KPC producers; no boronate has been evaluated in a trial dedicated to carbapenem-resistant A. baumannii, and conclusions about compounds that have not completed phase 3 remain provisional. A trial that cannot distinguish a KPC infection from an NDM infection cannot test whether a pan-class inhibitor delivers pan-class benefit. Future trials should stratify by baseline carbapenemase genotype, even if that slows recruitment.

Both taniborbactam and xeruborbactam fail against CphA and L1, the subclass B2 and B3 enzymes. This is a limitation of the bicyclic boronate core, not of any particular side chain, and closing it will likely require a different scaffold or multidentate chelation of the zinc centers rather than further optimization of the current chemistry. Rapid carbapenemase identification would also need to become routine practice before the spectrum differences between these agents could guide a clinician’s choice; without it, empirical selection continues regardless of what the inhibitors can do in vitro.

Selection experiments with meropenem-vaborbactam produced mutants with porin loss and enzyme overexpression, not active-site substitutions. These mechanisms act on drug entry and efflux rather than the target, and a broader inhibitor cannot address them. Surveillance for reduced susceptibility to these combinations should start now, while they remain rarely used clinically, rather than be reconstructed retrospectively once resistance has emerged.

## Figures and Tables

**Figure 1:**
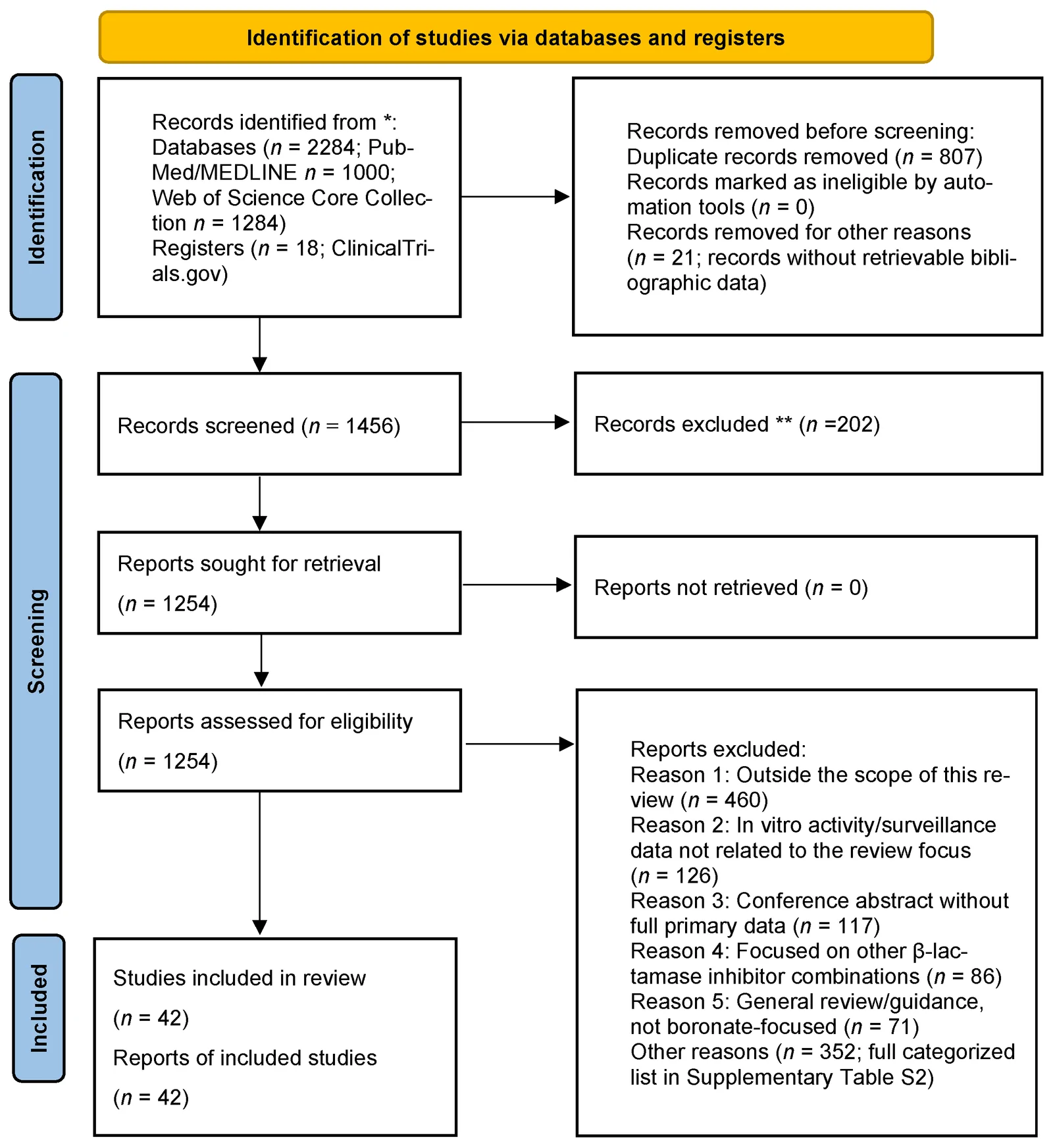
PRISMA 2020 flow diagram documenting the identification, screening, and selection of studies. Searches were conducted in PubMed/MEDLINE and Web of Science Core Collection (last searched 30 September 2026), with 18 additional trial registrations identified in ClinicalTrials.gov. No automation tools were used; screening was performed by title and abstract, and all exclusions were verified by the authors. Fourteen further reports were identified through citation searching of reference lists (PRISMA “other methods”) and are cited in the review. The complete list of the 1212 reports excluded at full-text assessment, with reasons, is provided in Supplementary Table S2. Adapted from Page MJ et al. BMJ 2021;372:n71 (CC BY 4.0) [14]. * Consider, if feasible, doing so, reporting the number of records identified from each database or register searched (rather than the total number across all databases/registers). ** If automation tools were used, indicate how many records were excluded by a human and how many were excluded by automation tools.

**Figure 2:**
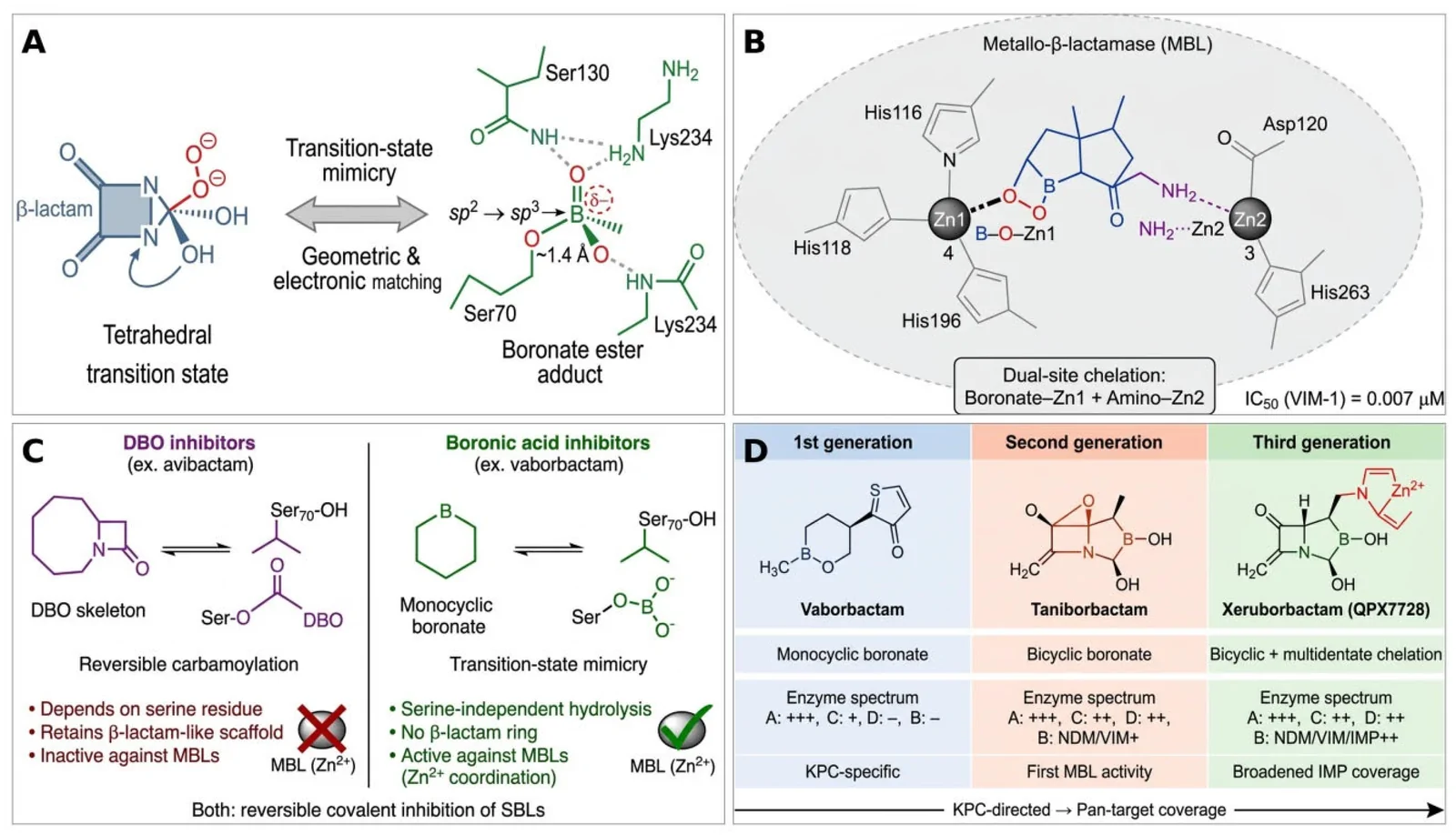
(**A**) The boronic acid group forms a 1.4-Å B–O covalent bond with the hydroxyl oxygen of Ser70, and its carboxamide group makes a hydrogen-bond network (dashed lines) with Ser130, Thr235, and Thr237. The 2-thiopheneacetyl side chain is buried in a hydrophobic pocket formed by Trp105, Cys69, and Pro107. (**B**) One boronate oxygen bridges Zn1 and Zn2 (1.9 Å to Zn1, 3.0 Å to Zn2); His116, His118, and His196 coordinate Zn1, whereas Asp120, Cys221, and His263 coordinate Zn2. (**C**) DBOs bind covalently by carbamoylating the catalytic serine, retain a strained cyclic urea that acts as a β-lactam mimic, and are intrinsically inactive against MBLs (Zn^2+^). Boronates do not depend on a β-lactam ring, and their boronate adduct is reversible but is not hydrolyzed, permitting MBL inhibition through Zn^2+^ coordination. The green check mark indicates that boronic acid inhibitors are active against metallo-β-lactamases (MBLs, via Zn^2+^ coordination), whereas the red cross indicates that DBO inhibitors are inactive against MBLs. (**D**) Progression from a monocyclic boronate (KPC-selective) to a bicyclic boronate (the first with MBL activity) and then to a bicyclic scaffold with multidentate coordination (optimized IMP coverage): increasing scaffold complexity parallels the expansion of the inhibitory spectrum from KPC-targeted to pan-class coverage. “+++”, “++”, “+”, and “−“ denote strong, moderate, weak, and no inhibitory activity, respectively, against the indicated Ambler class (A, C, and D: serine β-lactamases; B: metallo-β-lactamases), with relevant enzymes named where applicable. Letters indicate the Ambler enzyme classes.

**Table 1:** Comparative biochemical and microbiological activity of the boronic acid β-lactamase inhibitors.

Property	Vaborbactam	Taniborbactam	Xeruborbactam (QPX7728)
Scaffold	Monocyclic cyclic boronate	Bicyclic boronate	Bicyclic boronate with an additional Zn^2+^-coordinating group
Partner β-lactam	Meropenem	Cefepime	Meropenem and other β-lactams (investigational)
Class A, KPC-2	K_i_ 0.069 µM [19]; IC_50_ 110 nM [10]; 0.1 µM [15]	IC_50_ 0.03 µM [15]	IC_50_ 2.9 nM; K_d_ 0.25 nM; k_2_/K 3.6 × 10^5^ M^−1^·s^−1^; residence time 189 min [10]
Class A, ESBLs	K_i_ 0.029–0.110 µM [19]; IC_50_ 56–470 nM [10]; SHV-5 0.44, CTX-M-15 0.42 µM [15]	SHV-5 IC_50_ 0.0004 µM; CTX-M-15 0.01 µM [15]; TEM-116 0.12 µM [11]; 97.6% of carbapenemase-producing Enterobacterales inhibited at ≤8/4 mg/L [31]	IC_50_ 0.94–3.0 nM (CTX-M-14/15, SHV-12, TEM-10/43) [10]
Class C, AmpC	P99: K_i_ 0.053 µM [19]; IC_50_ 88 nM [10]; CMY-2 0.22 µM [15]	P99 IC_50_ 0.03 µM; CMY-2 0.007 µM [15]; *P. aeruginosa* AmpC 0.301 µM [11]	P99: IC_50_ 22 nM; K_d_ 0.53 nM [10]
Class D, OXA-48 and narrow-spectrum OXA	OXA-48 IC_50_ 6.9 µM [10], 38.8 µM [15]; OXA-1 7.9 µM [15]; no enhancement of meropenem against OXA-48-like CRE (MIC_50_/_90_ 16/≥32 µg/mL) [24]	OXA-48 IC_50_ 0.42 µM [15], 0.537 µM [11]; OXA-1 0.16 µM [15]; OXA-10 0.234 µM [11]; all OXA-48 producers susceptible at ≤8/4 mg/L [31]	OXA-48 IC_50_ 1.1 nM; K_d_ 0.13 nM [10]
Class D, OXA-23/24/58 (*A. baumannii*)	OXA-23 IC_50_ 120 µM [10]	No inhibition [5,10]	IC_50_ 1.0–2.0 nM; K_d_ 0.58–3.2 nM; k_2_/K ~1 × 10^6^ M^−1^·s^−1^ [10]
Class B1, NDM-1	IC_50_ > 160 µM [10], >100 µM [15]	IC_50_ 0.01 µM [11]; 0.19 µM [15] ^b^	IC_50_ 55 nM; K_i_ 32 nM [10] ^a^
Class B1, NDM-9 (E152K)	No activity	IC_50_ > 60 µM [5]	IC_50_ ~1.2 µM [33] ^a^
Class B1, VIM-1/VIM-2	IC_50_ > 160 µM [10], >100 µM [15]	IC_50_ 0.0079/0.0005 µM [11]; VIM-2 0.026 µM [15] ^b^	VIM-1: IC_50_ 14 nM; K_i_ 7.5 nM [10]
Class B1, IMP-1	IC_50_ > 160 µM [10], >100 µM [15]	IC_50_ 2.51 µM [11]; 39.8 µM [15] ^b^	IC_50_ 0.61 µM; K_i_ 0.24 µM [10]
Class B1, IMP-26	IC_50_ > 160 µM [10]	NR	IC_50_ 4.1 µM [10]
Class B1, SPM-1	No activity	More active than xeruborbactam [32]	No potentiation of β-lactams against SPM-1 producers [5]
Class B2, CphA	No activity	IC_50_ 2.51 µM [11]	NR
Class B3, L1	No activity	IC_50_ > 10 µM [11]	No potentiation of β-lactams against L1 producers [5]
MIC_90_ of the combination against CRE	1 µg/mL against KPC producers (meropenem plus vaborbactam at 8 µg/mL) [23]	16 µg/mL against MBL-positive isolates [32]; cefepime MIC_50_/_90_ 1/4 µg/mL against 14 MBL producers [15]	1 µg/mL against MBL-positive isolates (meropenem plus xeruborbactam at 8 µg/mL) [32]
Intrinsic antibacterial activity	NR	None (MIC > 128 µg/mL) [15]	MIC 16–32 µg/mL against *E. coli* and *K. pneumoniae* via PBP binding [22]
PK/PD driver	NR	%*f*T > C_t_; 40–50% for stasis and 60–100% for 1-log_10_ kill in a neutropenic murine thigh model; plasma protein binding 19.6% [38]	NR
Development stage	Approved (2017)	NDA submitted; phase 3 completed (NCT03840148) [15]	Phase 1 completed [39]; phase 2 ongoing [37]

The spectrum of xeruborbactam nonetheless has defined limits, and those limits are informative. Within the IMP family, potency varies by almost an order of magnitude, from IC_50_ 0.61 µM against IMP-1 to 4.1 µM against IMP-26 [10]. More fundamentally, xeruborbactam does not restore β-lactam activity against strains producing SPM-1 or L1, the subclass B3 enzyme of *Stenotrophomonas maltophilia* [6]. Docking analysis attributes the loss of affinity for L1 to two features: the absence of a positively charged residue equivalent to K211 of NDM-1, which removes the salt bridge formed by the inhibitor carboxylate, and truncation of the active-site capping loop, which removes the lipophilic contacts made by the phenyl and cyclopropyl rings [6]. Taniborbactam shares this blind spot, with IC_50_ values of 2.51 µM against the subclass B2 enzyme CphA and >10 µM against L1 [11]. That both compounds fail against the same enzymes indicates that it is the bicyclic boronate scaffold itself, rather than any particular side chain, that is not yet matched to the B2 and B3 active sites. CRE, carbapenem-resistant Enterobacterales; C_t_, concentration threshold; ESBL, extended-spectrum β-lactamase. FT, time above threshold for free drug; IC_50_, half-maximal inhibitory concentration; K_d_, dissociation constant; K_i_, inhibition constant. K_2_/K, second-order inactivation constant; MBL, metallo-β-lactamase; MIC, minimum inhibitory concentration; NDA, new drug application; NR, not reported in the sources cited in this review; PBP, penicillin-binding protein; PK/PD, pharmacokinetic/pharmacodynamic. Values obtained within a single study are internally comparable; comparisons across references should take account of differing substrates, enzyme preparations, and pre-incubation conditions. Class D values from reference [11] were also determined in the presence of 100 mM bicarbonate, which lowers apparent potency. ^a^ Reference [33] reports a higher IC_50_ for xeruborbactam against NDM-1 (0.77 µM) than reference [10] (55 nM). The NDM-1 and NDM-9 values from reference [33] should be compared with each other rather than with values from reference [10]. ^b^ Reported potencies of taniborbactam against subclass B1 MBLs differ by one to two orders of magnitude between references [11,15], which used different substrates and assay formats. Both are given so that the uncertainty is explicit. NR, not reported in the sources cited in this review. CphA is a chromosomal, mono-zinc subclass B2 MBL of Aeromonas hydrophila; L1 is a tetrameric, di-zinc subclass B3 MBL of *Stenotrophomonas maltophilia*. Neither is currently a major clinical target, but together they mark the boundary of the bicyclic boronate scaffold.

**Table 2:** Binding chemistry and spectrum of enzyme inhibition: DBOs versus boronic acids.

Feature	DBOs	Boronic Acids
Binding mechanism	Serine carbamoylation (covalent, reversible)	Transition-state mimicry plus Zn^2+^ coordination
Dependence on β-lactam ring	Dependent	Independent
Inhibition of SBLs	Classes A/C/D	Classes A/C/D
Inhibition of MBLs	None	Yes (structure-dependent)
Resistance mutation risk	KPC-2 variants (D179Y, etc.)	NDM-9 variants (E152K, etc.)
Representative drugs	Avibactam, relebactam, durlobactam	Vaborbactam, taniborbactam, xeruborbactam

**Table 3:** Design, principal results, and limitations of the pivotal phase 3 trials of the β-lactam/BLI combinations discussed in this review.

References	Design and Population	Regimen Versus Comparator	Primary EndPoint	Key Result	Main Limitation for the Present Review
[43]	Phase 3 randomized trial; 550 adults with cUTI	Meropenem-vaborbactam versus piperacillin-tazobactam	FDA composite endpoint	98.4% versus 94.0% (difference, 4.5%; *p* < 0.001); non-inferiority met	Only ~12% of baseline pathogens resistant to piperacillin-tazobactam; population largely ESBL rather than KPC, so KPC inhibition was not tested
[44]	Phase 3 randomized trial; 77 adults with CRE infection	Meropenem-vaborbactam monotherapy versus best available therapy	Clinical cure in the mCRE-MITT population	Cure 65.6% versus 33.3% (difference, 32.3%; 95% CI, 3.3 to 61.3); 28-day mortality 15.6% versus 33.3%	Small sample; dominated by KPC producers; minimal evidence for OXA-48-like or MBL producers
[45,46]	Phase 3 randomized trial; 661 adults with cUTI, 2:1 allocation	Cefepime-taniborbactam 2 g/0.5 g q8h versus meropenem 1 g q8h	Composite endpoint at follow-up in the micro-ITT population	70.6% (207/293) versus 58.0% (83/143) (difference, 12.6%; 95% CI, 3.1 to 22.2); superiority met	Only 10 patients carried meropenem-resistant pathogens; benefit driven by ESBL coverage rather than anti-CRE activity
[47,48]	Phase 3 randomized trial; 125 patients (micro-ITT) with carbapenem-resistant ABC infection	Sulbactam-durlobactam versus colistin	28-day all-cause mortality	19% (12/63) versus 32.3% (20/62) (difference, −13.2%; 95% CI, −30.0 to 3.5); non-inferiority met; cure 62% versus 40%	Sulbactam dosed at 4 g daily, above conventional dosing; MBL-producing isolates uniformly resistant; colistin comparator of limited efficacy and tolerability

**Table 4:** Potential treatment options by carbapenemase type and current level of supporting clinical evidence.

Carbapenemase Type	Option with Strongest Supporting Clinical Evidence	Other Options (Investigational or Lower-Level Evidence)	Notes and Limitations
KPC	Ceftazidime–avibactam (I)	Meropenem–vaborbactam (I); cefiderocol (II); cefepime–taniborbactam (investigational)	Vaborbactam appears less affected by resistance-conferring KPC-2 mutations than avibactam; exclude OXA-48-like/MBL co-production
OXA-48-like	Ceftazidime–avibactam (I)	Cefiderocol (II); aztreonam plus ceftazidime–avibactam (II); cefepime–taniborbactam (investigational)	Meropenem–vaborbactam is not active as monotherapy against OXA-48-like producers
NDM/VIM	Aztreonam plus ceftazidime–avibactam (II)	Cefiderocol (II); cefepime–taniborbactam (investigational); xeruborbactam combinations (preclinical)	No single reliable β-lactam/BLI regimen; combination therapy often advised; taniborbactam retains activity against most NDM/VIM but with a defined IMP gap
IMP	Aztreonam plus ceftazidime–avibactam (II)	Cefiderocol (II); xeruborbactam combinations (preclinical)	Taniborbactam has a substantial in vitro activity gap against IMP-type enzymes (see Section 4.2)
CRAB (OXA-23/24/40)	Sulbactam–durlobactam (I; approved for HABP/VABP due to ABC)	High-dose sulbactam-based regimens (II); cefiderocol (II)	MBL-producing CRAB isolates are resistant to sulbactam–durlobactam; high-dose sulbactam requires attention to cumulative neurotoxicity in renal impairment

## Data Availability

No new data were created or analyzed in this study. Data sharing is not applicable to this article.

## Supplementary Materials

The following supporting information can be downloaded at: https://www.mdpi.com/article/10.3390/molecules31203647/s1, Table S1: Database-specific search strategies and records identified (last searched 30 September 2026). Table S2: The 1212 reports excluded at full-text assessment, with reasons.
