# Supplementary material for: A Review on the Structural Evolution and Clinical Evidence of Boronic Acid β-Lactamase Inhibitors
Source: Molecules. 2026 Oct 13;31(20):3647. doi: 10.3390/molecules31203647 (PMC13707926; doi:10.3390/molecules31203647)
Supplement: Supplementary file 1 [file molecules-31-03647-s001.zip › molecules-4601772-supplementary.pdf]

Supplementary Materials

Zou, W.; Zhu, L. A Review on the Structural Evolution and Clinical Evidence of Boronic Acid β-Lactamase Inhibitors. Molecules 2026, manuscript molecules-4601772.

Table S1. Database-specific search strategies and records identified (last searched 30 September 2026).

Database or register (platform)	Search string	Records identified, n	
PubMed/MEDLINE (NCBI)	("Boronic Acids"[Mesh] OR "boronic acid"[Title/Abstract] OR "cyclic boronate"[Title/Abstract] OR "cyclic boronates"[Title/Abstract] OR vaborbactam[Title/Abstract] OR RPX7009[Title/Abstract] OR taniborbactam[Title/Abstract] OR VNRX-5133[Title/Abstract] OR xeruborbactam[Title/Abstract] OR QPX7728[Title/Abstract] OR durlobactam[Title/Abstract] OR ETX2514[Title/Abstract] OR ledaborbactam[Title/Abstract] OR VNRX-7145[Title/Abstract]) AND ("beta-Lactamases"[Mesh] OR "beta-lactamase inhibitor"[Title/Abstract] OR "beta-lactamase inhibitors"[Title/Abstract] OR carbapenemase[Title/Abstract] OR "metallo-beta-lactamase"[Title/Abstract])	1000	
Web of Science Core Collection (Clarivate)	TS=("boronic acid" OR "cyclic boronate*" OR vaborbactam OR RPX7009 OR taniborbactam OR VNRX-5133 OR xeruborbactam OR QPX7728 OR durlobactam OR ETX2514) AND TS=("beta-lactamase inhibitor*" OR carbapenemase* OR "metallo-beta-lactamase*")	1284	
ClinicalTrials.gov (register)	vaborbactam OR taniborbactam OR xeruborbactam OR QPX7728	18	
Protein Data Bank (RCSB)	vaborbactam / taniborbactam / xeruborbactam / QPX7728 (ligand search)	structures (reported qualitatively)	

Table S2. The 1212 reports excluded at full-text assessment, with reasons.

No.	Report title	Source database	Reason for exclusion
1	Design, synthesis, crystal structures, and antimicrobial activity of sulfonamide boronic acids as β-lactamase inhibitors	PubMed	In vitro activity or surveillance data
2	Carbapenemase-Producing Gram-Negative Bacteria in Andalusia, Spain, 2014-2018	PubMed	Other (outside the scope of this review)
3	Systematic Review and Meta-Analysis of Clinical Efficacy and Safety of Meropenem-Vaborbactam versus Best-Available Therapy in Patients with Carbapenem-Resistant Enterobacteriaceae Infections	PubMed	General review or guidance (not boronate-focused)
4	Cross-Border Emergence of Escherichia coli Producing the Carbapenemase NDM-5 in Switzerland and Germany	PubMed	Other (outside the scope of this review)
5	High transconjugation efficiency of fusion plasmid pNDM_KPC in carbapenem-resistant Citrobacter freundii and its formation driven by IS26-mediated integration	PubMed	Other (outside the scope of this review)
6	Emergence of resistance to novel β-lactam/β-lactamase inhibitor combinations in KPC-producing Klebsiella pneumoniae: clinical and genomic insights from consecutive bloodstream infections	PubMed	Resistance-mechanism or variant study
7	The complexed structure and antimicrobial activity of a non-beta-lactam inhibitor of AmpC beta-lactamase	PubMed	In vitro activity or surveillance data
8	NCY-1 β-Lactamase Activity Correlates With Antimicrobial Susceptibility of a Clinical Strain of Nocardia cyriacigeorgica	PubMed	In vitro activity or surveillance data
9	Impact of Acquired Broad-Spectrum β-Lactamases on Susceptibility to Cefiderocol and Newly Developed β-Lactam/β-Lactamase Inhibitor Combinations in Escherichia coli and Pseudomonas aeruginosa	PubMed	Focused on cefiderocol or other novel agents
10	Comparative activity of sulbactam and sulbactam/durlobactam against carbapenem-resistant A. baumannii isolates producing OXA-23 or OXA-23 plus PER-1 enzymes	PubMed	In vitro activity or surveillance data
11	In vitro activity of imipenem/relebactam, meropenem/vaborbactam, ceftazidime/avibactam, cefepime/zidebactam and other novel antibiotics against imipenem-non-susceptible Gram-negative bacilli from Taiwan	PubMed	Focused on other β-lactamase inhibitor combinations

12	Evaluating Antibacterial Efficacy of Cefiderocol and Cefepime/Taniborbactam Against OXA-48-Like and NDM-Expressing Enterobacterales: An In-Vitro and In-Silico Approach	PubMed	Focused on cefiderocol or other novel agents
13	Profiling interactions of vaborbactam with metallo-β-lactamases	PubMed	Other (outside the scope of this review)
14	Resistance to beta-lactam antibiotics: structure and mechanism based design of beta-lactamase inhibitors	PubMed	Structural or chemical-synthesis study
15	Activity of aztreonam-avibactam, cefiderocol, and cefepime-taniborbactam against a global collection of genetically characterized metallo-β-lactamase-producing Enterobacterales	PubMed	Focused on cefiderocol or other novel agents
16	A descriptive pharmacokinetic/pharmacodynamic analysis of continuous infusion meropenem/vaborbactam in the treatment of critically ill patients with documented KPC-producing Klebsiella pneumoniae ventilator-associated pneumonia	PubMed	PK/PD or pharmaceutical-technology study
17	NDM-1 (New Delhi metallo beta lactamase-1) producing Gram-negative bacilli: emergence & clinical implications	PubMed	Other (outside the scope of this review)
18	Characterization of IMI-4 carbapenemase-producing Enterobacter sp. isolated from humans and a companion animal in French Indian Ocean territories	PubMed	Other (outside the scope of this review)
19	Pharmacodynamics of taniborbactam in combination with cefepime studied in an in vitro model of infection	PubMed	PK/PD or pharmaceutical-technology study
20	The latest advances in β-lactam/β-lactamase inhibitor combinations for the treatment of Gram-negative bacterial infections	PubMed	Detection or diagnostic methodology
21	QPX7728, An Ultra-Broad-Spectrum B-Lactamase Inhibitor for Intravenous and Oral Therapy: Overview of Biochemical and Microbiological Characteristics	PubMed	General review or guidance (not boronate-focused)
22	NDM-9 resistance to taniborbactam	PubMed	Other (outside the scope of this review)
23	Dynamics and characterization of carbapenemase-producing Enterobacterales isolated from clinical specimens in a private healthcare center in Lima, Peru, from 2018 to 2019	PubMed	Other (outside the scope of this review)
24	Sulbactam-durlobactam: a β-lactam/β-lactamase inhibitor combination targeting Acinetobacter baumannii	PubMed	Other (outside the scope of this review)
25	New Antibiotics for Multidrug-Resistant Bacterial Strains: Latest Research Developments and Future	PubMed	General review or guidance (not boronate-focused)

	Perspectives		
26	In Vitro Activities of β-Lactam-β-Lactamase Inhibitor Antimicrobial Agents against Cystic Fibrosis Respiratory Pathogens	PubMed	Other (outside the scope of this review)
27	Structure-Based Development of (1-(3'-Mercaptopropanamido)methyl)boronic Acid Derived Broad-Spectrum, Dual-Action Inhibitors of Metallo- and Serine-β-lactamases	PubMed	Structural or chemical-synthesis study
28	Structure-based approach for binding site identification on AmpC beta-lactamase	PubMed	Structural or chemical-synthesis study
29	The Growing Threat of NDM-Producing Escherichia coli With Penicillin-Binding Protein 3 Mutations in the United States-Is There a Potential Role for Durlobactam?	PubMed	Other (outside the scope of this review)
30	Activity of Sulbactam-Durlobactam and Comparators Against a National Collection of Carbapenem-Resistant Acinetobacter baumannii Isolates From Greece	PubMed	In vitro activity or surveillance data
31	Activity of cefiderocol and innovative β-lactam/β-lactamase inhibitor combinations against isogenic strains of Escherichia coli expressing single and double β-lactamases under high and low permeability conditions	PubMed	Focused on cefiderocol or other novel agents
32	High-Throughput Crystallography Reveals Boron-Containing Inhibitors of a Penicillin-Binding Protein with Di- and Tricovalent Binding Modes	PubMed	Structural or chemical-synthesis study
33	Role of new antibiotics for KPC-producing Klebsiella pneumoniae	PubMed	Other (outside the scope of this review)
34	In vitro and in vivo activity of meropenem+avibactam against MBL-producing carbapenem-resistant Klebsiella pneumoniae	PubMed	In vitro activity or surveillance data
35	Antibiotics in development for multiresistant gram-negative bacilli	PubMed	Other (outside the scope of this review)
36	Antibacterial activity of enmetazobactam against Acinetobacter spp.: a molecular dissection of mechanism of action and resistance determinants	PubMed	Focused on other β-lactamase inhibitor combinations
37	First Report of Klebsiella pneumoniae-Carbapenemase-3-Producing Escherichia coli ST479 in Poland	PubMed	Other (outside the scope of this review)
38	New antimicrobial options for the management of complicated intra-abdominal infections	PubMed	Other (outside the scope of this review)
39	Antimicrobial susceptibility to last-resort antibiotics in carbapenemase-producing bacteria from Ukrainian patients	PubMed	In vitro activity or surveillance data
40	1,2,3-Triazolylmethaneboronate: A Structure Activity Relationship Study of a Class of β-Lactamase Inhibitors	PubMed	Structural or chemical-synthesis study

	against Acinetobacter baumannii Cephalosporinase		
41	Cyclic boronates as versatile scaffolds for KPC-2 β-lactamase inhibition	PubMed	Other (outside the scope of this review)
42	In vitro synergistic activity of meropenem/vaborbactam in combination with ceftazidime/avibactam against KPC-producing Klebsiella pneumoniae	PubMed	Combination-therapy study
43	In vitro and in vivo antibacterial activity of oral tebipenem in combination with novel β-lactamase inhibitors (avibactam, relebactam, vaborbactam) against KPC-producing Enterobacterales	PubMed	Combination-therapy study
44	Recent updates in treating carbapenem-resistant infections in patients with hematological malignancies	PubMed	General review or guidance (not boronate-focused)
45	Structural analysis of the boronic acid β-lactamase inhibitor vaborbactam binding to Pseudomonas aeruginosa penicillin-binding protein 3	PubMed	Other (outside the scope of this review)
46	Resistance to Ceftazidime/Avibactam plus Meropenem/Vaborbactam When Both Are Used Together Is Achieved in Four Steps in Metallo-β-Lactamase-Negative Klebsiella pneumoniae	PubMed	Other (outside the scope of this review)
47	Evaluation of the in vitro activity of WCK 5222 (cefepime/zidebactam) and currently available combination therapies against single- and double-carbapenemase producing Enterobacteriaceae: Expanding the zone of hope	PubMed	Focused on other β-lactamase inhibitor combinations
48	(19) F NMR Monitoring of Reversible Protein Post-Translational Modifications: Class D β-Lactamase Carbamylation and Inhibition	PubMed	Structural or chemical-synthesis study
49	Clinical data from studies involving novel antibiotics to treat multidrug-resistant Gram-negative bacterial infections	PubMed	Other (outside the scope of this review)
50	Comparative evaluation of combined-disk tests using different boronic acid compounds for detection of klebsiella pneumoniae carbapenemase-producing enterobacteriaceae clinical isolates	PubMed	Detection or diagnostic methodology
51	Purification of Staphylococcus aureus beta-lactamases by using sequential cation-exchange and affinity chromatography	PubMed	Other (outside the scope of this review)
52	Pharmacokinetics and safety of single and repeat doses of ceftibuten in healthy participants: a phase 1 dose escalation study	PubMed	PK/PD or pharmaceutical-technology study
53	Aztreonam-avibactam Demonstrates Potent Activity Against Carbapenem-resistant Enterobacterales Collected	PubMed	Focused on other β-lactamase inhibitor combinations

	From US Medical Centers Over a 6-year Period (2017-2022)		
54	From clinical trials to daily practice: how to adequately administer sulbactam-durlobactam? alone or combined with imipenem?	PubMed	Combination-therapy study
55	Impact of chromosomally encoded resistance mechanisms and transferable β-lactamases on the activity of cefiderocol and innovative β-lactam/β-lactamase inhibitor combinations against Pseudomonas aeruginosa	PubMed	Focused on cefiderocol or other novel agents
56	In vitro activity assessment of cefiderocol against Enterobacterales, Pseudomonas aeruginosa, and Acinetobacter spp., including β-lactam nonsusceptible molecularly characterized isolates, collected from 2020 to 2021 in the United States and European hospitals	PubMed	Focused on cefiderocol or other novel agents
57	Treatment strategies for imipenemase-producing Gram-negative infections: lessons from Japan	PubMed	Other (outside the scope of this review)
58	In vivo evolution of resistance to novel β-lactam/β-lactamase inhibitor combinations through overproduction of the horizontally acquired extended-spectrum AmpC β-lactamase FOX-14 and porin disruption in Serratia marcescens	PubMed	Resistance-mechanism or variant study
59	Interactions of "bora-penicilloates" with serine β-lactamases and DD-peptidases	PubMed	Other (outside the scope of this review)
60	Resistance to ceftazidime-avibactam and other new β-lactams in Pseudomonas aeruginosa clinical isolates: a multi-center surveillance study	PubMed	In vitro activity or surveillance data
61	Comparative biochemical and computational study of the role of naturally occurring mutations at Ambler positions 104 and 170 in GES β-lactamases	PubMed	Other (outside the scope of this review)
62	Purification of beta-lactamases by affinity chromatography on phenylboronic acid-agarose	PubMed	Other (outside the scope of this review)
63	Effects of the Val211Gly substitution on molecular dynamics of the CMY-2 cephalosporinase: implications on hydrolysis of expanded-spectrum cephalosporins	PubMed	Structural or chemical-synthesis study
64	[First isolation of KPC in Chile: from Italy to a public hospital in Santiago]	PubMed	Other (outside the scope of this review)
65	In vitro activity of cefiderocol against ceftazidime-avibactam susceptible and resistant KPC-producing Enterobacterales: cross-resistance and synergistic effects	PubMed	Focused on cefiderocol or other novel agents
66	Carbapenem-hydrolyzing gram-	PubMed	General review or

	negative bacteria: current options for treatment and review of drugs in development		guidance (not boronate-focused)
67	Synthesis and evaluation of boronic acids as inhibitors of Penicillin Binding Proteins of classes A, B and C	PubMed	Structural or chemical-synthesis study
68	The Development of New Small-Molecule Inhibitors Targeting Bacterial Metallo-β-lactamases	PubMed	Other (outside the scope of this review)
69	β-Lactam/β-Lactamase Inhibitor Combination Antibiotics Under Development	PubMed	Other (outside the scope of this review)
70	An Increase in the clinical isolation of acquired AmpC β-lactamase-producing Klebsiella pneumoniae in Korea from 2007 to 2010	PubMed	Other (outside the scope of this review)
71	Site-protected fixation and immobilization of Escherichia coli cells displaying surface-anchored beta-lactamase	PubMed	Other (outside the scope of this review)
72	In vitro evolution of durlobactam resistance in NDM-producing Escherichia coli due to a single mutation in mrdA encoding penicillin-binding protein 2	PubMed	Other (outside the scope of this review)
73	Novel KPC-2 Plasmid in a Clinical Salmonella Rissen Selected by Antibiotic Pressure	PubMed	Other (outside the scope of this review)
74	Deciphering the Evolution of Cephalosporin Resistance to Ceftolozane-Tazobactam in Pseudomonas aeruginosa	PubMed	Focused on other β-lactamase inhibitor combinations
75	Infectious Diseases Society of America 2022 Guidance on the Treatment of Extended-Spectrum β-lactamase Producing Enterobacterales (ESBL-E), Carbapenem-Resistant Enterobacterales (CRE), and Pseudomonas aeruginosa with Difficult-to-Treat Resistance (DTR-P. aeruginosa)	PubMed	General review or guidance (not boronate-focused)
76	Neutron and X-ray crystal structures of a perdeuterated enzyme inhibitor complex reveal the catalytic proton network of the Toho-1 β-lactamase for the acylation reaction	PubMed	Structural or chemical-synthesis study
77	Resistance to sulbactam/durlobactam in carbapenem-resistant Acinetobacter baumannii in treatment-naive setting: In vitro data and genomic insights from Italian bloodstream isolates	PubMed	Other (outside the scope of this review)
78	Multidrug-resistant gram-negative bacteria in Spanish ICU patients: clinical and microbiological characterization (MURAN-UCI Project)	PubMed	Other (outside the scope of this review)
79	Coexistence of bla(NDM-5), bla(CTX-M-15), bla(OXA-232), bla(SHV-182) genes in multidrug-resistant K. pneumoniae ST437-carrying OmpK36 and OmpK37 porin mutations: First report in Italy	PubMed	Other (outside the scope of this review)

80	Antimicrobial Activity of Ceftazidime-Avibactam and Comparators against Pathogens Harboring OXA-48 and AmpC Alone or in Combination with Other β-Lactamases Collected from Phase 3 Clinical Trials and an International Surveillance Program	PubMed	Combination-therapy study
81	In vitro activity of cefoxitin, imipenem, meropenem, and ceftaroline in combination with vaborbactam against Mycobacterium abscessus	PubMed	Mycobacterium-focused
82	Activity of Cefepime in Combination with the Novel β-Lactamase Inhibitor Taniborbactam (VNRX-5133) against Extended-Spectrum-β-Lactamase-Producing Isolates in In Vitro Checkerboard Assays	PubMed	Combination-therapy study
83	Assessment of Activity and Resistance Mechanisms to Cefepime in Combination with the Novel β-Lactamase Inhibitors Zidebactam, Taniborbactam, and Enmetazobactam against a Multicenter Collection of Carbapenemase-Producing Enterobacterales	PubMed	Focused on other β-lactamase inhibitor combinations
84	In vitro activity of imipenem/relebactam, meropenem/vaborbactam and comparators against Enterobacterales causing urinary tract infection in Taiwan: results from the Study for Monitoring Antimicrobial Resistance Trends (SMART), 2020	PubMed	Focused on other β-lactamase inhibitor combinations
85	Boronic Acid Transition State Inhibitors as Potent Inactivators of KPC and CTX-M β-Lactamases: Biochemical and Structural Analyses	PubMed	Other (outside the scope of this review)
86	Epidemiology and in vitro activity of ceftazidime-avibactam, meropenem-vaborbactam, imipenem-relebactam, eravacycline, plazomicin, and comparators against Greek carbapenemase-producing Klebsiella pneumoniae isolates	PubMed	Focused on other β-lactamase inhibitor combinations
87	Meropenem/vaborbactam activity in vitro: a new option for Klebsiella pneumoniae carbapenemase (KPC)-producing Klebsiella pneumoniae treatment	PubMed	Other (outside the scope of this review)
88	Novel Specific Metallo-β-Lactamase Inhibitor ANT2681 Restores Meropenem Activity to Clinically Effective Levels against NDM-Positive Enterobacterales	PubMed	Other (outside the scope of this review)
89	In vitro activity of the novel β-lactamase inhibitor taniborbactam (VNRX-5133), in combination with cefepime or meropenem, against MDR Gram-negative bacterial isolates from China	PubMed	Combination-therapy study

90	Potency of Vaborbactam Is Less Affected than That of Avibactam in Strains Producing KPC-2 Mutations That Confer Resistance to Ceftazidime-Avibactam	PubMed	Other (outside the scope of this review)
91	Novel Variant of New Delhi Metallo-Beta-Lactamase (bla(NDM-60)) Discovered in a Clinical Strain of Escherichia coli from the United Arab Emirates: An Emerging Challenge in Antimicrobial Resistance	PubMed	Other (outside the scope of this review)
92	Novel β-Lactam/β-Lactamase Inhibitors Versus Best Available Therapy on the Mortality-Related Carbapenem-Resistant Enterobacterales Infection: A Systematic Review and Meta-Analysis	PubMed	Clinical-outcomes or pharmacoeconomic study
93	New agents for the treatment of infections with Gram-negative bacteria: restoring the miracle or false dawn?	PubMed	Other (outside the scope of this review)
94	Rapid Flow Cytometry Test for Identification of Different Carbapenemases in Enterobacteriaceae	PubMed	Detection or diagnostic methodology
95	Antifungal agent tavaborole as a potential broad-spectrum serine and metallo-β-lactamases inhibitor	PubMed	Other (outside the scope of this review)
96	The role of new β-lactamase inhibitors in gram-negative infections	PubMed	Other (outside the scope of this review)
97	[New antibiotics: an overview]	PubMed	General review or guidance (not boronate-focused)
98	Cefepime 2.0: Upgrading β-Lactamase Inhibitor Use	PubMed	Other (outside the scope of this review)
99	Sulbactam-Durlobactam for Carbapenem-resistant Acinetobacter baumannii in India: Does Local Molecular Epidemiology Support Current Guideline Recommendations?	PubMed	General review or guidance (not boronate-focused)
100	Meropenem/Vaborbactam in Pediatrics: 2 Cases of CRE Intraabdominal Infection	PubMed	Other (outside the scope of this review)
101	New Antibiotics Against Multidrug-Resistant Gram-Negative Bacteria in Lung Transplantation: Clinical Evidence, Safety, and PK/PD Properties	PubMed	Other (outside the scope of this review)
102	Correction to: Epidemiology and in vitro activity of ceftazidime-avibactam, meropenem-vaborbactam, imipenem-relebactam, eravacycline, plazomicin, and comparators against Greek carbapenemase-producing Klebsiella pneumoniae isolates	PubMed	Correction, comment, or reply
103	Safety, Pharmacokinetics, and Drug-Drug Interaction Potential of Intravenous Durlobactam, a β-Lactamase Inhibitor, in Healthy Subjects	PubMed	PK/PD or pharmaceutical-technology study
104	Infections Caused by Carbapenem-Resistant Enterobacteriaceae: An Update on Therapeutic Options	PubMed	General review or guidance (not boronate-focused)

105	Mutations in ompK36 differentially impact in vitro synergy of meropenem/vaborbactam and ceftazidime/avibactam in combination with other antibiotics against KPC-producing Klebsiella pneumoniae	PubMed	Combination-therapy study
106	Pharmacokinetic drug evaluation of co-packaged sulbactam for injection and durlobactam for injection for the treatment of Acinetobacter baumannii-calcoaceticus complex in HABP/VABP	PubMed	PK/PD or pharmaceutical-technology study
107	Evaluation of sulbactam/durlobactam activity and synergy against highly drug-resistant Acinetobacter baumannii strains	PubMed	Combination-therapy study
108	Suboptimal ceftazidime-avibactam exposure drives sequential bla(KPC) mutations and intra-host coexistence of Klebsiella pneumoniae harboring distinct variants leading to persistent infection	PubMed	Other (outside the scope of this review)
109	Aza-boronic acids as non-beta-lactam inhibitors of AmpC-beta-lactamase	PubMed	Other (outside the scope of this review)
110	Crystal structures of KPC-2 β-lactamase in complex with 3-nitrophenyl boronic acid and the penam sulfone PSR-3-226	PubMed	Structural or chemical-synthesis study
111	Steady-state kinetics of the binding of beta-lactams and penicilloates to the second binding site of the Enterobacter cloacae P99 beta-lactamase	PubMed	Other (outside the scope of this review)
112	Identification of metallo-β-lactamases and AmpC production among Escherichia coli strains isolated from hemodialysis patients with urinary tract infection	PubMed	Other (outside the scope of this review)
113	Cefepime-Taniborbactam: A Novel Cephalosporin/β-Lactamase Inhibitor Combination	PubMed	Other (outside the scope of this review)
114	Comparative In Vitro Activity of Ceftazidime-Avibactam, Imipenem-Relebactam, and Meropenem-Vaborbactam against Carbapenem-Resistant Clinical Isolates of Klebsiella pneumoniae and Pseudomonas aeruginosa	PubMed	Focused on other β-lactamase inhibitor combinations
115	Quantification of sulbactam and durlobactam in saline and human plasma via ultra-performance liquid chromatography tandem mass spectrometry	PubMed	PK/PD or pharmaceutical-technology study
116	Boron-Containing Drug Candidates in Clinical Trials	PubMed	Other (outside the scope of this review)
117	Erratum to: In vitro activity of the novel β-lactamase inhibitor taniborbactam (VNRX-5133), in combination with cefepime or meropenem, against MDR Gram-negative bacterial isolates from China	PubMed	Correction, comment, or reply
118	I n vitro	PubMed	PK/PD or

	pharmacokinetics/pharmacodynamics of the β-lactamase inhibitor, durlobactam, in combination with sulbactam against Acinetobacter baumannii-calcoaceticus complex		pharmaceutical-technology study
119	AI-enabled discovery of small molecule β-lactamase inhibitors to overcome enzyme diversity and resistance evolution	PubMed	Other (outside the scope of this review)
120	Sulbactam-durlobactam susceptibility test method development and quality control ranges for MIC and disk diffusion tests	PubMed	In vitro activity or surveillance data
121	An overview of sulbactam-durlobactam approval and implications in advancing therapeutics for hospital-acquired and ventilator-associated pneumonia by acinetobacter baumannii-calcoaceticus complex: A narrative review	PubMed	General review or guidance (not boronate-focused)
122	Sulbactam-Durlobactam in the Treatment of Bloodstream Infection Due to Carbapenem-Resistant Acinetobacter baumannii: A Case Series and Literature Review	PubMed	Case report
123	Assessing Clinical Effectiveness of Ceftolozane/Tazobactam, Ceftazidime/Avibactam, and Meropenem/Vaborbactam in Bacteremia: A Systematic Review and Meta-Analysis	PubMed	Focused on other β-lactamase inhibitor combinations
124	Characterization of Carbapenemase-Producing Klebsiella pneumoniae Isolates from Two Romanian Hospitals Co-Presenting Resistance and Heteroresistance to Colistin	PubMed	Resistance-mechanism or variant study
125	Weighing the Odds: Novel β-Lactam/β-Lactamase Inhibitor Use in Hospital-Acquired and Ventilator-Associated Pseudomonas aeruginosa Pneumonia for Patients Who Are Morbidly Obese	PubMed	Other (outside the scope of this review)
126	Molecular resistance mechanisms to newly approved antibiotics (2017-2025) in WHO priority pathogens	PubMed	Other (outside the scope of this review)
127	Sulbactam for carbapenem-resistant Acinetobacter baumannii infections: a literature review	PubMed	General review or guidance (not boronate-focused)
128	Clinical Characteristics and Outcome of Ceftazidime/Avibactam-Resistant Klebsiella pneumoniae Carbapenemase-Producing Klebsiella pneumoniae Infections: A Retrospective, Observational, 2-Center Clinical Study	PubMed	Clinical-outcomes or pharmacoeconomic study
129	Ceftazidime-avibactam resistance in Klebsiella pneumoniae: A growing public health concern across diverse sublineages, 2019-2024	PubMed	Other (outside the scope of this review)
130	How I manage patients with New Delhi metallo-beta-lactamase and OXA-48-producing Enterobacterales infections:	PubMed	Other (outside the scope of this review)

	a practical approach		
131	A Systematic Review of the Pharmacokinetics and Pharmacodynamics of Novel Beta-Lactams and Beta-Lactam with Beta-Lactamase Inhibitor Combinations for the Treatment of Pneumonia Caused by Carbapenem-Resistant Gram-Negative Bacteria	PubMed	PK/PD or pharmaceutical-technology study
132	Cefepime-taniborbactam demonstrates potent in vitro activity vs Enterobacterales with bla(OXA-48)	PubMed	In vitro activity or surveillance data
133	Effect of modification of penicillin-binding protein 3 on susceptibility to ceftazidime-avibactam, imipenem-relebactam, meropenem-vaborbactam, aztreonam-avibactam, cefepime-taniborbactam, and cefiderocol of Escherichia coli strains producing broad-spectrum β-lactamases	PubMed	Focused on cefiderocol or other novel agents
134	Examining the activity of cefepime-taniborbactam against Burkholderia cepacia complex and Burkholderia gladioli isolated from cystic fibrosis patients in the United States	PubMed	In vitro activity or surveillance data
135	Synthesis of a Novel Boronic Acid Transition State Inhibitor, MB076: A Heterocyclic Triazole Effectively Inhibits Acinetobacter-Derived Cephalosporinase Variants with an Expanded-Substrate Spectrum	PubMed	Structural or chemical-synthesis study
136	Multidrug-resistant Enterobacterales in United States hospitals: Frequency and susceptibility to newer β-lactamase inhibitor combinations (2020-2024)	PubMed	In vitro activity or surveillance data
137	Overexpression of KPC contributes to ceftazidime-avibactam heteroresistance in clinical isolates of carbapenem-resistant Klebsiella pneumoniae	PubMed	Resistance-mechanism or variant study
138	Citrobacter freundii resistant to novel β-lactamase inhibitor combinations and cefiderocol, co-producing class A, B and D carbapenemases encoded by transferable plasmids	PubMed	Focused on cefiderocol or other novel agents
139	A Post-Neurosurgical Infection due to KPC-Producing Klebsiella pneumoniae Treated with Meropenem-Vaborbactam: A Case Report	PubMed	Case report
140	Mutation of kvrA Causes OmpK35 and OmpK36 Porin Downregulation and Reduced Meropenem-Vaborbactam Susceptibility in KPC-Producing Klebsiella pneumoniae	PubMed	In vitro activity or surveillance data
141	Activity of Meropenem-Vaborbactam against Bacterial Isolates Causing Pneumonia in Patients in U.S. Hospitals during 2014 to 2018	PubMed	In vitro activity or surveillance data
142	Therapeutic potential of boron-	PubMed	Other (outside the

	containing compounds		scope of this review)
143	Characterization of GUA-1, a chromosomally encoded ESBL from Pseudomonas guariconensis-like	PubMed	Other (outside the scope of this review)
144	Increased bla(KPC) Copy Number and OmpK35 and OmpK36 Porins Disruption Mediated Resistance to Imipenem/Relebactam and Meropenem/Vaborbactam in a KPC-Producing Klebsiella pneumoniae Clinical Isolate	PubMed	Focused on other β-lactamase inhibitor combinations
145	Meropenem/vaborbactam fixed combination for the treatment of patients with complicated urinary tract infections	PubMed	Other (outside the scope of this review)
146	Potency of Meropenem-Vaborbactam in Lung Surfactant	PubMed	Other (outside the scope of this review)
147	In vitro activity of flomoxef against extended-spectrum β-lactamase-producing Escherichia coli and Klebsiella pneumoniae in Korea	PubMed	In vitro activity or surveillance data
148	Beta-lactamase inhibitors. The inhibition of serine beta-lactamases by specific boronic acids	PubMed	Other (outside the scope of this review)
149	Genomic characterization of a Klebsiella pneumoniae ST1519 resistant to ceftazidime/avibactam carrying a novel KPC variant (KPC-36)	PubMed	Resistance-mechanism or variant study
150	Use of boronic acid disk methods to detect the combined expression of plasmid-mediated AmpC beta-lactamases and extended-spectrum beta-lactamases in clinical isolates of Klebsiella spp., Salmonella spp., and Proteus mirabilis	PubMed	Detection or diagnostic methodology
151	A boronic-acid-based probe for fluorescence polarization assays with penicillin binding proteins and β-lactamases	PubMed	Other (outside the scope of this review)
152	APC24-7, a covalent combination of boronic acid and chelator moieties, restores β-lactam efficiency against metallo-β-lactamase-producers	PubMed	Other (outside the scope of this review)
153	Molecular analysis of metallo-beta-lactamase-producing Pseudomonas aeruginosa in Switzerland 2022-2023	PubMed	Other (outside the scope of this review)
154	β-lactam/β-lactamase inhibitor combinations: from then to now	PubMed	Other (outside the scope of this review)
155	Utilization of Colistin Versus β-Lactam and β-Lactamase Inhibitor Agents in Relation to Acute Kidney Injury in Patients with Severe Gram-Negative Infections	PubMed	Other (outside the scope of this review)
156	Genotypic Evolution of Klebsiella pneumoniae Sequence Type 512 during Ceftazidime/Avibactam, Meropenem/Vaborbactam, and Cefiderocol Treatment, Italy	PubMed	Focused on cefiderocol or other novel agents
157	In vitro activity of imipenem-relebactam against KPC-producing	PubMed	Focused on other β-lactamase inhibitor

	Klebsiella pneumoniae resistant to ceftazidime-avibactam and/or meropenem-vaborbactam		combinations
158	Activity of aztreonam-avibactam against Enterobacterales resistant to recently approved beta-lactamase inhibitor combinations collected in Europe, Latin America, and the Asia-Pacific Region (2020-2022)	PubMed	Focused on other β-lactamase inhibitor combinations
159	Future alternatives for the treatment of infections caused by carbapenemase-producing Enterobacteriaceae: what is in the pipeline?	PubMed	General review or guidance (not boronate-focused)
160	Structure and mechanism of taniborbactam inhibition of the cefepime-hydrolyzing, partial R2-loop deletion Pseudomonas-derived cephalosporinase variant PDC-88	PubMed	Structural or chemical-synthesis study
161	Use of Aztreonam-Avibactam with Rapid Eravacycline Step-Down Therapy for a Tibial Septic Non-Union by NDM-Producing Enterobacter cloacae	PubMed	Focused on other β-lactamase inhibitor combinations
162	Combating Multidrug-Resistant Acinetobacter baumannii: Insights from Japan, with Global Relevance	PubMed	Other (outside the scope of this review)
163	Current treatment options for pneumonia caused by carbapenem-resistant Acinetobacter baumannii	PubMed	Other (outside the scope of this review)
164	Comparative activity of newer β-lactam/β-lactamase inhibitor combinations against Pseudomonas aeruginosa isolates from US medical centres (2020-2021)	PubMed	In vitro activity or surveillance data
165	Cefiderocol, a Siderophore Cephalosporin, as a Treatment Option for Infections Caused by Carbapenem-Resistant Enterobacterales	PubMed	Focused on cefiderocol or other novel agents
166	Treatment Options for Carbapenem-resistant Gram-negative Bacterial Infections	PubMed	Other (outside the scope of this review)
167	Impact of ompk36 genotype and KPC subtype on the in vitro activity of ceftazidime/avibactam, imipenem/relebactam and meropenem/vaborbactam against KPC-producing K. pneumoniae clinical isolates	PubMed	Focused on other β-lactamase inhibitor combinations
168	Indian Society of Critical Care Medicine Guidelines on Evidence-based Recommendations for the Treatment of Carbapenem-resistant Enterobacterales, Carbapenem-resistant Acinetobacter baumannii, and Carbapenem-resistant Pseudomonas aeruginosa in Critically Ill Patients: A GRADE-guided Approach	PubMed	General review or guidance (not boronate-focused)
169	Bacterial Infections	PubMed	Other (outside the scope of this review)
170	Heteroresistance to cefepime-	PubMed	Resistance-mechanism

	taniborbactam in metallo-β-lactamase-encoding Enterobacterales		or variant study
171	Early Experience With Meropenem-Vaborbactam for Treatment of Carbapenem-resistant Enterobacteriaceae Infections	PubMed	Other (outside the scope of this review)
172	Aztreonam-Avibactam Combination Restores Susceptibility of Aztreonam in Dual-Carbapenemase-Producing Enterobacteriaceae	PubMed	Focused on other β-lactamase inhibitor combinations
173	Diagnostic utility of boronic acid inhibition with different cephalosporins against Escherichia coli producing AmpC β-lactamases	PubMed	Other (outside the scope of this review)
174	Comparative outcomes of ceftazidime-avibactam versus meropenem-vaborbactam for KPC-producing Enterobacterales infections	PubMed	Clinical-outcomes or pharmacoeconomic study
175	New-generation antibiotics and nonantibiotic strategies against carbapenemase-producing Enterobacterales: More focus on metallo-β-lactamase producers	PubMed	Other (outside the scope of this review)
176	Rates of resistance and heteroresistance to newer β-lactam/β-lactamase inhibitors for carbapenem-resistant Enterobacterales	PubMed	Resistance-mechanism or variant study
177	In vitro activity of cefepime/zidebactam against sulbactam/durlobactam-susceptible and -resistant Acinetobacter baumannii clinical isolates	PubMed	Focused on other β-lactamase inhibitor combinations
178	Recognizing and Overcoming Resistance to New Beta-Lactam/Beta-Lactamase Inhibitor Combinations	PubMed	Other (outside the scope of this review)
179	Resistance of Acinetobacter baumannii Complex Clinical Isolates to Sulbactam-Durlobactam: A Systematic Review of Data from In Vitro Studies	PubMed	General review or guidance (not boronate-focused)
180	In vitro activity of sulbactam-durlobactam against carbapenem-resistant Acinetobacter baumannii and mechanisms of resistance	PubMed	In vitro activity or surveillance data
181	Cefepime-taniborbactam: Ushering in the era of metallo-β-lactamase inhibition	PubMed	Other (outside the scope of this review)
182	Draft genome sequence of a KPC-2-producing, meropenem-durlobactam non-susceptible Klebsiella pneumoniae ST11-KL64 clinical isolate	PubMed	Other (outside the scope of this review)
183	Correction to: Imipenem-Relebactam and Meropenem-Vaborbactam: Two Novel Carbapenem-β-Lactamase Inhibitor Combinations	PubMed	Correction, comment, or reply
184	Geographic variations in distributions of carbapenemase-encoding genes, susceptibilities, and minimum inhibitory concentrations of inpatient meropenem-resistant Enterobacterales to ceftazidime-avibactam, meropenem-	PubMed	Focused on other β-lactamase inhibitor combinations

	vaborbactam, and aztreonam-avibactam across four global regions: 2020-2022 data from the Antimicrobial Testing Leadership and Surveillance		
185	TROJAN-MDR: in vitro activity of cefiderocol and comparators against multidrug-resistant Enterobacterales and Pseudomonas aeruginosa strains in Southern France, evaluation of available testing methods performances	PubMed	Focused on cefiderocol or other novel agents
186	In vitro activity of ceftazidime-avibactam, ceftolozane-tazobactam, imipenem-relebactam, meropenem-vaborbactam and cefiderocol against carbapenemase-producing Enterobacterales from clinical isolates in a tertiary healthcare centre in Serbia	PubMed	Focused on cefiderocol or other novel agents
187	In vitro activity of cefiderocol against a global collection of carbapenem-resistant Pseudomonas aeruginosa with a high level of carbapenemase diversity	PubMed	Focused on cefiderocol or other novel agents
188	In Vitro Mechanisms of Resistance Development to Imipenem-Relebactam in KPC-Producing Klebsiella pneumoniae	PubMed	Focused on other β-lactamase inhibitor combinations
189	Imipenem/Relebactam Resistance in Clinical Isolates of Extensively Drug Resistant Pseudomonas aeruginosa: Inhibitor-Resistant β-Lactamases and Their Increasing Importance	PubMed	Focused on other β-lactamase inhibitor combinations
190	Beta-lactamase-catalyzed aminolysis of depsipeptides: proof of the nonexistence of a specific D-phenylalanine/enzyme complex by double-label isotope trapping	PubMed	Other (outside the scope of this review)
191	Ceftazidime/avibactam and eravacycline susceptibility of carbapenem-resistant Klebsiella pneumoniae in two Greek tertiary teaching hospitals	PubMed	In vitro activity or surveillance data
192	In vivo development of resistance to novel β-lactam/β-lactamase inhibitor combinations in KPC-producing Klebsiella pneumoniae infections: a case series	PubMed	Case report
193	Novel agents in development for the treatment of resistant Gram-negative infections	PubMed	Other (outside the scope of this review)
194	Susceptibility to new antibiotics and genetic characterisation of carbapenemase-producing Enterobacterales: low activity of cefiderocol against NDM-producing isolates	PubMed	Focused on cefiderocol or other novel agents
195	In vitro activity of zosurabalpin against a global set of carbapenem-resistant-A. baumannii clinical isolates	PubMed	In vitro activity or surveillance data
196	In vivo evolution of resistance to	PubMed	Resistance-mechanism

	contemporary β-lactam/β-lactamase inhibitor combinations during treatment of a KPC-producing Serratia marcescens infection		or variant study
197	Structural basis of metallo-β-lactamase resistance to taniborbactam	PubMed	Other (outside the scope of this review)
198	Vaborbactam increases meropenem susceptibility in Pseudomonas aeruginosa clinical isolates displaying MexXY and AmpC upregulation	PubMed	In vitro activity or surveillance data
199	Rapid meropenem/vaborbactam NP test for detecting susceptibility/resistance in Enterobacterales	PubMed	In vitro activity or surveillance data
200	In vitro activity of ceftazidime/avibactam, imipenem/relebactam and meropenem/vaborbactam alone or in combination with polymyxin B against carbapenem resistant Acinetobacter baumannii	PubMed	Focused on other β-lactamase inhibitor combinations
201	In vivo pharmacokinetic/pharmacodynamic evaluation of cefepime/taniborbactam combination against cefepime-non-susceptible Enterobacterales and Pseudomonas aeruginosa in a murine pneumonia model	PubMed	PK/PD or pharmaceutical-technology study
202	Amphiphilic nebramine analogs synergize with β-lactam/β-lactamase inhibitor combinations, including cefepime-taniborbactam and meropenem-xeruborbactam against metallo-β-lactamase-carrying Pseudomonas aeruginosa	PubMed	Other (outside the scope of this review)
203	Occurrence of multi-carbapenemases producers among carbapenemase-producing Enterobacterales and in vitro activity of combinations including cefiderocol, ceftazidime-avibactam, meropenem-vaborbactam, and aztreonam in the COVID-19 era	PubMed	Focused on cefiderocol or other novel agents
204	Meropenem-vaborbactam: a critical positioning for the management of infections by Carbapenem-resistant Enterobacteriaceae	PubMed	Other (outside the scope of this review)
205	Loss-of-Function Mutations in the Penicillin-Binding Protein PonA1 Confer Agar-Dependent Resistance to Durlobactam in Mycobacterium abscessus	PubMed	Mycobacterium-focused
206	Durlobactam in the Treatment of Multidrug-Resistant Acinetobacter baumannii Infections: A Systematic Review	PubMed	General review or guidance (not boronate-focused)
207	Boronic Acids as Prospective Inhibitors of Metallo-β-Lactamases: Efficient Chemical Reaction in the Enzymatic Active Site Revealed by Molecular Modeling	PubMed	Other (outside the scope of this review)

208	In vitro activity of the orally bioavailable ceftibuten/VNRX-7145 (VNRX-5236 etzadroxil) combination against a challenge set of Enterobacterales pathogens carrying molecularly characterized β-lactamase genes	PubMed	In vitro activity or surveillance data
209	Novel β-lactam-β-lactamase inhibitor combinations: expectations for the treatment of carbapenem-resistant Gram-negative pathogens	PubMed	Other (outside the scope of this review)
210	Structures of FOX-4 Cephamycinase in Complex with Transition-State Analog Inhibitors	PubMed	Structural or chemical-synthesis study
211	Critical reappraisal of current issues for improving the proper clinical use of the incoming beta-lactam/beta-lactamase inhibitor combinations of tomorrow	PubMed	General review or guidance (not boronate-focused)
212	[Beta-lactams and beta-lactamase inhibitors-current developments]	PubMed	Other (outside the scope of this review)
213	Antibiotics and non-traditional antimicrobial agents for Pseudomonas aeruginosa in clinical phases 1, 2, and 3 trials	PubMed	Other (outside the scope of this review)
214	In Vitro Susceptibility of Aztreonam-Vaborbactam, Aztreonam-Relebactam and Aztreonam-Avibactam Associations against Metallo-β-Lactamase-Producing Gram-Negative Bacteria	PubMed	Focused on other β-lactamase inhibitor combinations
215	Microbiological, Clinical, and PK/PD Features of the New Anti-Gram-Negative Antibiotics: β-Lactam/β-Lactamase Inhibitors in Combination and Cefiderocol-An All-Inclusive Guide for Clinicians	PubMed	Focused on cefiderocol or other novel agents
216	New β-lactamase inhibitors: a therapeutic renaissance in an MDR world	PubMed	General review or guidance (not boronate-focused)
217	Risk factors for KPC-producing Klebsiella pneumoniae enteric colonization upon ICU admission	PubMed	Other (outside the scope of this review)
218	Structural insights into the activity of carbapenemases: understanding the mechanism of action of current inhibitors and informing the design of new carbapenem adjuvants	PubMed	In vitro activity or surveillance data
219	The Next-Generation β-Lactamase Inhibitor Taniborbactam Restores the Morphological Effects of Cefepime in KPC-Producing Escherichia coli	PubMed	Other (outside the scope of this review)
220	Three-dimensional structure of AmpC beta-lactamase from Escherichia coli bound to a transition-state analogue: possible implications for the oxyanion hypothesis and for inhibitor design	PubMed	Structural or chemical-synthesis study
221	Predicting early appropriate therapy for patients infected by carbapenem-resistant Gram-negative pathogens in	PubMed	Other (outside the scope of this review)

	intensive care units in Italy		
222	Novel agents in development for multidrug-resistant Gram-negative infections: potential new options facing multiple challenges	PubMed	Other (outside the scope of this review)
223	Meropenem-Vaborbactam for the Treatment of Post-Neurosurgical Meningitis Caused by KPC Producer Klebsiella Pneumoniae: A Case Report and Review of the Literature	PubMed	Case report
224	Sulbactam-Durlobactam Plus Ceftriaxone Dosing and Novel Treatment Regimens for Mycobacterium abscessus Lung Disease	PubMed	Mycobacterium-focused
225	In vitro activity of sulbactam/durlobactam against clinical isolates of Acinetobacter baumannii collected in China	PubMed	In vitro activity or surveillance data
226	Pharmacokinetics of sulbactam-durlobactam in patients with Acinetobacter baumannii ventriculitis: a report of two cases	PubMed	PK/PD or pharmaceutical-technology study
227	Antimicrobial susceptibility of Enterobacterales causing infection in the elderly: focus on aztreonam-avibactam and recently approved β-lactamase inhibitor combinations	PubMed	Focused on other β-lactamase inhibitor combinations
228	Genome sequencing and molecular characterisation of XDR Acinetobacter baumannii reveal complexities in resistance: Novel combination of sulbactam-durlobactam holds promise for therapeutic intervention	PubMed	Other (outside the scope of this review)
229	Epidemiology of Meropenem/Vaborbactam Resistance in KPC-Producing Klebsiella pneumoniae Causing Bloodstream Infections in Northern Italy, 2018	PubMed	Epidemiology or outbreak report
230	Pharmacokinetics of Non-β-Lactam β-Lactamase Inhibitors	PubMed	PK/PD or pharmaceutical-technology study
231	In vitro activity of new combinations of β-lactam and β-lactamase inhibitors against the Mycobacterium tuberculosis complex	PubMed	Mycobacterium-focused
232	Plasmid-mediated AmpC beta-lactamase-producing Escherichia coli causing urinary tract infection in the Auckland community likely to be resistant to commonly prescribed antimicrobials	PubMed	Other (outside the scope of this review)
233	Bifunctional inhibitors of β-lactamases and ferroptosis: Design, synthesis and anti-gram-negative bacterial infection evaluation	PubMed	Structural or chemical-synthesis study
234	The Optimal Permeation of Cyclic Boronates to Cross the Outer Membrane via the Porin Pathway	PubMed	Other (outside the scope of this review)
235	Drug Discovery in the Field of β-	PubMed	General review or

	Lactams: An Academic Perspective		guidance (not boronate-focused)
236	Mechanism of action of cefiderocol	PubMed	Focused on cefiderocol or other novel agents
237	In Vitro Activity of Sulbactam-Durlobactam against Acinetobacter baumannii-calcoaceticus Complex Isolates Collected Globally in 2016 and 2017	PubMed	In vitro activity or surveillance data
238	Molecular characterization of a clinical ST145 Klebsiella oxytoca strain co-producing KPC-2 and IMP-96 carbapenemases	PubMed	Other (outside the scope of this review)
239	Emerging antibiotic resistance: carbapenemase-producing enterobacteria. Bad new bugs, still no new drugs	PubMed	Other (outside the scope of this review)
240	First identification and whole-genome characterization of human-associated Enterobacter roggenkampii and Enterobacter sichuanensis carrying bla(IMI-4) carbapenemase in Costa Rica	PubMed	Other (outside the scope of this review)
241	Characterizing the V516M penicillin-binding protein 2 (PBP2) mutation causing zidebactam resistance in Pseudomonas aeruginosa	PubMed	Focused on other β-lactamase inhibitor combinations
242	Synergistic Effect of Clinically Available Beta-Lactamase Inhibitors Combined with Cefiderocol against Carbapenemase-Producing Gram-Negative Organisms	PubMed	Focused on cefiderocol or other novel agents
243	Activity of imipenem/relebactam against KPC-producing Klebsiella pneumoniae and the possible role of Ompk36 mutation in determining resistance: an Italian retrospective analysis	PubMed	Focused on other β-lactamase inhibitor combinations
244	Activity of Aztreonam-avibactam and Ceftazidime-Avibactam against Enterobacterales and Pseudomonas aeruginosa causing infections in patients hospitalized in hematology, oncology, and transplant units from United States medical centres (2019-2024)	PubMed	Focused on other β-lactamase inhibitor combinations
245	Synergistic cefiderocol-containing antibiotic combinations active against highly drug-resistant Acinetobacter baumannii patient isolates with diverse resistance mechanisms	PubMed	Focused on cefiderocol or other novel agents
246	Emergence and Recovery of Ceftazidime-avibactam Resistance in blaKPC-33-Harboring Klebsiella pneumoniae Sequence Type 11 Isolates in China	PubMed	Other (outside the scope of this review)
247	Probing active site chemistry in SHV beta-lactamase variants at Ambler position 244. Understanding unique properties of inhibitor resistance	PubMed	Other (outside the scope of this review)

248	Activity of Meropenem Combined with RPX7009, a Novel β-Lactamase Inhibitor, against Gram-Negative Clinical Isolates in New York City	PubMed	Combination-therapy study
249	Inhibition of the class C beta-lactamase from Acinetobacter spp.: insights into effective inhibitor design	PubMed	Other (outside the scope of this review)
250	What are the most relevant publications in Clinical Microbiology in the last two years?	PubMed	Other (outside the scope of this review)
251	Characterization of resistance to newer antimicrobials among carbapenem-resistant Klebsiella pneumoniae in the post-acute-care setting	PubMed	Other (outside the scope of this review)
252	Occurrence of Beta-Lactamases in Colistin-Resistant Enterobacterales Strains in Poland - a Pilot Study	PubMed	Other (outside the scope of this review)
253	Inhibitor-based methods for the detection of KPC carbapenemase-producing Enterobacteriaceae in clinical practice by using boronic acid compounds	PubMed	Detection or diagnostic methodology
254	AmpC beta-lactamases in Klebsiella pneumoniae: An emerging threat to the paediatric patients	PubMed	Other (outside the scope of this review)
255	Functional analyses of AmpC beta-lactamase through differential stability	PubMed	PK/PD or pharmaceutical-technology study
256	Sulbactam-durlobactam: A Step Forward in Treating Carbapenem-Resistant Acinetobacter baumannii (CRAB) Infections	PubMed	Other (outside the scope of this review)
257	Sporadic occurrence of CMY-2-producing multidrug-resistant Escherichia coli of ST-complexes 38 and 448, and ST131 in Norway	PubMed	Other (outside the scope of this review)
258	Induced Heteroresistance in Carbapenem-Resistant Acinetobacter baumannii (CRAB) via Exposure to Human Pleural Fluid (HPF) and Its Impact on Cefiderocol Susceptibility	PubMed	Focused on cefiderocol or other novel agents
259	Activity of novel antibiotics against dual metallo-Beta-lactamase producing Enterobacter hormaechei clinical isolates	PubMed	In vitro activity or surveillance data
260	Game Changers: New β-Lactamase Inhibitor Combinations Targeting Antibiotic Resistance in Gram-Negative Bacteria	PubMed	Other (outside the scope of this review)
261	Sulbactam-Durlobactam in the Treatment of Multidrug-Resistant Acinetobacter baumannii: A Narrative Review	PubMed	General review or guidance (not boronate-focused)
262	Cefepime Combined with Late-Generation β-Lactamase Inhibitors: Mechanisms of Action, In Vitro Activity, PK/PD Characteristics, Clinical Evidence and Resistance Mechanisms	PubMed	Combination-therapy study
263	A 2018-2019 patent review of metallo beta-lactamase inhibitors	PubMed	General review or guidance (not

			boronate-focused)
264	Unraveling sulbactam-durlobactam: insights into its role in combating infections caused by Acinetobacter baumannii	PubMed	Other (outside the scope of this review)
265	Escherichia coli with penicillin-binding protein 3 insertions: implications for newer β-lactam/β-lactamase inhibitor combinations	PubMed	Other (outside the scope of this review)
266	Breaking Through Resistance: A Comparative Review of New Beta-Lactamase Inhibitors (Avibactam, Vaborbactam, Relebactam) Against Multidrug-Resistant Superbugs	PubMed	General review or guidance (not boronate-focused)
267	In Vitro Activity of New β-Lactamase Inhibitor Combinations against bla(NDM), bla(KPC), and ESBL-Producing Enterobacteriales Uropathogens	PubMed	In vitro activity or surveillance data
268	Carbapenemase Inhibitors: Updates on Developments in 2021	PubMed	General review or guidance (not boronate-focused)
269	Geographic differences in susceptibility profiles of potential non-class B carbapenemase-producing Enterobacterales isolates against ceftazidime-avibactam, meropenem-vaborbactam, colistin, amikacin, gentamicin, and tigecycline: Data from the Antimicrobial Testing Leadership and Surveillance, 2018-2022	PubMed	In vitro activity or surveillance data
270	Bronchopulmonary disposition of IV cefepime/taniborbactam (2-0.5 g) administered over 2 h in healthy adult subjects	PubMed	PK/PD or pharmaceutical-technology study
271	An Appraisal of the Pharmacokinetic and Pharmacodynamic Properties of Meropenem-Vaborbactam	PubMed	PK/PD or pharmaceutical-technology study
272	New β-Lactam/β-Lactamase Inhibitor Combination Antibiotics	PubMed	Other (outside the scope of this review)
273	Resistance to Ceftazidime/Avibactam, Meropenem/Vaborbactam and Imipenem/Relebactam in Gram-Negative MDR Bacilli: Molecular Mechanisms and Susceptibility Testing	PubMed	Focused on other β-lactamase inhibitor combinations
274	In vitro Synergistic and Bactericidal Effects of Aztreonam in Combination with Ceftazidime/ Avibactam, Meropenem/Vaborbactam and Imipenem/Relebactam Against Dual-Carbapenemase-Producing Enterobacterales	PubMed	Focused on other β-lactamase inhibitor combinations
275	Carbapenem-Resistant Enterobacterales: Considerations for Treatment in the Era of New Antimicrobials and Evolving Enzymology	PubMed	Other (outside the scope of this review)
276	Cefepime and New Cefepime/Beta-Lactamase Inhibitor Combination for the Treatment of Gram-Negative	PubMed	Structural or chemical-synthesis study

	Bacteria: Chemical Structure and Mechanism of Action, Microbiological Target, Clinical Use and PK/PD Characteristics		
277	Meropenem/Vaborbactam Plus Aztreonam as a Possible Treatment Strategy for Bloodstream Infections Caused by Ceftazidime/Avibactam-Resistant Klebsiella pneumoniae: A Retrospective Case Series and Literature Review	PubMed	Case report
278	Re: Potent in vitro activity of sulbactam-durlobactam against NDM-producing Escherichia coli including cefiderocol and aztreonam-avibactam-resistant isolates: author's response	PubMed	Correction, comment, or reply
279	Will morphing boron-based inhibitors beat the β-lactamases?	PubMed	Other (outside the scope of this review)
280	Acinetobacter baumannii OmpA Is a Selective Antibiotic Permeant Porin	PubMed	Other (outside the scope of this review)
281	Establishing the reference broth microdilution MIC method for cefepime-taniborbactam	PubMed	Other (outside the scope of this review)
282	Pharmacokinetics and Pharmacodynamics of β-Lactamase Inhibitors	PubMed	PK/PD or pharmaceutical-technology study
283	Erratum for Lickliter et al., "Safety, Pharmacokinetics, and Drug-Drug Interaction Potential of Intravenous Durlobactam, a β-Lactamase Inhibitor, in Healthy Subjects"	PubMed	Correction, comment, or reply
284	Clonal dissemination of Klebsiella pneumoniae resistant to cefiderocol, ceftazidime/avibactam, meropenem/vaborbactam and imipenem/relebactam co-producing KPC and OXA-181 carbapenemase	PubMed	Focused on cefiderocol or other novel agents
285	Effect of Colistin, Fosfomycin and Meropenem/Vaborbactam on Carbapenem-Resistant Enterobacterales in Egypt: A Cross-Sectional Study	PubMed	Other (outside the scope of this review)
286	Evaluation of the VITEK 2 AST-N439 card for susceptibility testing of novel β-lactam/β-lactamase inhibitor combinations and colistin in carbapenem-non-susceptible gram-negative bacilli	PubMed	In vitro activity or surveillance data
287	In vitro activity of cefiderocol in Pseudomonas aeruginosa isolates from people with cystic fibrosis recovered during three multicentre studies in Spain	PubMed	Focused on cefiderocol or other novel agents
288	Pharmacokinetic-Pharmacodynamic Target Attainment Analyses as Support for Meropenem-Vaborbactam Dosing Regimens and Susceptibility Breakpoints	PubMed	PK/PD or pharmaceutical-technology study
289	Novel Beta-Lactamase Inhibitors: Unlocking Their Potential in Therapy	PubMed	Other (outside the scope of this review)

290	First case report of a vertebral osteomyelitis caused by carbapenem-resistant Enterobacter cloacae treated with imipenem/cilastatin/relebactam prolonged infusion then meropenem/vaborbactam in continuous infusion	PubMed	Case report
291	Successful treatment of carbapenem-resistant Acinetobacter baumannii and Enterobacterales coinfection with sulbactam-durlobactam combined with aztreonam: a case report	PubMed	Case report
292	Innovative Antibiotic Therapies for Carbapenem-Resistant Gram-Negative Bacterial Infections: Clinical Efficacy, Safety, and Comparative Studies	PubMed	Other (outside the scope of this review)
293	Exploring β-lactam interactions with DacB1: unraveling optimal therapies for combating drug-resistant Mycobacterium tuberculosis	PubMed	Mycobacterium-focused
294	In-vitro activity of newly-developed β-lactamase inhibitors avibactam, relebactam and vaborbactam in combination with anti-pseudomonal β-lactam antibiotics against AmpC-overproducing clinical Pseudomonas aeruginosa isolates	PubMed	Combination-therapy study
295	Assessment of the activity and mechanisms of resistance to cefiderocol and combinations of β-lactams and the novel β-lactamase inhibitors avibactam, taniborbactam, zidebactam, nacubactam, xeruborbactam, and ANT3310 in emerging double-carbapenemase-producing Enterobacterales	PubMed	Focused on cefiderocol or other novel agents
296	In vitro and intracellular activity of vaborbactam combined with β-lactams against Mycobacterium abscessus	PubMed	Mycobacterium-focused
297	Non-KPC Attributes of Newer β-lactam/β-lactamase Inhibitors, Part 1: Enterobacterales and Pseudomonas aeruginosa	PubMed	Other (outside the scope of this review)
298	Activity of novel β-lactam/β-lactamase inhibitor combinations against serine carbapenemase-producing carbapenem-resistant Pseudomonas aeruginosa	PubMed	In vitro activity or surveillance data
299	Genomic analysis and antimicrobial activity of β-lactam/β-lactamase inhibitors and other agents against KPC-producing Klebsiella pneumoniae clinical isolates from Brazilian hospitals	PubMed	In vitro activity or surveillance data
300	Sulbactam/Durlobactam: First Approval	PubMed	Other (outside the scope of this review)
301	Epidemiology and In Vitro Activity of Ceftazidime/Avibactam, Meropenem/Vaborbactam and Imipenem/Relebactam against KPC-	PubMed	Focused on other β-lactamase inhibitor combinations

	Producing K. pneumoniae Collected from Bacteremic Patients, 2018 to 2020		
302	Stewardship Challenges, PK/PD Evidence, and Prescribing Appropriateness for Recently Approved Antibiotics in Pediatric Practice	PubMed	Other (outside the scope of this review)
303	Treatment of ventilator-associated pneumonia due to carbapenem-resistant Gram-negative bacteria with novel agents: a contemporary, multidisciplinary ESGCIP perspective	PubMed	General review or guidance (not boronate-focused)
304	Current Advances in Developing New Antimicrobial Agents Against Non-Tuberculous Mycobacterium	PubMed	Other (outside the scope of this review)
305	Role of a Real-Time TDM-Based Expert Clinical Pharmacological Advice Program in Optimizing the Early Pharmacokinetic/Pharmacodynamic Target Attainment of Continuous Infusion Beta-Lactams among Orthotopic Liver Transplant Recipients with Documented or Suspected Gram-Negative Infections	PubMed	PK/PD or pharmaceutical-technology study
306	Contemporary Perspective on the Treatment of Acinetobacter baumannii Infections: Insights from the Society of Infectious Diseases Pharmacists	PubMed	General review or guidance (not boronate-focused)
307	In vitro activity of aztreonam-nacubactam and cefepime-nacubactam against a global collection of clinically important Gram-negative Bacilli collected in 2021-2023	PubMed	Focused on other β-lactamase inhibitor combinations
308	Impact of porin deletions on cefepime-taniborbactam activity against Klebsiella pneumoniae	PubMed	In vitro activity or surveillance data
309	Resistance to Novel β-Lactam-β-Lactamase Inhibitor Combinations: The "Price of Progress"	PubMed	Other (outside the scope of this review)
310	Activity of Meropenem-Vaborbactam against Carbapenem-Resistant Enterobacteriaceae in a Murine Model of Pyelonephritis	PubMed	In vitro activity or surveillance data
311	New evidence in severe pneumonia: meropenem-vaborbactam	PubMed	Other (outside the scope of this review)
312	Emergence of KPC-8-producing K. pneumoniae infection without prior exposure to ceftazidime/avibactam: the threat of de novo infections by ceftazidime/avibactam-resistant KPC variants	PubMed	Resistance-mechanism or variant study
313	In vitro evaluation of meropenem-vaborbactam against clinical CRE isolates at a tertiary care center with low KPC-mediated carbapenem resistance	PubMed	Other (outside the scope of this review)
314	Meropenem/vaborbactam plus aztreonam for the treatment of New Delhi metallo-β-lactamase-producing	PubMed	Combination-therapy study

	Klebsiella pneumoniae infections		
315	A Ceftazidime-Avibactam-Resistant and Carbapenem-Susceptible Klebsiella pneumoniae Strain Harboring bla(KPC-14) Isolated in New York City	PubMed	Other (outside the scope of this review)
316	Meropenem/vaborbactam: a next generation β-lactam β-lactamase inhibitor combination	PubMed	Other (outside the scope of this review)
317	Pharmacokinetics of the Meropenem Component of Meropenem-Vaborbactam in the Treatment of KPC-Producing Klebsiella pneumoniae Bloodstream Infection in a Pediatric Patient	PubMed	PK/PD or pharmaceutical-technology study
318	Click Chemistry in Lead Optimization of Boronic Acids as β-Lactamase Inhibitors	PubMed	Other (outside the scope of this review)
319	Structure-based design of a potent transition state analogue for TEM-1 beta-lactamase	PubMed	Structural or chemical-synthesis study
320	Ceftibuten-Ledaborbactam Activity against Multidrug-Resistant and Extended-Spectrum-β-Lactamase-Positive Clinical Isolates of Enterobacterales from a 2018-2020 Global Surveillance Collection	PubMed	In vitro activity or surveillance data
321	Biochemical and structural analysis of inhibitors targeting the ADC-7 cephalosporinase of Acinetobacter baumannii	PubMed	Other (outside the scope of this review)
322	Targeting class A and C serine β-lactamases with a broad-spectrum boronic acid derivative	PubMed	Other (outside the scope of this review)
323	[The presence of extended spectrum beta-lactamase, KPC-type carbapenemase and plasmid-mediated AmpC beta-lactamase in E.coli and K.pneumoniae strains isolated from blood cultures]	PubMed	Other (outside the scope of this review)
324	Design and exploration of novel boronic acid inhibitors reveals important interactions with a clavulanic acid-resistant sulfhydryl-variable (SHV) β-lactamase	PubMed	Structural or chemical-synthesis study
325	Exploring sequence requirements for C_3/C_4 carboxylate recognition in the Pseudomonas aeruginosa cephalosporinase: Insights into plasticity of the AmpC β-lactamase	PubMed	Other (outside the scope of this review)
326	Nanomolar inhibitors of AmpC beta-lactamase	PubMed	Other (outside the scope of this review)
327	A sensitive and specific phenotypic assay for detection of metallo-β-lactamases and KPC in Klebsiella pneumoniae with the use of meropenem disks supplemented with aminophenylboronic acid, dipicolinic acid and cloxacillin	PubMed	Detection or diagnostic methodology
328	Identification of plasmid-mediated AmpC beta-lactamases in Escherichia	PubMed	In vitro activity or surveillance data

	coli, Klebsiella spp., and proteus species can potentially improve reporting of cephalosporin susceptibility testing results		
329	Wide dissemination of Gram-negative bacteria producing the taniborbactam-resistant NDM-9 variant: a One Health concern	PubMed	Epidemiology or outbreak report
330	The role of sulbactam-durlobactam in treating carbapenem-resistant Acinetobacter infections	PubMed	Other (outside the scope of this review)
331	Covalent docking of selected boron-based serine beta-lactamase inhibitors	PubMed	Structural or chemical-synthesis study
332	National Cohort of Compassionate Use of Meropenem-Vaborbactam: No Benefit over Meropenem for Pseudomonas aeruginosa	PubMed	Clinical-outcomes or pharmacoeconomic study
333	Comparative activity of newer β-lactam/β-lactamase inhibitor combinations against Pseudomonas aeruginosa from patients hospitalized with pneumonia in European medical centers in 2020	PubMed	In vitro activity or surveillance data
334	New perspectives in the antibiotic treatment of mechanically ventilated patients with infections from Gram-negatives	PubMed	General review or guidance (not boronate-focused)
335	Successful Treatment of Carbapenem-Resistant Acinetobacter baumannii Infection with Sulbactam-Durlobactam: A Two-Case Report	PubMed	Case report
336	Global trends in carbapenem-resistant gram-negative bacteria research (2020-2025): a bibliometric analysis and systematic review	PubMed	General review or guidance (not boronate-focused)
337	Effect of Ceftaroline, Ceftazidime/Avibactam, Ceftolozane/Tazobactam, and Meropenem/Vaborbactam on Establishment of Colonization by Vancomycin-Resistant Enterococci and Klebsiella pneumoniae in Mice	PubMed	Focused on other β-lactamase inhibitor combinations
338	Activity of Aztreonam/Avibactam and Recently Approved β-Lactamase Inhibitor Combinations against Enterobacterales and Pseudomonas aeruginosa from Intensive Care Unit and Non-Intensive Care Unit Patients	PubMed	Focused on other β-lactamase inhibitor combinations
339	The place of new antibiotics for Gram-negative bacterial infections in intensive care: report of a consensus conference	PubMed	Other (outside the scope of this review)
340	Choosing Optimal Antibiotics for the Treatment of Patients Infected With Enterobacteriaceae: A Network Meta-analysis and Cost-Effectiveness Analysis	PubMed	Clinical-outcomes or pharmacoeconomic study
341	Meropenem-Vaborbactam for Treatment of Carbapenem-Resistant Enterobacterales: A Narrative Review	PubMed	General review or guidance (not boronate-focused)

	of Clinical Practice Evidence		
342	Inhibitor Affinity Differs among Clinical Variants of IMP Metallo-β-Lactamases: Analysis and Implications for Inhibitor Design	PubMed	Other (outside the scope of this review)
343	[Carbapenem resistance in ESBL positive Enterobacteriaceae isolates causing invasive infections]	PubMed	Other (outside the scope of this review)
344	Carbapenem-Resistant Enterobacteriaceae Infections: Taiwan Aspects	PubMed	Other (outside the scope of this review)
345	Management of Severe Infections: Multidrug-Resistant and Carbapenem-Resistant Gram-Negative Bacteria	PubMed	Other (outside the scope of this review)
346	The role of new carbapenem combinations in the treatment of multidrug-resistant Gram-negative infections	PubMed	Other (outside the scope of this review)
347	Oral Antibiotics in Clinical Development for Community-Acquired Urinary Tract Infections	PubMed	Other (outside the scope of this review)
348	Investigational Agents for the Treatment of Gram-Negative Bacterial Infections: A Reality Check	PubMed	Other (outside the scope of this review)
349	VNRX-5133 (Taniborbactam), a Broad-Spectrum Inhibitor of Serine- and Metallo-β-Lactamases, Restores Activity of Cefepime in Enterobacterales and Pseudomonas aeruginosa	PubMed	In vitro activity or surveillance data
350	In Vitro Antibacterial Activity and In Vivo Efficacy of Sulbactam-Durlobactam against Pathogenic Burkholderia Species	PubMed	Other (outside the scope of this review)
351	Review of Ceftazidime-Avibactam, Meropenem-Vaborbactam, and Imipenem/Cilastatin-Relebactam to Target Klebsiella pneumoniae Carbapenemase-Producing Enterobacterales	PubMed	General review or guidance (not boronate-focused)
352	Investigating cefepime/taniborbactam for the treatment of complicated urinary tract infections	PubMed	Other (outside the scope of this review)
353	Activity of cefepime/taniborbactam and comparators against whole genome sequenced ertapenem-non-susceptible Enterobacterales clinical isolates: CANWARD 2007-19	PubMed	In vitro activity or surveillance data
354	Publisher Correction: Interplay between β-lactamases and new β-lactamase inhibitors	PubMed	Correction, comment, or reply
355	Cefepime-Taniborbactam-a Novel Combination Therapy for Multidrug-Resistant Pathogens	PubMed	Other (outside the scope of this review)
356	Treatment and diagnosis of severe KPC-producing Klebsiella pneumoniae infections: a perspective on what has changed over last decades	PubMed	General review or guidance (not boronate-focused)
357	Safety and pharmacokinetics of single and multiple doses of ledaborbactam	PubMed	PK/PD or pharmaceutical-

	etzadroxil with or without ceftibuten in healthy volunteers		technology study
358	Activity of mecillinam against AmpC beta-lactamase-producing Escherichia coli	PubMed	In vitro activity or surveillance data
359	Risk factors for in vivo emergence of ceftazidime-avibactam resistance in KPC-producing Klebsiella pneumoniae and the associated resistance mechanisms: a case-control study	PubMed	Other (outside the scope of this review)
360	Aztreonam/avibactam activity against Enterobacterales from European medical centres: summary of 5 years of surveillance prior to approval for clinical use (2019-2023)	PubMed	Focused on other β-lactamase inhibitor combinations
361	Activity of aztreonam-avibactam and ceftazidime-avibactam against β-lactamase-producing enterobacterales Isolates from United States hospitals	PubMed	Focused on other β-lactamase inhibitor combinations
362	The emerging concern of IMP variants being resistant to the only IMP-type metallo-β-lactamase inhibitor, xeruborbactam	PubMed	Other (outside the scope of this review)
363	Activity of ceftazidime/avibactam, meropenem/vaborbactam and imipenem/relebactam against carbapenemase-negative carbapenem-resistant Enterobacterales isolates from US hospitals	PubMed	Focused on other β-lactamase inhibitor combinations
364	Cost-effectiveness analysis of vaborem for the treatment of carbapenem-resistant Enterobacteriaceae-Klebsiella pneumoniae carbapenemase (CRE-KPC) infections in the UK	PubMed	Clinical-outcomes or pharmacoeconomic study
365	Increasing frequency of OXA-48-producing Enterobacterales worldwide and activity of ceftazidime/avibactam, meropenem/vaborbactam and comparators against these isolates	PubMed	In vitro activity or surveillance data
366	Structural Basis of Metallo-β-lactamase Inhibition by N-Sulfamoylpyrrole-2-carboxylates	PubMed	Other (outside the scope of this review)
367	Ceftazidime-avibactam, meropenen-vaborbactam, and imipenem-relebactam in combination with aztreonam against multidrug-resistant, metallo-β-lactamase-producing Klebsiella pneumoniae	PubMed	Focused on other β-lactamase inhibitor combinations
368	Mono vs. combo regimens with novel beta-lactam/beta-lactamase inhibitor combinations for the treatment of infections due to carbapenemase-producing Enterobacterales: insights from the literature	PubMed	Other (outside the scope of this review)
369	Aztreonam Combinations with Avibactam, Relebactam, and Vaborbactam as Treatment for New Delhi Metallo-β-Lactamase-Producing Enterobacterales Infections-In Vitro Susceptibility Testing	PubMed	In vitro activity or surveillance data

370	Treatment of infections caused by multidrug-resistant Gram-negative bacilli: A practical approach by the Italian (SIMIT) and French (SPILF) Societies of Infectious Diseases	PubMed	Other (outside the scope of this review)
371	In Vitro Activity of Two Cefepime-Based Novel Combinations, Cefepime/Taniborbactam and Cefepime/Zidebactam, against Carbapenemase-Expressing Enterobacterales Collected in India	PubMed	Focused on other β-lactamase inhibitor combinations
372	[New β-lactam antibiotics and β-lactamase inhibitors against multidrug-resistant Gram-negative bacteria]	PubMed	Other (outside the scope of this review)
373	Molecular drivers of resistance to sulbactam-durlobactam in contemporary clinical isolates of Acinetobacter baumannii	PubMed	Other (outside the scope of this review)
374	Antimicrobial and Diagnostic Stewardship of the Novel β-Lactam/β-Lactamase Inhibitors for Infections Due to Carbapenem-Resistant Enterobacterales Species and Pseudomonas aeruginosa	PubMed	Other (outside the scope of this review)
375	In vitro activity of Biapenem plus RPX7009, a carbapenem combined with a serine β-lactamase inhibitor, against anaerobic bacteria	PubMed	Combination-therapy study
376	Antibiotic therapy for nonfermenting Gram-negative bacilli infections: future perspectives	PubMed	General review or guidance (not boronate-focused)
377	Frequency, Antimicrobial Susceptibility, and Molecular Characterization of Carbapenem-Resistant Enterobacterales Stratified by United States Census Divisions: Results From the INFORM Program (2018-2022)	PubMed	In vitro activity or surveillance data
378	In Vitro Activity of Novel β-Lactam/β-Lactamase Inhibitors Against Carbapenem-Resistant Pseudomonas aeruginosa and Enterobacterales in Korea	PubMed	In vitro activity or surveillance data
379	β-Lactam/β-Lactamase Inhibitor Combinations in Sepsis-Associated Acute Kidney Injury and Renal Replacement Therapy	PubMed	Other (outside the scope of this review)
380	Clinical Evaluation of Meropenem-Vaborbactam Combination for the Treatment of Urinary Tract Infection: Evidence to Date	PubMed	Other (outside the scope of this review)
381	Potentially effective antimicrobial treatment for pneumonia caused by isolates of carbapenem-resistant and extensively drug-resistant Acinetobacter baumannii complex species: what can we expect in the future?	PubMed	Other (outside the scope of this review)
382	Reviving β-lactams for Mycobacterium	PubMed	Mycobacterium-

	abscessus: the emerging role of durlobactam		focused
383	The antibiotic pipeline for multi-drug resistant gram negative bacteria: what can we expect?	PubMed	General review or guidance (not boronate-focused)
384	Overview of novel cefepime-based β-lactam/β-lactamase inhibitor combinations	PubMed	General review or guidance (not boronate-focused)
385	Pharmacokinetics, safety and population pharmacokinetics/pharmacodynamics analysis of meropenem-vaborbactam in Chinese healthy participants	PubMed	PK/PD or pharmaceutical-technology study
386	In Vitro Activity of the Ultrabroad-Spectrum Beta-Lactamase Inhibitor QPX7728 in Combination with Multiple Beta-Lactam Antibiotics against Pseudomonas aeruginosa	PubMed	Combination-therapy study
387	Interactions of the Diazabicyclooctane Serine β-Lactamase Inhibitor ETX1317 with Target Enzymes	PubMed	Other (outside the scope of this review)
388	Structural Insights into Inhibition of the Acinetobacter-Derived Cephalosporinase ADC-7 by Ceftazidime and Its Boronic Acid Transition State Analog	PubMed	Other (outside the scope of this review)
389	Oral cephalosporin and β-lactamase inhibitor combinations for ESBL-producing Enterobacteriaceae urinary tract infections	PubMed	Other (outside the scope of this review)
390	Meropenem-RPX7009 Concentrations in Plasma, Epithelial Lining Fluid, and Alveolar Macrophages of Healthy Adult Subjects	PubMed	PK/PD or pharmaceutical-technology study
391	A reliable phenotypic assay for detection of ESBLs and AmpCs in MBL-producing gram-negative bacteria with the use of aminophenylboronic acid, dipicolinic acid and cloxacillin	PubMed	Detection or diagnostic methodology
392	Activity of Aztreonam-avibactam and other β-lactamase inhibitor combinations against Gram-negative bacteria isolated from patients hospitalized with pneumonia in United States medical centers (2020-2022)	PubMed	Focused on other β-lactamase inhibitor combinations
393	In vivo dose response and efficacy of the β-lactamase inhibitor, durlobactam, in combination with sulbactam against the Acinetobacter baumannii-calcoaceticus complex	PubMed	PK/PD or pharmaceutical-technology study
394	Reduced susceptibility to aztreonam-avibactam conferred by acquired AmpC-type β-lactamases in PBP3-modified Escherichia coli	PubMed	Focused on other β-lactamase inhibitor combinations
395	Activity of meropenem/vaborbactam and comparators against non-carbapenemase-producing carbapenem-resistant Enterobacterales isolates from Europe	PubMed	In vitro activity or surveillance data
396	In vitro efficacy of	PubMed	Mycobacterium-

	sulbactam/durlobactam combined with β-lactam antibiotics in Australian Mycobacterium abscessus isolates		focused
397	Complete genome sequences of KPC-producing Klebsiella pneumoniae isolates pre- and post-development of meropenem/vaborbactam resistance	PubMed	Other (outside the scope of this review)
398	The Role of Colistin in the Era of New β-Lactam/β-Lactamase Inhibitor Combinations	PubMed	Other (outside the scope of this review)
399	Cefiderocol and Sulbactam-Durlobactam against Carbapenem-Resistant Acinetobacter baumannii	PubMed	Focused on cefiderocol or other novel agents
400	Plasma and intrapulmonary pharmacokinetics of ceftibuten and ledaborbactam in healthy male and female adults	PubMed	PK/PD or pharmaceutical-technology study
401	ARGONAUT-IV: susceptibility of carbapenemase-producing Klebsiella pneumoniae to the oral bicyclic boronate β-lactamase inhibitor ledaborbactam combined with ceftibuten	PubMed	Combination-therapy study
402	Relative inhibitory activities of newly developed diazabicyclooctanes, boronic acid derivatives, and penicillin-based sulfone β-lactamase inhibitors against broad-spectrum AmpC β-lactamases	PubMed	Other (outside the scope of this review)
403	Meropenem-Vaborbactam as Salvage Therapy for Ceftazidime-Avibactam-, Cefiderocol-Resistant ST-512 Klebsiella pneumoniae-Producing KPC-31, a D179Y Variant of KPC-3	PubMed	Focused on cefiderocol or other novel agents
404	Sulbactam-Durlobactam: A Novel Antibiotic Combination for the Treatment of Acinetobacter baumannii-Calcoaceticus Complex (ABC) Hospital-Acquired Bacterial Pneumonia and Ventilator-Associated Bacterial Pneumonia	PubMed	Other (outside the scope of this review)
405	Efficacy and safety of novel carbapenem-β-lactamase inhibitor combinations: Results from phase II and III trials	PubMed	Other (outside the scope of this review)
406	Revitalizing cephalosporins: The promise of β-lactamase inhibitor combinations	PubMed	Other (outside the scope of this review)
407	Targeting KPC-Producing Klebsiella pneumoniae Biofilms: Genomic Profiles, Clinical Outcomes and Novel Combinations	PubMed	Clinical-outcomes or pharmacoeconomic study
408	New Drugs for the Treatment of Pseudomonas aeruginosa Infections with Limited Treatment Options: A Narrative Review	PubMed	General review or guidance (not boronate-focused)
409	Meropenem-ANT3310, a unique β-lactam-β-lactamase inhibitor combination with expanded antibacterial spectrum against Gram-	PubMed	Focused on other β-lactamase inhibitor combinations

	negative pathogens including carbapenem-resistant Acinetobacter baumannii		
410	Reviewing novel treatment options for carbapenem-resistant Enterobacterales	PubMed	General review or guidance (not boronate-focused)
411	Multidrug-resistant Gram-negative clinical isolates with reduced susceptibility/resistance to cefiderocol: which are the best present and future therapeutic alternatives?	PubMed	Focused on cefiderocol or other novel agents
412	Impact of acquired broad-spectrum β-lactamases on susceptibility to oral penems/carbapenems (tebipenem, sulopenem, and faropenem) alone or in combination with avibactam and taniborbactam β-lactamase inhibitors in Escherichia coli	PubMed	Combination-therapy study
413	In vitro activity of sulbactam-durlobactam against colistin-resistant and/or cefiderocol-non-susceptible, carbapenem-resistant Acinetobacter baumannii collected in U.S. hospitals	PubMed	Focused on cefiderocol or other novel agents
414	Case Report: Successful salvage therapy with sulbactam-durlobactam combined with meropenem for carbapenem-resistant Acinetobacter baumannii bloodstream infection in a child after failure of allogeneic hematopoietic stem cell transplantation	PubMed	Case report
415	Efficacy of ceftazidime-avibactam, meropenem-vaborbactam, and imipenem-relebactam combinations against carbapenemase-producing Enterobacterales in Switzerland	PubMed	Focused on other β-lactamase inhibitor combinations
416	Impact of Acquired Broad Spectrum β-Lactamases on Susceptibility to Novel Combinations Made of β-Lactams (Aztreonam, Cefepime, Meropenem, and Imipenem) and Novel β-Lactamase Inhibitors in Escherichia coli and Pseudomonas aeruginosa	PubMed	In vitro activity or surveillance data
417	Therapeutic challenges and emerging strategies against carbapenem-resistant Acinetobacter baumannii	PubMed	Other (outside the scope of this review)
418	The Effect of Double Carbapenem Regimen in the Management of Carbapenem-Resistant Klebsiella pneumoniae Infections: A Report of Five Cases	PubMed	Other (outside the scope of this review)
419	In vitro activity of newer β-lactam/β-lactamase inhibitor combinations, cefiderocol, plazomicin and comparators against carbapenemase-producing Klebsiella pneumoniae isolates	PubMed	Focused on cefiderocol or other novel agents
420	[New antibacterial agents on the market and in the pipeline]	PubMed	General review or guidance (not boronate-focused)

421	Treatment of UTIs Due to Klebsiella pneumoniae Carbapenemase-Producers: How to Use New Antibiotic Drugs? A Narrative Review	PubMed	General review or guidance (not boronate-focused)
422	Triazole-substituted phenylboronic acids as tunable lead inhibitors of KPC-2 antibiotic resistance	PubMed	Other (outside the scope of this review)
423	Non-β-lactam inhibitors targeting KPC-2 β-lactamase: Structural insights and rational design strategies to combat antimicrobial drug resistance	PubMed	Other (outside the scope of this review)
424	Synergistic Meropenem/Vaborbactam Plus Fosfomycin Treatment of KPC Producing K. pneumoniae Septic Thrombosis Unresponsive to Ceftazidime/Avibactam: From the Bench to the Bedside	PubMed	Combination-therapy study
425	New treatment options for multiresistant gram negatives	PubMed	Other (outside the scope of this review)
426	Multidrug-Resistant Acinetobacter baumannii: Resistance Mechanisms, Emerging Therapies, and Prevention-A Narrative Review	PubMed	General review or guidance (not boronate-focused)
427	Innovative β-lactam/β-lactamase inhibitor combinations for carbapenem-resistant Gram-negative bacteria	PubMed	Other (outside the scope of this review)
428	Management of MDR/XDR severe infections in the critically ill	PubMed	Other (outside the scope of this review)
429	Use of Newer and Repurposed Antibiotics against Gram-Negative Bacteria in Neonates	PubMed	Other (outside the scope of this review)
430	Comparative in vitro efficacy of aztreonam-avibactam and other alternatives (cefepime-taniborbactam, cefepime-zidebactam, and cefiderocol) against metallo-β-lactamase-producing Enterobacterales isolated in 2024 in France	PubMed	Focused on cefiderocol or other novel agents
431	Phosphonic Acid-Boronic Acid Anhydrides Demonstrate Class A-D β-Lactamase Inhibition	PubMed	Other (outside the scope of this review)
432	NDM-63: a novel NDM metallo-β-lactamase variant in the L3 loop, from a Klebsiella pneumoniae clinical isolate	PubMed	Other (outside the scope of this review)
433	In vitro susceptibility of cefepime-enmetazobactam, cefepime-taniborbactam and cefepime-zidebactam towards carbapenemase-producing Enterobacterales	PubMed	Focused on other β-lactamase inhibitor combinations
434	The Role of β-Lactam Antibiotics in Treating Mycobacterium abscessus: From Laboratory Insights to Clinical Applications and the Case for Clinical Trials	PubMed	Mycobacterium-focused
435	In Vitro Activity of Imipenem-Relebactam, Meropenem-Vaborbactam, Ceftazidime-Avibactam and Comparators on Carbapenem-Resistant Non-Carbapenemase-Producing	PubMed	Focused on other β-lactamase inhibitor combinations

	Enterobacterales		
436	β-lactam/β-lactamase inhibitor combinations: an update	PubMed	General review or guidance (not boronate-focused)
437	Pharmacokinetics and Tolerability of Intravenous Sulbactam-Durlobactam with Imipenem-Cilastatin in Hospitalized Adults with Complicated Urinary Tract Infections, Including Acute Pyelonephritis	PubMed	PK/PD or pharmaceutical-technology study
438	Assessment of human exposures of cefepime-taniborbactam against cefepime-resistant Enterobacterales and Pseudomonas aeruginosa in a 7-day hollow fiber infection model	PubMed	Other (outside the scope of this review)
439	Allergic Reactions and Cross-Reactivity Potential with Beta-Lactamase Inhibitors	PubMed	Other (outside the scope of this review)
440	Design and SAR Analysis of Phenylboronic Acid-Based Inhibitors for Sensitizing KPC-2-Producing Klebsiella pneumoniae to β-Lactam Antibiotics	PubMed	Structural or chemical-synthesis study
441	In vitro activity of cefiderocol against European Enterobacterales, including isolates resistant to meropenem and recentβ-lactam/β-lactamase inhibitor combinations	PubMed	Focused on cefiderocol or other novel agents
442	Clinical efficacy, safety and pharmacokinetics of novel β-lactam/β-lactamase inhibitor combinations: a systematic review	PubMed	PK/PD or pharmaceutical-technology study
443	Sulbactam-durlobactam improves cephalosporin and carbapenem susceptibility and time-kill effect against Mycobacterium abscessus	PubMed	Mycobacterium-focused
444	Pathogen-Targeted Clinical Development to Address Unmet Medical Need: Design, Safety, and Efficacy of the ATTACK Trial	PubMed	Other (outside the scope of this review)
445	Antimicrobial activity of aztreonam/avibactam and molecular characterization of Enterobacterales not susceptible to ceftazidime/avibactam and/or meropenem/vaborbactam from US medical centres (2016-2024)	PubMed	Focused on other β-lactamase inhibitor combinations
446	Real-World Evidence and Multidrug Resistant Infections: How Can We Leverage RWE to Improve Patient Outcome with the Novel Beta-Lactam and Beta-Lactam/Beta-Lactamase Inhibitor Combinations	PubMed	Clinical-outcomes or pharmacoeconomic study
447	Disarming carbapenemase-producing Acinetobacter baumannii: high potency of the novel therapeutic combination of meropenem and the innovative diazabicyclooctane β-lactamase inhibitor pilabactam (formerly ANT3310)	PubMed	Focused on other β-lactamase inhibitor combinations

448	Comparative activity of established versus new-generation β-lactams against AmpC-hyperproducing clinical isolates of Enterobacter cloacae complex and Klebsiella aerogenes: a multicentre study	PubMed	In vitro activity or surveillance data
449	Effectiveness and safety of meropenem-vaborbactam versus ceftazidime-avibactam in multidrug-resistant Gram-negative infections: a systematic review and meta-analysis with trial sequential analysis	PubMed	General review or guidance (not boronate-focused)
450	The epidemiological and molecular characteristics and sulbactam-durlobactam susceptibility of carbapenem-resistant Acinetobacter baumannii in a Chinese teaching hospital	PubMed	In vitro activity or surveillance data
451	Tricyclic boronic acids as broad-spectrum serine and metallo-β-lactamase inhibitors with in vitro activity against Acinetobacter baumannii: a patent evaluation (US 2025/0223303)	PubMed	Patent
452	A microbiological and structural analysis of the interplay between sulbactam/durlobactam and imipenem against penicillin-binding proteins (PBPs) of Acinetobacter spp	PubMed	Other (outside the scope of this review)
453	Antimicrobial resistance among imipenem-non-susceptible Escherichia coli and Klebsiella pneumoniae isolates, with an emphasis on novel β-lactam/β-lactamase inhibitor combinations and tetracycline derivatives: The Taiwan surveillance of antimicrobial resistance program, 2020-2022	PubMed	In vitro activity or surveillance data
454	Restoring ceftolozane susceptibility: a role for diazabicyclooctane β-lactamase inhibitors?	PubMed	Focused on other β-lactamase inhibitor combinations
455	In vitro antibacterial activity of sulbactam-durlobactam in combination with other antimicrobial agents against Acinetobacter baumannii-calcoaceticus complex	PubMed	Combination-therapy study
456	Susceptibility evaluation of novel beta-lactam/beta-lactamase inhibitor combinations against carbapenem-resistant Klebsiella pneumoniae from bloodstream infections in hospitalized patients in Brazil	PubMed	In vitro activity or surveillance data
457	Activity of cefepime, carbapenems and new β-lactam/β-lactamase inhibitor combinations on Enterobacter cloacae complex and Klebsiella aerogenes in Spain (SMART 2016-2022)	PubMed	In vitro activity or surveillance data
458	An update on cefepime and its future role in combination with novel β-lactamase inhibitors for MDR	PubMed	Combination-therapy study

	Enterobacterales and Pseudomonas aeruginosa		
459	Ceftazidime-Avibactam Resistance Mutations V240G, D179Y, and D179Y/T243M in KPC-3 β-Lactamase Do Not Alter Cefpodoxime-ETX1317 Susceptibility	PubMed	In vitro activity or surveillance data
460	Potent inhibitory activity of taniborbactam towards NDM-1 and NDM-1(Q119X) mutants, and in vitro activity of cefepime/taniborbactam against MBLs producing Enterobacterales	PubMed	In vitro activity or surveillance data
461	In vitro Activity of Meropenem-Vaborbactam versus Other Antibiotics Against Carbapenem-Resistant Escherichia coli from Southeastern China	PubMed	In vitro activity or surveillance data
462	Meropenem/Vaborbactam and Cefiderocol as Combination or Monotherapy to Treat Multi-Drug Resistant Gram-Negative Infections: A Regional Cross-Sectional Survey from Piedmont Infectious Disease Unit Network (PIDUN)	PubMed	Focused on cefiderocol or other novel agents
463	Treatment of severe infections due to metallo-β-lactamases-producing Gram-negative bacteria	PubMed	Other (outside the scope of this review)
464	Rationale and evidence for the use of new beta-lactam/beta-lactamase inhibitor combinations and cefiderocol in critically ill patients	PubMed	Focused on cefiderocol or other novel agents
465	In Vitro Activity of the Ultra-Broad-Spectrum Beta-Lactamase Inhibitor QPX7728 in Combination with Meropenem against Clinical Isolates of Carbapenem-Resistant Acinetobacter baumannii	PubMed	Combination-therapy study
466	In Vivo Activity of QPX7728, an Ultrabroad-Spectrum Beta-Lactamase Inhibitor, in Combination with Beta-Lactams against Carbapenem-Resistant Klebsiella pneumoniae	PubMed	Combination-therapy study
467	Assessment of Meropenem and Vaborbactam Room Temperature and Refrigerated Stability in Polyvinyl Chloride Bags and Elastomeric Devices	PubMed	PK/PD or pharmaceutical-technology study
468	Multicenter Clinical Evaluation of Etest Meropenem-Vaborbactam (bioMérieux) for Susceptibility Testing of Enterobacterales (Enterobacteriaceae) and Pseudomonas aeruginosa	PubMed	In vitro activity or surveillance data
469	β-Lactamases and β-Lactamase Inhibitors in the 21st Century	PubMed	Other (outside the scope of this review)
470	Meropenem/Vaborbactam: A Review in Complicated Urinary Tract Infections	PubMed	General review or guidance (not boronate-focused)
471	Meropenem/Vaborbactam, the First Carbapenem/β-Lactamase Inhibitor	PubMed	Other (outside the scope of this review)

	Combination		
472	Physical Compatibility of Meropenem and Vaborbactam With Select Intravenous Drugs During Simulated Y-site Administration	PubMed	PK/PD or pharmaceutical-technology study
473	In Vitro Antimicrobial Activity of Five Newly Approved Antibiotics against Carbapenemase-Producing Enterobacteria-A Pilot Study in Bulgaria	PubMed	In vitro activity or surveillance data
474	Detection of KPC Carbapenemase in Pseudomonas aeruginosa Isolated From Clinical Samples Using Modified Hodge Test and Boronic Acid Phenotypic Methods and Their Comparison With the Polymerase Chain Reaction	PubMed	Detection or diagnostic methodology
475	[New Antibiotics for Treatment of Highly Resistant Gram-negative Bacteria]	PubMed	Other (outside the scope of this review)
476	The β-Lactamase Inhibitor Boronic Acid Derivative SM23 as a New Anti-Pseudomonas aeruginosa Biofilm	PubMed	Other (outside the scope of this review)
477	Fast and Sensitive Method for Simultaneous Quantification of Meropenem and Vaborbactam in Human Plasma Microsamples by Liquid Chromatography-Tandem Mass Spectrometry for Therapeutic Drug Monitoring	PubMed	PK/PD or pharmaceutical-technology study
478	Current Positioning against Severe Infections Due to Klebsiella pneumoniae in Hospitalized Adults	PubMed	Other (outside the scope of this review)
479	Review of novel β-lactams and β-lactam/β-lactamase inhibitor combinations with implications for pediatric use	PubMed	General review or guidance (not boronate-focused)
480	Comparative in vitro analysis of sulbactam-durlobactam against extensively drug resistant (XDR) and non-XDR carbapenem-resistant Acinetobacter baumannii (CRAB) clinical isolates collected from a tertiary care facility in North India	PubMed	Other (outside the scope of this review)
481	Pharmacokinetic/pharmacodynamic target attainment in critically ill renal patients on antimicrobial usage: focus on novel beta-lactams and beta lactams/beta-lactamase inhibitors	PubMed	PK/PD or pharmaceutical-technology study
482	Place in Therapy of the Newly Available Armamentarium for Multi-Drug-Resistant Gram-Negative Pathogens: Proposal of a Prescription Algorithm	PubMed	General review or guidance (not boronate-focused)
483	X-ray Crystallography Deciphers the Activity of Broad-Spectrum Boronic Acid β-Lactamase Inhibitors	PubMed	In vitro activity or surveillance data
484	Therapeutic drug monitoring (TDM) of β-lactam/β-lactamase inhibitor (BL/BLI) drug combinations: insights from a pharmacometric simulation	PubMed	PK/PD or pharmaceutical-technology study

	study		
485	Klebsiella pneumoniae carbapenemase variants: the new threat to global public health	PubMed	Other (outside the scope of this review)
486	What's new in the treatment of multidrug-resistant gram-negative infections?	PubMed	Other (outside the scope of this review)
487	Boronate-Based Inhibitors of Penicillin-Binding Proteins: An Underestimated Avenue for Antibiotic Discovery?	PubMed	Other (outside the scope of this review)
488	Plazomicin for the treatment of multidrug-resistant Klebsiella bacteraemia in a patient with underlying chronic kidney disease and acute renal failure requiring renal replacement therapy	PubMed	Other (outside the scope of this review)
489	Aztreonam/avibactam activity against a large collection of carbapenem-resistant Enterobacterales (CRE) collected in hospitals from Europe, Asia and Latin America (2019-21)	PubMed	Focused on other β-lactamase inhibitor combinations
490	Two Comprehensive Liquid Chromatography High-Resolution Mass Spectrometry (UPLC-MS/MS) Multi-Methods for Real-Time Therapeutic Drug Monitoring (TDM) of Five Novel Beta-Lactams and of Fosfomycin Administered by Continuous Infusion	PubMed	PK/PD or pharmaceutical-technology study
491	Carbapenem Resistance in Acinetobacter baumannii: Mechanisms, Therapeutics, and Innovations	PubMed	Other (outside the scope of this review)
492	Treatment options for multidrug-resistant Gram-negatives in urinary tract infections	PubMed	Other (outside the scope of this review)
493	Evaluating the clinical effectiveness of new beta-lactam/beta-lactamase inhibitor combination antibiotics: A systematic literature review and meta-analysis	PubMed	General review or guidance (not boronate-focused)
494	Update on the comparative in vitro activity of cefiderocol and four β-lactam-β-lactamase-inhibitor combinations against clinically important Gram-negative pathogens	PubMed	Focused on cefiderocol or other novel agents
495	Antimicrobial Activity of Aztreonam in Combination with Old and New β-Lactamase Inhibitors against MBL and ESBL Co-Producing Gram-Negative Clinical Isolates: Possible Options for the Treatment of Complicated Infections	PubMed	Combination-therapy study
496	Utility of a TDM-Guided Expert Clinical Pharmacological Advice Program for Optimizing the Use of Novel Beta-Lactam/Beta-Lactamase Inhibitor Combinations and Cefiderocol in a Tertiary University Hospital: An Interim Analysis	PubMed	Focused on cefiderocol or other novel agents
497	New Antibiotics for the Treatment of	PubMed	Other (outside the

	Nosocomial Central Nervous System Infections		scope of this review)
498	Simultaneous post-neurosurgical ventriculitis and bacteraemia by two different strains of KPC-producing K. pneumoniae successfully treated with meropenem/vaborbactam and high dose of fosfomycin	PubMed	Other (outside the scope of this review)
499	Efficacy and In Vitro Activity of Novel Antibiotics for Infections With Carbapenem-Resistant Gram-Negative Pathogens	PubMed	In vitro activity or surveillance data
500	Newer antibiotics for drug-resistant Gram-negative infections in immunocompromised hosts: from pivotal trials to high-risk practice	PubMed	Other (outside the scope of this review)
501	Treatment of multidrug-resistant Gram-negative skin and soft tissue infections	PubMed	Other (outside the scope of this review)
502	Identification of two multidrug-resistant Pseudomonas aeruginosa clonal lineages with a countrywide distribution in Hungary	PubMed	Other (outside the scope of this review)
503	Pathogens constancy, harbinger of nosocomial infection cum identification of resistant genes and drug designing	PubMed	Other (outside the scope of this review)
504	A comparative analysis of performance and time-to-result for VITEK REVEAL versus MicroScan during antimicrobial susceptibility testing for gram-negative bacteremia	PubMed	In vitro activity or surveillance data
505	Treatment of Infections Caused by Extended-Spectrum-Beta-Lactamase-, AmpC-, and Carbapenemase-Producing Enterobacteriaceae	PubMed	Other (outside the scope of this review)
506	Continuous infusion of meropenem-vaborbactam for a KPC-3-producing Klebsiella pneumoniae bloodstream infection in a critically ill patient with augmented renal clearance	PubMed	Other (outside the scope of this review)
507	In vitro activity of imipenem/relebactam, meropenem/vaborbactam and comparators against Pseudomonas aeruginosa in Taiwan: Results from the Study for Monitoring Antimicrobial Resistance Trends (SMART) in 2020	PubMed	Focused on other β-lactamase inhibitor combinations
508	Effect of detergent on "promiscuous" inhibitors	PubMed	Other (outside the scope of this review)
509	In vitro activity of ceftazidime-avibactam, imipenem-cilastatin-relebactam, meropenem-vaborbactam, cefiderocol, and plazomicin against non-fermenting Gram-negative isolates obtained from Bulgarian inpatients	PubMed	Focused on cefiderocol or other novel agents
510	Beta-lactamase-catalyzed aminolysis of depsipeptides: peptide inhibition and a new kinetic mechanism	PubMed	Other (outside the scope of this review)
511	Emergence of KPC-producing Pseudomonas aeruginosa in Spain:	PubMed	Focused on other β-lactamase inhibitor

	insights into an outbreak and resistance to the novel carbapenem/β-lactamase inhibitor combinations imipenem/relebactam and meropenem/vaborbactam		combinations
512	Targeting carbapenem-resistant and hypervirulent Klebsiella pneumoniae: in vitro evaluation of cefepime/zidebactam, aztreonam/avibactam, imipenem/relebactam, and meropenem/vaborbactam	PubMed	Focused on other β-lactamase inhibitor combinations
513	New β-Lactam-β-Lactamase Inhibitor Combinations	PubMed	Other (outside the scope of this review)
514	Impact of loading dose β-lactam therapy on outcomes of KPC-producing Klebsiella pneumoniae bloodstream infections in non-ICU patients: a real world study	PubMed	Clinical-outcomes or pharmacoeconomic study
515	Safety and Pharmacokinetics in Human Volunteers of Taniborbactam (VNRX-5133), a Novel Intravenous β-Lactamase Inhibitor	PubMed	PK/PD or pharmaceutical-technology study
516	In Vitro Antimicrobial Activities of Carbapenem-beta Lactamase Inhibitor Combinations Against Carbapenem-resistant Gram-negative Pathogens Isolated from Türkiye	PubMed	Other (outside the scope of this review)
517	Selection of QPX7831, an Orally Bioavailable Prodrug of Boronic Acid β-Lactamase Inhibitor QPX7728	PubMed	Other (outside the scope of this review)
518	Susceptibilities of cefiderocol, meropenem-xeruborbactam, cefepime-taniborbactam, aztreonam-avibactam, and sulbactam-durlobactam against imipenem-non-susceptible Gram-negative bacilli in Taiwan	PubMed	Focused on cefiderocol or other novel agents
519	The Triple Combination of Meropenem, Avibactam, and a Metallo-β-Lactamase Inhibitor Optimizes Antibacterial Coverage Against Different β-Lactamase Producers	PubMed	Other (outside the scope of this review)
520	Unlocking potentials: the impact of meropenem, meropenem-vaborbactam, and ceftazidime-avibactam in combating carbapenem-resistant Enterobacter cloacae	PubMed	Other (outside the scope of this review)
521	Approachable Synthetic Methodologies for Second-Generation β-Lactamase Inhibitors: A Review	PubMed	General review or guidance (not boronate-focused)
522	Beyond structure and activity: targeting class A carbapenemases with monocyclic and bicyclic boronic acids to counter antimicrobial resistance	PubMed	Structural or chemical-synthesis study
523	Microbiology of Meropenem-Vaborbactam: A Novel Carbapenem Beta-Lactamase Inhibitor Combination for Carbapenem-Resistant Enterobacterales Infections	PubMed	Other (outside the scope of this review)
524	Classification and applicability of new	PubMed	Other (outside the

	beta-lactamase inhibitors		scope of this review)
525	Overview of meropenem-vaborbactam and newer antimicrobial agents for the treatment of carbapenem-resistant Enterobacteriaceae	PubMed	General review or guidance (not boronate-focused)
526	Contribution of mutational resistance mechanisms and acquired β-lactamases to cefiderocol/xeruborbactam susceptibility in Pseudomonas aeruginosa	PubMed	Focused on cefiderocol or other novel agents
527	Durlobactam in combination with β-lactams to combat Mycobacterium abscessus	PubMed	Mycobacterium-focused
528	Structural Optimization of Bicyclic Oxo-Boronates as Dual Metallo- and Serine-β-Lactamase Inhibitors	PubMed	Other (outside the scope of this review)
529	Precision Approaches to Carbapenem-Resistant Infections in the ICU: Integrating Diagnostics, Stewardship, and Novel Therapies	PubMed	Other (outside the scope of this review)
530	Efficacy of cefiderocol in combination with xeruborbactam versus taniborbactam against cefiderocol-resistant NDM-producing Pseudomonas aeruginosa	PubMed	Focused on cefiderocol or other novel agents
531	Managing KPC infections in daily practice: when BLBLIs are interchangeable - and when they are not	PubMed	Other (outside the scope of this review)
532	Molecular and pharmacodynamic insights into β-lactam therapy for high-inoculum Enterobacter cloacae complex infections	PubMed	PK/PD or pharmaceutical-technology study
533	Burkholderia pseudomallei PenI β-lactamase and variants are potently inhibited by taniborbactam	PubMed	Correction, comment, or reply
534	Novel beta-lactamase inhibitors with cefepime: where do they fit in clinical practice?	PubMed	Other (outside the scope of this review)
535	In vivo activity of ceftibuten-ledaborbactam oral combination against serine-β-lactamase-producing Enterobacterales: an assessment in the pyelonephritis and cystitis murine model	PubMed	In vitro activity or surveillance data
536	Pharmacokinetics, safety, and tolerability of ceftibuten-ledaborbactam etzadroxil in participants with renal impairment	PubMed	PK/PD or pharmaceutical-technology study
537	Broad spectrum of β-lactamase coverage and potent antimicrobial activity of xeruborbactam in combination with meropenem against carbapenemase-producing Enterobacterales, including strains resistant to new β-lactam/β-lactamase inhibitor combinations	PubMed	Combination-therapy study
538	In vitro activity and resistance mechanisms of	PubMed	In vitro activity or surveillance data

	sulbactam/durlobactam against Acinetobacter baumannii clinical isolates in China (2019-2020)		
539	Evaluation of imipenem-relebactam, meropenem-vaborbactam, aztreonam-avibactam and cefepime-zidebactam activities on a wide collection of French clinical Enterobacterales isolates	PubMed	Focused on other β-lactamase inhibitor combinations
540	Plasma and Intrapulmonary Concentrations of ETX2514 and Sulbactam following Intravenous Administration of ETX2514SUL to Healthy Adult Subjects	PubMed	Other (outside the scope of this review)
541	Discovery of Boronic Acids-Based β-Lactamase Inhibitors Through In Situ Click Chemistry	PubMed	Other (outside the scope of this review)
542	Sulbactam-durlobactam and cefiderocol combination treatment of Burkholderia cenocepacia-associated Fitz-Hugh-Curtis syndrome	PubMed	Focused on cefiderocol or other novel agents
543	Discovery of 3-methacylic acid substituted benzoxaboroles as dual metallo- and serine-β-lactamase inhibitors	PubMed	Other (outside the scope of this review)
544	Characterization of Metallo β-Lactamase Producing Enterobacterales Isolates with Susceptibility to the Aztreonam/Avibactam Combination	PubMed	Focused on other β-lactamase inhibitor combinations
545	Fused 3D boron heterocycles via EnT catalysis: synthesis, modification and validation as beta-lactamase inhibitors	PubMed	Structural or chemical-synthesis study
546	The NitroSpeed Taniborbactam NP test as a rapid test for detection of β-lactamase-mediated susceptibility to taniborbactam	PubMed	In vitro activity or surveillance data
547	In Vitro Activity of Cefiderocol against a Global Collection of Carbapenem-Resistant Acinetobacter baumannii Isolates	PubMed	Focused on cefiderocol or other novel agents
548	In vitro activity and resistance mechanisms of novel antimicrobial agents against metallo-β-lactamase producers	PubMed	In vitro activity or surveillance data
549	Sulbactam-durlobactam: A novel β-lactam-β-lactamase inhibitor combination targeting carbapenem-resistant Acinetobacter baumannii infections	PubMed	Other (outside the scope of this review)
550	Cefepime-taniborbactam and ceftibuten-ledaborbactam maintain activity against KPC variants that lead to ceftazidime-avibactam resistance	PubMed	In vitro activity or surveillance data
551	In vitro activity of cefepime/taniborbactam and comparator agents against Gram-negative bacterial bloodstream pathogens recovered from patients with cancer	PubMed	In vitro activity or surveillance data
552	Advancements in the fight against globally distributed OXA-48	PubMed	Other (outside the scope of this review)

	carbapenemase: evaluating the new generation of carbapenemase inhibitors		
553	Pharmacokinetic/Pharmacodynamic Adequacy of Novel β-Lactam/β-Lactamase Inhibitors against Gram-Negative Bacterial in Critically Ill Patients	PubMed	PK/PD or pharmaceutical-technology study
554	2-Carboxyquinoline Boronic Acids as Highly Potent KPC Inhibitors	PubMed	Other (outside the scope of this review)
555	Distributions of plasmidic genes encoding extended-spectrum and AmpC β-lactamases, and susceptibilities of global non-carbapenemase-producing meropenem-resistant Enterobacterales to ceftazidime-avibactam, meropenem-vaborbactam, and aztreonam-avibactam, 2017-2022	PubMed	Focused on other β-lactamase inhibitor combinations
556	In-vitro activity of the novel β-lactam/β-lactamase inhibitor combinations and cefiderocol against carbapenem-resistant Pseudomonas spp. clinical isolates collected in Switzerland in 2022	PubMed	Focused on cefiderocol or other novel agents
557	Population pharmacokinetic analyses for sulbactam-durlobactam using Phase 1, 2, and 3 data	PubMed	PK/PD or pharmaceutical-technology study
558	In vitro activity and genomic characterization of KPC-producing Klebsiella pneumoniae clinical blood culture isolates resistant to ceftazidime/avibactam, meropenem/vaborbactam, imipenem/relebactam: an Italian nationwide multicentre observational study (2022-23)	PubMed	Focused on other β-lactamase inhibitor combinations
559	Characterization of Enterobacter cloacae and Citrobacter freundii Species Complex Isolates with Decreased Susceptibility to Cephalosporins from United States Hospitals and Activity of Aztreonam-Avibactam and Comparator Agents (2019-2023)	PubMed	Focused on other β-lactamase inhibitor combinations
560	Interplay between β-lactamases and new β-lactamase inhibitors	PubMed	Other (outside the scope of this review)
561	Compassionate use of meropenem/vaborbactam for infections caused by KPC-producing Klebsiella pneumoniae: a multicentre study	PubMed	Clinical-outcomes or pharmacoeconomic study
562	Studies on the inhibition of AmpC and other β-lactamases by cyclic boronates	PubMed	Other (outside the scope of this review)
563	Adverse Event Signals Associated with Beta-Lactamase Inhibitors: Disproportionality Analysis of USFDA Adverse Event Reporting System	PubMed	Other (outside the scope of this review)
564	The multiple benefits of second-generation β-lactamase inhibitors in	PubMed	Other (outside the scope of this review)

	treatment of multidrug-resistant bacteria		
565	Meropenem-Vaborbactam as Salvage Therapy for Ceftazidime-Avibactam-Resistant Klebsiella pneumoniae Bacteremia and Abscess in a Liver Transplant Recipient	PubMed	Other (outside the scope of this review)
566	Searching for the Optimal Treatment for Metallo- and Serine-β-Lactamase Producing Enterobacteriaceae: Aztreonam in Combination with Ceftazidime-avibactam or Meropenem-vaborbactam	PubMed	Combination-therapy study
567	Diversity of resistance mechanisms in carbapenem-resistant Enterobacteriaceae at a health care system in Northern California, from 2013 to 2016	PubMed	Other (outside the scope of this review)
568	Optimizing therapy in carbapenem-resistant Enterobacteriaceae infections	PubMed	Other (outside the scope of this review)
569	Pharmacokinetic evaluation of meropenem and vaborbactam for the treatment of urinary tract infection	PubMed	PK/PD or pharmaceutical-technology study
570	In vitro activity of meropenem combined with vaborbactam against KPC-producing Enterobacteriaceae in China	PubMed	Combination-therapy study
571	Inhibiting the β-Lactamase of Mycobacterium tuberculosis (Mtb) with Novel Boronic Acid Transition-State Inhibitors (BATSIs)	PubMed	Mycobacterium-focused
572	Meropenem-vaborbactam: a new weapon in the war against infections due to resistant Gram-negative bacteria	PubMed	Other (outside the scope of this review)
573	Activity of β-Lactamase Inhibitor Combinations Against Enterobacterales Isolated from Patients with Intra-Abdominal Infection from United States Medical Centres (2019-2023)	PubMed	In vitro activity or surveillance data
574	In vitro activity of meropenem/vaborbactam and characterisation of carbapenem resistance mechanisms among carbapenem-resistant Enterobacteriaceae from the 2015 meropenem/vaborbactam surveillance programme	PubMed	In vitro activity or surveillance data
575	Development and in vitro evaluation of 1,4,7-triazacyclononane-coupled β-lactams against metallo-β-lactamase producing bacteria	PubMed	Other (outside the scope of this review)
576	Phenylboronic Acid Derivatives as Validated Leads Active in Clinical Strains Overexpressing KPC-2: A Step against Bacterial Resistance	PubMed	Other (outside the scope of this review)
577	New antibiotics for Gram-negative pneumonia	PubMed	Other (outside the scope of this review)
578	Single-Dose Pharmacokinetics and Safety of Meropenem-Vaborbactam in Subjects with Chronic Renal	PubMed	PK/PD or pharmaceutical-technology study

	Impairment		
579	Inhibition of Acinetobacter-Derived Cephalosporinase: Exploring the Carboxylate Recognition Site Using Novel β-Lactamase Inhibitors	PubMed	Other (outside the scope of this review)
580	Resistance to beta-lactams in Gram-negative bacilli: relevance and potential therapeutic alternatives	PubMed	Other (outside the scope of this review)
581	Vaborbactam: Spectrum of Beta-Lactamase Inhibition and Impact of Resistance Mechanisms on Activity in Enterobacteriaceae	PubMed	Other (outside the scope of this review)
582	Isonitrile-Based Multicomponent Synthesis of β-Amino Boronic Acids as β-Lactamase Inhibitors	PubMed	Structural or chemical-synthesis study
583	Extended and Continuous Infusion of Novel Protected β-Lactam Antibiotics: A Narrative Review	PubMed	General review or guidance (not boronate-focused)
584	Aminoglycosides enhance meropenem/vaborbactam activity against KPC-producing Klebsiella pneumoniae in the hollow fiber infection model	PubMed	In vitro activity or surveillance data
585	Exploring the activity of a novel orally bioavailable β-lactam-β-lactamase inhibitor combination, ceftibuten-ledaborbactam, against Enterobacterales-carrying bla(OXA-48)	PubMed	In vitro activity or surveillance data
586	In vitro activity of cefiderocol and sulbactam-durlobactam against carbapenem-resistant Acinetobacter baumannii clinical isolates collected between 2019 and 2024	PubMed	Focused on cefiderocol or other novel agents
587	Characterization of ertapenem-resistant Enterobacterales in Canadian hospitals: 17 years of the CANWARD study (2007-23)	PubMed	Other (outside the scope of this review)
588	Clinical and microbiological analysis of bloodstream infections by four cefiderocol-resistant and not previously exposed NDM-producing Klebsiella pneumoniae	PubMed	Focused on cefiderocol or other novel agents
589	Rapid detection of cefepime-taniborbactam susceptibility/resistance in Enterobacterales	PubMed	In vitro activity or surveillance data
590	Spectrum of cefepime-taniborbactam coverage against 190 β-lactamases defined in engineered isogenic Escherichia coli strains	PubMed	Other (outside the scope of this review)
591	Are New β-Lactam/β-Lactamase Inhibitor Combinations Promising Against Carbapenem-Resistant K. pneumoniae Isolates?	PubMed	Other (outside the scope of this review)
592	In vitro antimicrobial activity of six novel β-lactam and β-lactamase inhibitor combinations and cefiderocol against NDM-producing Enterobacterales in China	PubMed	Focused on cefiderocol or other novel agents
593	Antimicrobial susceptibility of	PubMed	Focused on other β-

	enterobacterales causing bloodstream infection in United States medical centres: comparison of aztreonam-avibactam with beta-lactams active against carbapenem-resistant enterobacterales		lactamase inhibitor combinations
594	Multicenter evaluation of activity of aztreonam in combination with avibactam, relebactam, and vaborbactam against metallo-β-lactamase-producing carbapenem-resistant gram-negative bacilli	PubMed	Combination-therapy study
595	Relative inhibitory activities of the broad-spectrum β-lactamase inhibitor xeruborbactam in comparison with taniborbactam against metallo-β-lactamases produced in Escherichia coli and Pseudomonas aeruginosa	PubMed	Other (outside the scope of this review)
596	Relative inhibitory activities of the broad-spectrum β-lactamase inhibitor taniborbactam against metallo-β-lactamases	PubMed	Other (outside the scope of this review)
597	Determination of potential combination of non-β-lactam, β-lactam, and β-lactamase inhibitors/β-lactam enhancer against class D oxacillinases producing Acinetobacter baumannii: Evidence from in-vitro, molecular docking and dynamics simulation	PubMed	Structural or chemical-synthesis study
598	Ceftazidime/avibactam-resistant meropenem-susceptible KPC-producing Klebsiella pneumoniae: Analysis of cases and evaluation of in vitro activity of fosfomycin-containing combinations	PubMed	In vitro activity or surveillance data
599	Ceftazidime-avibactam, meropenem-vaborbactam, and imipenem-relebactam activities against multidrug-resistant Enterobacterales from United States Medical Centers (2018-2022)	PubMed	Focused on other β-lactamase inhibitor combinations
600	Impact of Minor Carbapenemases on Susceptibility to Novel β-Lactam/β-Lactamase Inhibitor Combinations and Cefiderocol in Enterobacterales	PubMed	Focused on cefiderocol or other novel agents
601	In vitro activity of cefepime/zidebactam and cefepime/taniborbactam against aztreonam/avibactam-resistant NDM-like-producing Escherichia coli clinical isolates	PubMed	Focused on other β-lactamase inhibitor combinations
602	Activity of aztreonam in combination with novel β-lactamase inhibitors against metallo-β-lactamase-producing Enterobacterales from Spain	PubMed	Combination-therapy study
603	Meropenem-Vaborbactam Activity against U.S. Multidrug-Resistant Enterobacterales Strains, Including Carbapenem-Resistant Isolates	PubMed	In vitro activity or surveillance data
604	Deciphering variable resistance to	PubMed	Resistance-mechanism

	novel carbapenem-based β-lactamase inhibitor combinations in a multi-clonal outbreak caused by Klebsiella pneumoniae carbapenemase (KPC)-producing Klebsiella pneumoniae resistant to ceftazidime/avibactam		or variant study
605	Geographic patterns of global isolates of carbapenem-resistant Klebsiella pneumoniae and the activity of ceftazidime/avibactam, meropenem/vaborbactam, and comparators against these isolates: Results from the Antimicrobial Testing Leadership and Surveillance (ATLAS) program, 2020	PubMed	In vitro activity or surveillance data
606	Activity of cefiderocol, imipenem/relebactam, cefepime/taniborbactam and cefepime/zidebactam against ceftolozane/tazobactam- and ceftazidime/avibactam-resistant Pseudomonas aeruginosa	PubMed	Focused on cefiderocol or other novel agents
607	Comparing the activity of novel antibiotic agents against carbapenem-resistant Enterobacterales clinical isolates	PubMed	In vitro activity or surveillance data
608	Evaluation of the inoculum effect of new antibiotics against carbapenem-resistant enterobacterales	PubMed	Other (outside the scope of this review)
609	The Ins and Outs of Susceptibility Testing for New β-Lactam/β-Lactamase Inhibitor Combinations for Gram-Negative Organisms	PubMed	In vitro activity or surveillance data
610	Structural basis to repurpose boron-based proteasome inhibitors Bortezomib and Ixazomib as β-lactamase inhibitors	PubMed	Other (outside the scope of this review)
611	Three new inhibitors of class A β-lactamases evaluated by molecular docking and dynamics simulations methods: relebactam, enmetazobactam, and QPX7728	PubMed	Focused on other β-lactamase inhibitor combinations
612	Inhibiting Mycobacterium abscessus Cell Wall Synthesis: Using a Novel Diazabicyclooctane β-Lactamase Inhibitor To Augment β-Lactam Action	PubMed	Mycobacterium-focused
613	In Vitro Activity of Cefepime-Taniborbactam against Carbapenemase-Producing Enterobacterales and Pseudomonas aeruginosa Isolates Recovered in Spain	PubMed	In vitro activity or surveillance data
614	New options for bloodstream infections caused by colistin- or ceftazidime/avibactam-resistant Klebsiella pneumoniae	PubMed	Other (outside the scope of this review)
615	Multicenter Clinical Evaluation of Vitek 2 Meropenem-Vaborbactam for Susceptibility Testing of Enterobacterales and Pseudomonas aeruginosa	PubMed	In vitro activity or surveillance data

616	In Vitro Activity of Ceftibuten/VNRX-5236 against Urinary Tract Infection Isolates of Antimicrobial-Resistant Enterobacterales	PubMed	In vitro activity or surveillance data
617	Activity of meropenem/vaborbactam and comparators against Gram-negative isolates from Eastern and Western European patients hospitalized with pneumonia including ventilator-associated pneumonia (2014-19)	PubMed	In vitro activity or surveillance data
618	Population Pharmacokinetics of Meropenem and Vaborbactam Based on Data from Noninfected Subjects and Infected Patients	PubMed	PK/PD or pharmaceutical-technology study
619	Microbiological Characterization of VNRX-5236, a Broad-Spectrum β-Lactamase Inhibitor for Rescue of the Orally Bioavailable Cephalosporin Ceftibuten as a Carbapenem-Sparing Agent against Strains of Enterobacterales Expressing Extended-Spectrum β-Lactamases and Serine Carbapenemases	PubMed	Other (outside the scope of this review)
620	Assessing the Potency of β-Lactamase Inhibitors with Diverse Inactivation Mechanisms against the PenA1 Carbapenemase from Burkholderia multivorans	PubMed	Other (outside the scope of this review)
621	In vivo pharmacodynamics of new-generation β-lactamase inhibitor taniborbactam (formerly VNRX-5133) in combination with cefepime against serine-β-lactamase-producing Gram-negative bacteria	PubMed	PK/PD or pharmaceutical-technology study
622	Bicyclic Boronates as Potent Inhibitors of AmpC, the Class C β-Lactamase from Escherichia coli	PubMed	Other (outside the scope of this review)
623	α-Triazolylboronic Acids: A Promising Scaffold for Effective Inhibitors of KPCs	PubMed	Other (outside the scope of this review)
624	An Update on Existing and Emerging Data for Meropenem-Vaborbactam	PubMed	General review or guidance (not boronate-focused)
625	Activity of Imipenem-Relebactam and Meropenem-Vaborbactam against Carbapenem-Resistant, SME-Producing Serratia marcescens	PubMed	Focused on other β-lactamase inhibitor combinations
626	Broad Spectrum β-Lactamase Inhibition by a Thioether Substituted Bicyclic Boronate	PubMed	Other (outside the scope of this review)
627	Serendipitous discovery of aryl boronic acids as β-lactamase inhibitors	PubMed	Other (outside the scope of this review)
628	A validated LC-MSMS method for the simultaneous quantification of meropenem and vaborbactam in human plasma and renal replacement therapy effluent and its application to a pharmacokinetic study	PubMed	PK/PD or pharmaceutical-technology study
629	Meropenem-Vaborbactam Activity against Carbapenem-Resistant	PubMed	In vitro activity or surveillance data

	Enterobacterales Isolates Collected in U.S. Hospitals during 2016 to 2018		
630	Biochemical Activity of Vaborbactam	PubMed	In vitro activity or surveillance data
631	Efficacy and Safety of Meropenem-Vaborbactam Versus Best Available Therapy for the Treatment of Carbapenem-Resistant Enterobacteriaceae Infections in Patients Without Prior Antimicrobial Failure: A Post Hoc Analysis	PubMed	Other (outside the scope of this review)
632	Targeting Multidrug-Resistant Acinetobacter spp.: Sulbactam and the Diazabicyclooctenone β-Lactamase Inhibitor ETX2514 as a Novel Therapeutic Agent	PubMed	Other (outside the scope of this review)
633	The Infinity War: How to Cope with Carbapenem-resistant Enterobacteriaceae	PubMed	Other (outside the scope of this review)
634	Meropenem-vaborbactam for the treatment of complicated urinary tract infections including acute pyelonephritis	PubMed	Other (outside the scope of this review)
635	Boronic acid inhibitors of the class A β-lactamase KPC-2	PubMed	Other (outside the scope of this review)
636	Structure-based approach for identification of novel phenylboronic acids as serine-β-lactamase inhibitors	PubMed	Structural or chemical-synthesis study
637	Meropenem-vaborbactam: a carbapenem and beta-lactamase inhibitor with activity against carbapenem-resistant Enterobacteriaceae	PubMed	In vitro activity or surveillance data
638	Head-to-head: meropenem/vaborbactam versus ceftazidime/avibactam in ICUs patients with KPC-producing K. pneumoniae infections- results from a retrospective multicentre study	PubMed	Clinical-outcomes or pharmacoeconomic study
639	New β-Lactamase Inhibitors in the Clinic	PubMed	Other (outside the scope of this review)
640	Frequency and Mechanism of Spontaneous Resistance to Sulbactam Combined with the Novel β-Lactamase Inhibitor ETX2514 in Clinical Isolates of Acinetobacter baumannii	PubMed	Combination-therapy study
641	Comparative evaluation of the activity of novel β-lactam antibiotics against difficult-to-treat Pseudomonas aeruginosa clinical isolates	PubMed	In vitro activity or surveillance data
642	Reversibility of Covalent, Broad-Spectrum Serine β-Lactamase Inhibition by the Diazabicyclooctenone ETX2514	PubMed	Other (outside the scope of this review)
643	Structure-based enhancement of boronic acid-based inhibitors of AmpC beta-lactamase	PubMed	Structural or chemical-synthesis study
644	Comparative activity of ceftibuten combinations with avibactam, ledaborbactam, and xeruborbactam	PubMed	In vitro activity or surveillance data

	against recombinant β-lactamase-producing Escherichia coli and contemporary WGS-characterized strains of carbapenemase-producing Enterobacterales		
645	Computational and biological profile of boronic acids for the detection of bacterial serine- and metallo-β-lactamases	PubMed	Detection or diagnostic methodology
646	Permeation through the cell membrane of a boron-based β-lactamase inhibitor	PubMed	Other (outside the scope of this review)
647	AMBER force field implementation of the boronate function to simulate the inhibition of beta-lactamases by alkyl and aryl boronic acids	PubMed	Other (outside the scope of this review)
648	Crystal structure of Enterobacter cloacae 908R class C beta-lactamase bound to iodo-acetamido-phenyl boronic acid, a transition-state analogue	PubMed	Database record (non-research item)
649	Do inert beta-lactamase inhibitors act as synergizers of beta-lactam antibiotics? Utility of boric and boronic acids	PubMed	Other (outside the scope of this review)
650	Use of boronic acid disk tests to detect extended- spectrum beta-lactamases in clinical isolates of KPC carbapenemase-possessing enterobacteriaceae	PubMed	Detection or diagnostic methodology
651	Structure and dynamics of CTX-M enzymes reveal insights into substrate accommodation by extended-spectrum beta-lactamases	PubMed	Structural or chemical-synthesis study
652	The deacylation mechanism of AmpC beta-lactamase at ultrahigh resolution	PubMed	Other (outside the scope of this review)
653	Thoracic aorta graft infection by avibactam-resistant KPC-producing K. pneumoniae treated with meropenem/vaborbactam: a case report and literature review	PubMed	Case report
654	Novel Insights into Carbapenem Resistance: Mechanisms, Diagnostics, and Future Directions	PubMed	Other (outside the scope of this review)
655	Antibiotic Treatment of Severe Infections with Multiresistant Bacteria	PubMed	Other (outside the scope of this review)
656	Meropenem/vaborbactam-based combinations against KPC-producing Klebsiella pneumoniae and multidrug-resistant Pseudomonas aeruginosa	PubMed	Other (outside the scope of this review)
657	Carbapenem-resistant Enterobacterales across the UK: A nationwide study of carbapenemase testing and novel antimicrobial activity	PubMed	Detection or diagnostic methodology
658	Consistent in vitro activity of sulbactam-durlobactam against genetically diverse carbapenem-resistant Acinetobacter baumannii in a high-endemic Brazilian setting	PubMed	In vitro activity or surveillance data
659	Therapeutic challenges in treating ESBL- and/or AmpC-producing non-carbapenemase-producing	PubMed	Focused on cefiderocol or other novel agents

	Enterobacterales: an in vitro evaluation of novel β-lactam/β-lactamase inhibitor combinations and cefiderocol		
660	Global susceptibility profiles and potential resistance mechanisms of ceftazidime-avibactam-resistant, non-carbapenemase-producing carbapenem-resistant Enterobacterales: 2020-23 data from the Antimicrobial Testing Leadership and Surveillance	PubMed	In vitro activity or surveillance data
661	In vitro activity of meropenem-vaborbactam combinations and eravacycline against carbapenem-resistant Acinetobacter baumannii	PubMed	In vitro activity or surveillance data
662	Sporadic cefiderocol resistance in Escherichia coli from the United Arab Emirates involves multifactorial mechanisms reversible by novel beta-lactamase inhibitors	PubMed	Focused on cefiderocol or other novel agents
663	Non-Klebsiella pneumoniae Carbapenemase Attributes of the Newer β-lactam/β-lactamase Inhibitors, Part 2: Burkholderia cepacia Complex, Stenotrophomonas maltophilia, and Acinetobacter baumannii	PubMed	Other (outside the scope of this review)
664	Challenges of Carbapenem-Resistant Enterobacteriaceae in the Development of New β-Lactamase Inhibitors and Antibiotics	PubMed	Other (outside the scope of this review)
665	Antimicrobial activity of new anti-Pseudomonas beta-lactam-beta-lactamase inhibitors against Pseudomonas aeruginosa respiratory isolates recovered during the study for Monitoring Antimicrobial Resistance Trends (SMART) program in France (2016-2022)	PubMed	In vitro activity or surveillance data
666	In vitro effects of the new oral β-lactamase inhibitor xeruborbactam in combination with oral β-lactams against clinical Mycobacterium abscessus isolates	PubMed	Mycobacterium-focused
667	Successful Treatment of Carbapenem-Resistant Acinetobacter baumannii Meningitis With Sulbactam-Durlobactam	PubMed	Other (outside the scope of this review)
668	What are the optimal pharmacokinetic/pharmacodynamic targets for β-lactamase inhibitors? A systematic review	PubMed	PK/PD or pharmaceutical-technology study
669	The metallo-β-lactamases strike back: emergence of taniborbactam escape variants	PubMed	Other (outside the scope of this review)
670	The occurrence of the carbapenemase gene, bla(NDM-5), on a transmissible IncX3 plasmid in multidrug-resistant Escherichia coli isolated from a farm dog	PubMed	Other (outside the scope of this review)

671	Meropenem/Vaborbactam: β-Lactam/β-Lactamase Inhibitor Combination, the Future in Eradicating Multidrug Resistance	PubMed	Other (outside the scope of this review)
672	A randomized, double-blind, placebo- and positive-controlled crossover study of the effects of durlobactam on cardiac repolarization in healthy subjects	PubMed	Other (outside the scope of this review)
673	In vitro activities of ceftazidime-avibactam, ceftolozane-tazobactam, meropenem-vaborbactam and other comparators against Pseudomonas aeruginosa isolates with discrepant resistance to carbapenems: Data from the Antimicrobial Testing Leadership and Surveillance (ATLAS) program, 2012-2021	PubMed	Focused on other β-lactamase inhibitor combinations
674	In vitro susceptibility of carbapenem resistant Enterobacterales to meropenem-vaborbactam and ceftazidime-avibactam at a single academic medical centre	PubMed	In vitro activity or surveillance data
675	[New antimicrobial substances to combat increasing resistance]	PubMed	Other (outside the scope of this review)
676	Omic insights into various ceftazidime-avibactam-resistant Klebsiella pneumoniae isolates from two southern Italian regions	PubMed	Other (outside the scope of this review)
677	In vitro-obtained meropenem-vaborbactam resistance mechanisms among clinical Klebsiella pneumoniae carbapenemase-producing K. pneumoniae isolates	PubMed	Other (outside the scope of this review)
678	Phenotypes, genotypes and breakpoints: an assessment of β-lactam/β-lactamase inhibitor combinations against OXA-48	PubMed	Detection or diagnostic methodology
679	In-vitro activity of plazomicin, meropenem-vaborbactam, and omadacycline against carbapenem-resistant Gram-negative isolates in Egypt	PubMed	In vitro activity or surveillance data
680	Recent Developments to Cope the Antibacterial Resistance via β-Lactamase Inhibition	PubMed	Other (outside the scope of this review)
681	Carbapenemase-producing Enterobacterales infections: recent advances in diagnosis and treatment	PubMed	Other (outside the scope of this review)
682	Synthesis and Application of Constrained Amidoboronic Acids Using Amphoteric Boron-Containing Building Blocks	PubMed	Structural or chemical-synthesis study
683	First report of coexistence of AmpC beta-lactamase genes in Klebsiella pneumoniae strains isolated from burn patients	PubMed	Other (outside the scope of this review)
684	The combined-disk boronic acid test as an accurate strategy for the detection of KPC carbapenemase in Central	PubMed	Detection or diagnostic methodology

	America		
685	Boronic Acid Transition State Inhibitors Active against KPC and Other Class A β-Lactamases: Structure-Activity Relationships as a Guide to Inhibitor Design	PubMed	Structural or chemical-synthesis study
686	A resurgence of β-lactamase inhibitor combinations effective against multidrug-resistant Gram-negative pathogens	PubMed	Other (outside the scope of this review)
687	Effect of an amino acid insertion into the omega loop region of a class C beta-lactamase on its substrate specificity	PubMed	Resistance-mechanism or variant study
688	β-Lactamase inhibitors: a review of the patent literature (2010 - 2013)	PubMed	General review or guidance (not boronate-focused)
689	The use of cefepime for treating AmpC β-lactamase-producing Enterobacteriaceae	PubMed	Other (outside the scope of this review)
690	Activity of biapenem (RPX2003) combined with the boronate β-lactamase inhibitor RPX7009 against carbapenem-resistant Enterobacteriaceae	PubMed	Combination-therapy study
691	Comparison of the boronic acid disk potentiation test and cefepime-clavulanic acid method for the detection of ESBL among AmpC-producing Enterobacteriaceae	PubMed	Detection or diagnostic methodology
692	Practical methods using boronic acid compounds for identification of class C beta-lactamase-producing Klebsiella pneumoniae and Escherichia coli	PubMed	Other (outside the scope of this review)
693	Mechanism of reaction of acyl phosph(on)ates with the beta-lactamase of Enterobacter cloacae P99	PubMed	Other (outside the scope of this review)
694	Broth microdilution method to detect extended-spectrum beta-lactamases and AmpC beta-lactamases in enterobacteriaceae isolates by use of clavulanic acid and boronic acid as inhibitors	PubMed	Detection or diagnostic methodology
695	First occurrence of KPC-2-possessing Klebsiella pneumoniae in a Greek hospital and recommendation for detection with boronic acid disc tests	PubMed	Detection or diagnostic methodology
696	Detection of extended-spectrum beta-lactamases by using boronic acid as an AmpC beta-lactamase inhibitor in clinical isolates of Klebsiella spp. and Escherichia coli	PubMed	Detection or diagnostic methodology
697	In vitro activity of β-lactamase inhibitors avibactam, relebactam and vaborbactam in combination with aztreonam against metallo-β-lactamase producing Escherichia coli clinical isolates	PubMed	Combination-therapy study
698	Genomic and molecular characterisation of a KPC-producing Klebsiella pneumoniae clinical isolate	PubMed	Focused on other β-lactamase inhibitor combinations

	resistant to meropenem-vaborbactam, imipenem-relebactam, and ceftazidime-avibactam		
699	Aggressive joint pharmacokinetic/pharmacodynamic target attainment of TDM-guided continuous infusion meropenem-vaborbactam monotherapy: A valuable strategy for maximizing the microbiological outcome of documented KPC-producing Enterobacterales infections?	PubMed	PK/PD or pharmaceutical-technology study
700	Structural and Kinetic Basis for the Rational Design of Next-Generation β-Lactamase Inhibitors	PubMed	Other (outside the scope of this review)
701	Failure of durlobactam to restore the susceptibility to meropenem in carbapenem-resistant Klebsiella pneumoniae	PubMed	In vitro activity or surveillance data
702	Pharmacokinetics/pharmacodynamics of KSP-1007 in combination with meropenem against carbapenemase-producing gram-negative bacteria in a neutropenic murine thigh infection model	PubMed	PK/PD or pharmaceutical-technology study
703	Emerging resistance mechanisms to newer β-lactams in Pseudomonas aeruginosa	PubMed	Other (outside the scope of this review)
704	Whole-genome sequencing characterisation of a new KPC-245 variant-carrying Klebsiella pneumoniae strain isolated from a transplanted patient and resistant to ceftazidime/avibactam, meropenem/vaborbactam and imipenem/relebactam	PubMed	Focused on other β-lactamase inhibitor combinations
705	Restoring cefepime activity against multidrug-resistant KPC-producing Klebsiella pneumoniae by combination with boronic acid inhibitors, MB076 and S02030	PubMed	In vitro activity or surveillance data
706	Identification of bla(KPC-90) in Klebsiella pneumoniae associated with ceftazidime-avibactam resistance and the translocation & truncation of resistant genes mediated by IS26	PubMed	Other (outside the scope of this review)
707	In vitro activity of cefepime-enmetazobactam on carbapenem-resistant Gram negatives	PubMed	Focused on other β-lactamase inhibitor combinations
708	Cefepime-taniborbactam activity against antimicrobial-resistant clinical isolates of Enterobacterales and Pseudomonas aeruginosa: GEARS global surveillance programme 2018-22	PubMed	In vitro activity or surveillance data
709	ARGONAUT-III and -V: susceptibility of carbapenem-resistant Klebsiella pneumoniae and multidrug-resistant Pseudomonas aeruginosa to the bicyclic boronate β-lactamase inhibitor	PubMed	Combination-therapy study

	taniborbactam combined with cefepime		
710	Biliary pharmacokinetic/pharmacodynamic analysis of continuous infusion meropenem/vaborbactam in a case series of orthotopic liver transplant recipients	PubMed	Case report
711	In vitro and in vivo activities of KSP-1007, a broad-spectrum inhibitor of serine- and metallo-β-lactamases, in combination with meropenem against carbapenem-resistant Gram-negative bacteria	PubMed	Combination-therapy study
712	Antimicrobial activity of ceftazidime-avibactam against KPC-2-producing Enterobacterales: a cross-combination and dose-escalation titration study with relebactam and vaborbactam	PubMed	In vitro activity or surveillance data
713	In vitro activity of ceftazidime/avibactam, cefiderocol, meropenem/vaborbactam and imipenem/relebactam against clinical strains of the Stenotrophomonas maltophilia complex	PubMed	Focused on cefiderocol or other novel agents
714	Durlobactam, a Diazabicyclooctane β-Lactamase Inhibitor, Inhibits BlaC and Peptidoglycan Transpeptidases of Mycobacterium tuberculosis	PubMed	Mycobacterium-focused
715	Metallo-β-lactamase inhibitors: A continuing challenge for combating antibiotic resistance	PubMed	Other (outside the scope of this review)
716	In vitro activity of cefiderocol against European Pseudomonas aeruginosa and Acinetobacter spp., including isolates resistant to meropenem and recent β-lactam/β-lactamase inhibitor combinations	PubMed	Focused on cefiderocol or other novel agents
717	In vitro activity of meropenem-vaborbactam plus aztreonam against metallo-β-lactamase-producing Klebsiella pneumoniae	PubMed	Combination-therapy study
718	Assessing the in vivo impact of novel β-lactamase inhibitors on the efficacy of their partner β-lactams against serine carbapenemase-producing Pseudomonas aeruginosa using human-simulated exposures	PubMed	Other (outside the scope of this review)
719	Natural protein engineering in the Ω-loop: the role of Y221 in ceftazidime and ceftolozane resistance in Pseudomonas-derived cephalosporinase	PubMed	Focused on other β-lactamase inhibitor combinations
720	Meropenem-vaborbactam restoration of first-line drug efficacy and comparison of meropenem-vaborbactam-moxifloxacin versus BPaL MDR-TB regimen	PubMed	Mycobacterium-focused
721	Comparative Activities of Novel Therapeutic Agents against Molecularly	PubMed	Other (outside the scope of this review)

	Characterized Clinical Carbapenem-Resistant Enterobacterales Isolates		
722	Durlobactam, a Broad-Spectrum Serine β-lactamase Inhibitor, Restores Sulbactam Activity Against Acinetobacter Species	PubMed	In vitro activity or surveillance data
723	The Pharmacokinetics/Pharmacodynamic Relationship of Durlobactam in Combination With Sulbactam in In Vitro and In Vivo Infection Model Systems Versus Acinetobacter baumannii-calcoaceticus Complex	PubMed	PK/PD or pharmaceutical-technology study
724	In Vitro Activity of Cefepime-Taniborbactam and Comparators against Clinical Isolates of Gram-Negative Bacilli from 2018 to 2020: Results from the Global Evaluation of Antimicrobial Resistance via Surveillance (GEARS) Program	PubMed	In vitro activity or surveillance data
725	In vitro activity of aztreonam in combination with newly developed β-lactamase inhibitors against MDR Enterobacterales and Pseudomonas aeruginosa producing metallo-β-lactamases	PubMed	Combination-therapy study
726	In vivo pharmacokinetics and pharmacodynamics of ceftibuten/ledaborbactam, a novel oral β-lactam/β-lactamase inhibitor combination	PubMed	PK/PD or pharmaceutical-technology study
727	Susceptibility of OXA-48-producing Enterobacterales to imipenem/relebactam, meropenem/vaborbactam and ceftazidime/avibactam	PubMed	Focused on other β-lactamase inhibitor combinations
728	Safety and Pharmacokinetics of Taniborbactam (VNRX-5133) with Cefepime in Subjects with Various Degrees of Renal Impairment	PubMed	PK/PD or pharmaceutical-technology study
729	Activity of meropenem-vaborbactam against different beta-lactamase producing Klebsiella pneumoniae and Escherichia coli isolates in Iran	PubMed	In vitro activity or surveillance data
730	A rationale for the unlike potency of avibactam and ETX2514 against OXA-24 β-lactamase	PubMed	Other (outside the scope of this review)
731	Genome characterization of a Klebsiella pneumoniae co-producing OXA-181 and KPC-121 resistant to ceftazidime/avibactam, meropenem/vaborbactam, imipenem/relebactam and cefiderocol isolated from a critically ill patient	PubMed	Focused on cefiderocol or other novel agents
732	Activity of cefiderocol and synergy of novel β-lactam-β-lactamase inhibitor-based combinations against metallo-β-lactamase-producing gram-negative bacilli: insights from a two-year study (2019-2020)	PubMed	Focused on cefiderocol or other novel agents

733	Phenotypes, genotypes and breakpoints: an assessment of β-lactam/ β-lactamase inhibitor combinations against OXA-48	PubMed	Detection or diagnostic methodology
734	In vitro antibacterial activity of cefiderocol against recent multidrug-resistant carbapenem-nonsusceptible Enterobacterales isolates	PubMed	Focused on cefiderocol or other novel agents
735	Clinical exposure-response relationship of cefepime/taniborbactam against Gram-negative organisms in the murine complicated urinary tract infection model	PubMed	Other (outside the scope of this review)
736	Antimicrobial activities of ceftazidime/avibactam, ceftolozane/tazobactam, imipenem/relebactam, meropenem/vaborbactam, and comparators against Pseudomonas aeruginosa from patients with skin and soft tissue infections	PubMed	Focused on other β-lactamase inhibitor combinations
737	In vitro comparative activity of the new beta-lactamase inhibitor taniborbactam with cefepime or meropenem against Klebsiella pneumoniae and cefepime against Pseudomonas aeruginosa metallo-beta-lactamase-producing clinical isolates	PubMed	In vitro activity or surveillance data
738	Discovery of VNRX-7145 (VNRX-5236 Etzadroxil): An Orally Bioavailable β-Lactamase Inhibitor for Enterobacterales Expressing Ambler Class A, C, and D Enzymes	PubMed	Other (outside the scope of this review)
739	Carbapenemase producing Klebsiella pneumoniae: implication on future therapeutic strategies	PubMed	Other (outside the scope of this review)
740	Activity of meropenem/vaborbactam against international carbapenem-resistant Escherichia coli isolates in relation to clonal background, resistance genes, resistance to comparators and region	PubMed	In vitro activity or surveillance data
741	Activity of β-lactam/taniborbactam (VNRX-5133) combinations against carbapenem-resistant Gram-negative bacteria	PubMed	In vitro activity or surveillance data
742	Efficacy of human-simulated exposures of meropenem/vaborbactam and meropenem against OXA-48 β-lactamase-producing Enterobacterales in the neutropenic murine thigh infection model	PubMed	Other (outside the scope of this review)
743	An evaluation of meropenem/vaborbactam for the treatment of nosocomial pneumonia	PubMed	Other (outside the scope of this review)
744	Activity of Aztreonam in Combination with Avibactam, Clavulanate, Relebactam, and Vaborbactam against Multidrug-Resistant	PubMed	Combination-therapy study

	Stenotrophomonas maltophilia		
745	In Vitro Activity of the Ultrabroad-Spectrum-Beta-Lactamase Inhibitor QPX7728 against Carbapenem-Resistant Enterobacterales with Varying Intrinsic and Acquired Resistance Mechanisms	PubMed	In vitro activity or surveillance data
746	Impact of Intrinsic Resistance Mechanisms on Potency of QPX7728, a New Ultrabroad-Spectrum Beta-Lactamase Inhibitor of Serine and Metallo-Beta-Lactamases in Enterobacteriaceae, Pseudomonas aeruginosa, and Acinetobacter baumannii	PubMed	Other (outside the scope of this review)
747	Discovery of Cyclic Boronic Acid QPX7728, an Ultrabroad-Spectrum Inhibitor of Serine and Metallo-β-lactamases	PubMed	Other (outside the scope of this review)
748	Evaluation of Hemodialysis Effect on Pharmacokinetics of Meropenem/Vaborbactam in End-Stage Renal Disease Patients Using Modeling and Simulation	PubMed	PK/PD or pharmaceutical-technology study
749	β-Lactamase of Mycobacterium tuberculosis Shows Dynamics in the Active Site That Increase upon Inhibitor Binding	PubMed	Mycobacterium-focused
750	New Drugs for Multidrug-Resistant Gram-Negative Organisms: Time for Stewardship	PubMed	Other (outside the scope of this review)
751	In Vitro Activity of the New β-Lactamase Inhibitors Relebactam and Vaborbactam in Combination with β-Lactams against Mycobacterium abscessus Complex Clinical Isolates	PubMed	Mycobacterium-focused
752	Pharmacokinetics/Pharmacodynamics of Vaborbactam, a Novel Beta-Lactamase Inhibitor, in Combination with Meropenem	PubMed	PK/PD or pharmaceutical-technology study
753	Meropenem-vaborbactam for adults with complicated urinary tract and other invasive infections	PubMed	Other (outside the scope of this review)
754	The antibiotic arms race: current and emerging therapy for Klebsiella pneumoniae carbapenemase (KPC) -producing bacteria	PubMed	Other (outside the scope of this review)
755	Meropenem and Vaborbactam: Stepping up the Battle against Carbapenem-resistant Enterobacteriaceae	PubMed	Other (outside the scope of this review)
756	Determination of MIC and disk diffusion quality control guidelines for meropenem-vaborbactam, a novel carbapenem/boronic acid β-lactamase inhibitor combination	PubMed	General review or guidance (not boronate-focused)
757	Imipenem-Relebactam and Meropenem-Vaborbactam: Two Novel Carbapenem-β-Lactamase Inhibitor Combinations	PubMed	Focused on other β-lactamase inhibitor combinations

758	Structure-Based Analysis of Boronic Acids as Inhibitors of Acinetobacter-Derived Cephalosporinase-7, a Unique Class C β-Lactamase	PubMed	Structural or chemical-synthesis study
759	Activity of Meropenem-Vaborbactam in Mouse Models of Infection Due to KPC-Producing Carbapenem-Resistant Enterobacteriaceae	PubMed	In vitro activity or surveillance data
760	In Vitro Activity of Meropenem-Vaborbactam against Clinical Isolates of KPC-Positive Enterobacteriaceae	PubMed	In vitro activity or surveillance data
761	ETX2514 is a broad-spectrum β-lactamase inhibitor for the treatment of drug-resistant Gram-negative bacteria including Acinetobacter baumannii	PubMed	Other (outside the scope of this review)
762	Cyclic Boronates Inhibit All Classes of β-Lactamases	PubMed	Other (outside the scope of this review)
763	Exploring the potential of boronic acids as inhibitors of OXA-24/40 β-lactamase	PubMed	Other (outside the scope of this review)
764	Boronic acid disk diffusion for the phenotypic detection of polymerase chain reaction-confirmed, carbapenem-resistant, gram-negative bacilli isolates	PubMed	Detection or diagnostic methodology
765	Crystal Structures of KPC-2 and SHV-1 β-Lactamases in Complex with the Boronic Acid Transition State Analog S02030	PubMed	Structural or chemical-synthesis study
766	Structure, function, and inhibition along the reaction coordinate of CTX-M beta-lactamases	PubMed	Structural or chemical-synthesis study
767	Thermodynamic evaluation of a covalently bonded transition state analogue inhibitor: inhibition of beta-lactamases by phosphonates	PubMed	Other (outside the scope of this review)
768	Characterization of beta-lactamases responsible for resistance to extended-spectrum cephalosporins in Escherichia coli and Salmonella enterica strains from food-producing animals in the United Kingdom	PubMed	Other (outside the scope of this review)
769	Recognition and resistance in TEM beta-lactamase	PubMed	Other (outside the scope of this review)
770	Structure-based design guides the improved efficacy of deacylation transition state analogue inhibitors of TEM-1 beta-Lactamase(,)	PubMed	Structural or chemical-synthesis study
771	A disc diffusion assay for detection of class A, B and OXA-48 carbapenemases in Enterobacteriaceae using phenyl boronic acid, dipicolinic acid and temocillin	PubMed	Detection or diagnostic methodology
772	Interactions of oximino-substituted boronic acids and β-lactams with the CMY-2-derived extended-spectrum cephalosporinases CMY-30 and CMY-42	PubMed	Other (outside the scope of this review)
773	A simple test for the detection of KPC and metallo-β-lactamase carbapenemase-producing Pseudomonas aeruginosa isolates with	PubMed	Detection or diagnostic methodology

	the use of meropenem disks supplemented with aminophenylboronic acid, dipicolinic acid and cloxacillin		
774	Optimal phenotypic testing of AmpC beta-lactamases using boronic acid solutions	PubMed	Detection or diagnostic methodology
775	Controlling false-positive results obtained with the Hodge and Masuda assays for detection of class a carbapenemase in species of enterobacteriaceae by incorporating boronic Acid	PubMed	Detection or diagnostic methodology
776	Interplay of efflux, impermeability, and AmpC activity contributes to cefuroxime resistance in clinical, non-ESBL-producing isolates of Escherichia coli	PubMed	Other (outside the scope of this review)
777	Evaluation of boronic acid disk tests for differentiating KPC-possessing Klebsiella pneumoniae isolates in the clinical laboratory	PubMed	Detection or diagnostic methodology
778	Boronic acid disk tests for identification of extended-spectrum beta-lactamase production in clinical isolates of Enterobacteriaceae producing chromosomal AmpC beta-lactamases	PubMed	Detection or diagnostic methodology
779	Structure-based optimization of cephalothin-analogue boronic acids as beta-lactamase inhibitors	PubMed	Structural or chemical-synthesis study
780	Structures of ceftazidime and its transition-state analogue in complex with AmpC beta-lactamase: implications for resistance mutations and inhibitor design	PubMed	Structural or chemical-synthesis study
781	Clinico-Microbiological Profile of Carbapenem-Resistant Acinetobacter baumannii Isolates From Intensive Care Unit Patients and In Vitro Activity of Sulbactam-Durlobactam: A Cross-Sectional Study From Eastern India	PubMed	In vitro activity or surveillance data
782	Successful Treatment of Bloodstream Infection due to a KPC-Producing Klebsiella Pneumoniae Resistant to Imipenem/Relebactam in a Hematological Patient	PubMed	Focused on other β-lactamase inhibitor combinations
783	Novel Antibiotics and Emerging Resistance: In Vitro Activity of Five Antimicrobials Against Intensive Care Unit Isolates from a Turkish Tertiary Care Hospital	PubMed	In vitro activity or surveillance data
784	Pharmacokinetics, Pharmacodynamics, and Dosing Considerations of Novel β-Lactams and β-Lactam/β-Lactamase Inhibitors in Critically Ill Adult Patients: Focus on Obesity, Augmented Renal Clearance, Renal Replacement Therapies, and Extracorporeal	PubMed	PK/PD or pharmaceutical-technology study

	Membrane Oxygenation		
785	Simple disk-based method for detection of Klebsiella pneumoniae carbapenemase-type beta-lactamase by use of a boronic acid compound	PubMed	Detection or diagnostic methodology
786	Use of novel boronic acid transition state inhibitors to probe substrate affinity in SHV-type extended-spectrum beta-lactamases	PubMed	Other (outside the scope of this review)
787	Broad-spectrum cyclic boronate β-lactamase inhibitors featuring an intramolecular prodrug for oral bioavailability	PubMed	Other (outside the scope of this review)
788	4,7-Dichloro benzothien-2-yl sulfonylaminomethyl boronic acid: first boronic acid-derived beta-lactamase inhibitor with class A, C, and D activity	PubMed	Other (outside the scope of this review)
789	Structural study of phenyl boronic acid derivatives as AmpC beta-lactamase inhibitors	PubMed	Other (outside the scope of this review)
790	Optimizing cell permeation of an antibiotic resistance inhibitor for improved efficacy	PubMed	Other (outside the scope of this review)
791	Antimicrobial Activity of Aztreonam-Avibactam and Other β-Lactamase Inhibitor Combinations Tested Against Enterobacterales Isolates from Pediatric Patients from United States Medical Centers (2019-2023)	PubMed	Focused on other β-lactamase inhibitor combinations
792	Structural Comparisons of Cefotaximase (CTX-M-ase) Sub Family 1	PubMed	Other (outside the scope of this review)
793	Treatment of Carbapenem-Resistant Enterobacteriaceae Infections in Children	PubMed	Other (outside the scope of this review)
794	Treatment for carbapenem-resistant Enterobacterales infections: recent advances and future directions	PubMed	Other (outside the scope of this review)
795	Imipenem/Relebactam Plus Aztreonam: First Reported Use in MDR Klebsiella pneumoniae Sternal Infection Complicated by Bacteremia	PubMed	Focused on other β-lactamase inhibitor combinations
796	Alternative and Experimental Therapies of Mycobacterium abscessus Infections	PubMed	Mycobacterium-focused
797	Genomic Characterization of KPC-31 and OXA-181 Klebsiella pneumoniae Resistant to New Generation of β-Lactam/β-Lactamase Inhibitor Combinations	PubMed	Other (outside the scope of this review)
798	Insights Into the Inhibition of MOX-1 β-Lactamase by S02030, a Boronic Acid Transition State Inhibitor	PubMed	Other (outside the scope of this review)
799	Current opinion in management of septic shock due to Gram-negative bacteria	PubMed	Other (outside the scope of this review)
800	Treatment Options for Critically Ill Patients with Infections Caused by Metallo-Beta-Lactamase-Producing Klebsiella pneumoniae	PubMed	Other (outside the scope of this review)

801	Trends of β-Lactamase Occurrence Among Escherichia coli and Klebsiella pneumoniae in United States Hospitals During a 5-Year Period and Activity of Antimicrobial Agents Against Isolates Stratified by β-Lactamase Type	PubMed	In vitro activity or surveillance data
802	Synergistic effects of sulopenem in combination with cefuroxime or durlobactam against Mycobacterium abscessus	PubMed	Mycobacterium-focused
803	Tackling multidrug-resistant Gram-negative infections in children globally: current therapeutic options and perspectives	PubMed	General review or guidance (not boronate-focused)
804	Executive summary of the consensus document of the Spanish Society of Infectious Diseases and Clinical Microbiology (SEIMC) on the diagnosis and antimicrobial treatment of infections due to carbapenem-resistant Gram-negative bacteria	PubMed	Other (outside the scope of this review)
805	The "Old" and the "New" Antibiotics for MDR Gram-Negative Pathogens: For Whom, When, and How	PubMed	Other (outside the scope of this review)
806	Real-world use of novel β-lactams in immunologically vulnerable patients: insights from an Italian retrospective cohort	PubMed	Clinical-outcomes or pharmacoeconomic study
807	Cost-effectiveness analysis of Vaborem in Carbapenem-resistant Enterobacterales (CRE) -Klebsiella pneumoniae infections in Italy	PubMed	Clinical-outcomes or pharmacoeconomic study
808	Clinical efficacy and safety of novel antibiotics for complicated urinary tract infection: A systematic review and meta-analysis of randomized controlled trials	PubMed	General review or guidance (not boronate-focused)
809	Risk Factors for Carbapenem-Resistant Enterobacterales Clinical Treatment Failure	PubMed	Other (outside the scope of this review)
810	Klebsiella pneumoniae Mutants Resistant to Ceftazidime-Avibactam Plus Aztreonam, Imipenem-Relebactam, Meropenem-Vaborbactam, and Cefepime-Taniborbactam	PubMed	Focused on other β-lactamase inhibitor combinations
811	Pharmacokinetics-pharmacodynamics of β-lactamase inhibitors: are we missing the target?	WoS	PK/PD or pharmaceutical-technology study
812	New promising β-lactamase inhibitors for clinical use	WoS	Other (outside the scope of this review)
813	Reviving (3-lactams for Mycobacterium abscessus: the emerging role of durlobactam	WoS	Mycobacterium-focused
814	Novel β-Lactam/β-Lactamase inhibitor combinations vs alternative antibiotics in the treatment of complicated urinary tract infections A meta-analysis of randomized controlled trials	WoS	Other (outside the scope of this review)
815	Broad-spectrum cyclic boronate beta-lactamase inhibitors featuring an	WoS	Other (outside the scope of this review)

	intramolecular prodrug for oral bioavailability.		
816	The road to avibactam: the first clinically useful non-β-lactam working somewhat like a β-lactam	WoS	Other (outside the scope of this review)
817	Efficacy and safety of novel β-lactam/β-lactamase inhibitor combinations for the treatment of complicated urinary tract infections or acute pyelonephritis: A systematic review and meta-analysis	WoS	General review or guidance (not boronate-focused)
818	In vitro activity of cefiderocol in combination with new β-lactam/β-lactamase inhibitor combinations (βL-βLICs) against multidrug resistant KPC-producing Klebsiella pneumoniae	WoS	Focused on cefiderocol or other novel agents
819	In vitro activity of cefiderocol against European Enterobacterales, including isolates resistant to meropenem and recent β-lactam/β-lactamase inhibitor combinations	WoS	Focused on cefiderocol or other novel agents
820	Old and New Beta-Lactamase Inhibitors: Molecular Structure, Mechanism of Action, and Clinical Use	WoS	Structural or chemical-synthesis study
821	In vitro activity of cefiderocolcefiderocol against European Pseudomonas aeruginosa and Acinetobacter spp., including isolates resistant to meropenem and recent β-lactam/β-lactamase inhibitorcombinationsinhibitor combinations	WoS	Focused on cefiderocol or other novel agents
822	In vitroactivity of cefiderocol against European Pseudomonas aeruginosaand Acinetobacterspp., including isolates resistant to meropenem and recent beta-lactam/beta-lactamase inhibitor combinations.	WoS	Focused on cefiderocol or other novel agents
823	VABORBACTAM Rec INN β-Lactamase inhibitor, Antibacterial agent	WoS	Database record (non-research item)
824	Recent advances in the development ofβ-lactamase inhibitors	WoS	Other (outside the scope of this review)
825	Durlobactam, a Broad-Spectrum Serine ß-lactamase Inhibitor, Restores Sulbactam Activity Against Acinetobacter Species	WoS	In vitro activity or surveillance data
826	Global Surveillance of the Activity of Sulbactam Combined With the Novel β-lactamase Inhibitor ETX2514 Against Clinical Isolates of Acinetobacter baumannii From 2014	WoS	Combination-therapy study
827	The β-lactamase Inhibitor Boronic Acid SM23 Inhibits Pseudomonas aeruginosa Biofilm Formation and Virulence Factor Production	WoS	Other (outside the scope of this review)
828	Comment on: An update on cefepime and its future role in combination with novel β-lactamase inhibitors for MDR Enterobacterales and Pseudomonas aeruginosa	WoS	Correction, comment, or reply
829	Safety and Pharmacokinetics (PK) in	WoS	PK/PD or

	Humans of Intravenous ETX2514, a β-lactamase Inhibitor (BLI) which Broadly Inhibits Ambler Class A, C, and D β-lactamases		pharmaceutical-technology study
830	Synthesis of β-Lactamase Inhibitor QPX7728	WoS	Structural or chemical-synthesis study
831	Cefepime/taniborbactam Fixed-dose combination Cephalosporin β-lactam antibiotic/β-lactamase inhibitor Treatment of complicated urinary tract infection	WoS	Other (outside the scope of this review)
832	Transcending the challenge of evolving resistance mechanisms in Pseudomonas aeruginosa through β-lactam-enhancer-mechanism-based cefepime/zidebactam	WoS	Focused on other β-lactamase inhibitor combinations
833	Resistance to aztreonam in combination with non-β-lactam β-lactamase inhibitors due to the layering of mechanisms in Escherichia coli identified following mixed culture selection.	WoS	Combination-therapy study
834	Novel β-lactamase inhibitors: a therapeutic hope against the scourge of multidrug resistance	WoS	Other (outside the scope of this review)
835	Structural study of phenyl boronic acid derivatives as AmpC β-lactamase inhibitors	WoS	Other (outside the scope of this review)
836	Sulbactam Combined With the Novel β-lactamase Inhibitor ETX2514 for the Treatment of Multidrug-Resistant Acinetobacter baumannii Infections	WoS	Combination-therapy study
837	Meropenem/Vaborbactam for Complicated UTIs	WoS	Other (outside the scope of this review)
838	Filling the Gaps: A Review and Update on Beta-Lactamase Inhibitor Combinations	WoS	General review or guidance (not boronate-focused)
839	Evaluating Taniborbactam as an Inhibitor of the SDS-Hydrolyzing Enzyme SdsA1 via Structural and Docking Analysis	WoS	Structural or chemical-synthesis study
840	The emerging concern of IMP variants being resistant to the only IMP-type metallo-beta-lactamase inhibitor, xeruborbactam.	WoS	Other (outside the scope of this review)
841	Aromatic Diboronic Acids as Effective KPC/AmpC Inhibitors	WoS	Other (outside the scope of this review)
842	The β-lactamase Inhibitor Boronic Acid SM23 as a new anti-Pseudomonas aeruginosa Biofilm Compound	WoS	Other (outside the scope of this review)
843	Fragment-guided design of subnanomolar β-lactamase inhibitors active in vivo	WoS	Structural or chemical-synthesis study
844	Fitness and Substrate Specificity Among Serine β-Lactamases: A Study of KPC, SHV, and the AmpC of Pseudomonas aeruginosa	WoS	Other (outside the scope of this review)
845	Chances/Challenges of the Role of New Beta-lactamase Inhibitors Against the Growing Threat of NDM-producing	WoS	Other (outside the scope of this review)

	Escherichia coli With Penicillin-binding Protein 3 Mutations		
846	Scalable Synthesis of β-Lactamase Inhibitor QPX7728 by Sequential Nickel-Catalyzed Boron Insertion into a Benzofuran Substrate and Enantioselective Cyclopropanation of the Resulting Vinylboronate	WoS	Structural or chemical-synthesis study
847	Mechanistic Studies of Antibiotic Resistance: Developing Novel Approaches to Overcome KPC β-Lactamases; a Global Threat	WoS	Other (outside the scope of this review)
848	A method of treating a bacterial infection in a subject in need thereof includes administering to the subject therapeutically effective amts. of at least one β-lactam antibiotic and at least one triazolylmethyl boronic acid β-lactamase inhibitor.	WoS	Patent
849	513. Cefiderocol Retains in vitro Activity Against Enterobacterales Non-Susceptible to β-lactam-β-lactamase Inhibitor Combinations	WoS	Conference abstract without full primary data
850	Stereoselective synthesis and mechanistic studies of boron-containing antibacterials and beta-lactamase inhibitors	WoS	Structural or chemical-synthesis study
851	The Growing Threat of NDM-Producing E. Coli With Penicillin-Binding Potein 3 Mutations in the United States-Is There a Potential Role for Durlobactam?	WoS	Other (outside the scope of this review)
852	Safety, Pharmacokinetics, and DrugDrug Interaction Potential of Intravenous Durlobactam, a b-Lactamase Inhibitor, in Healthy Subjects (vol 64, e00071, 2020)	WoS	PK/PD or pharmaceutical-technology study
853	Sulbactam/Durlobactam (Xacduro) for Acinetobacter Pneumonia	WoS	Other (outside the scope of this review)
854	Identification of novel KPC-2 mutations conferring resistance to β-lactam/β-lactamase inhibitor combinations: implications for genomic surveillance and clinical diagnostics	WoS	In vitro activity or surveillance data
855	Imipenem-Relebactam and Meropenem-Vaborbactam: Two Novel Carbapenem-β-Lactamase Inhibitor Combinations (vol 78, pg 65, 2018)	WoS	Focused on other β-lactamase inhibitor combinations
856	Broth Microdilution Methods Using B-Lactamase Inhibitors for the Identification of Klebsiella pneumoniae Carbapenemases and Metallo-B-Lactamases in Gram-Negative Bacilli	WoS	Other (outside the scope of this review)
857	New β-lactam antibiotics and β-lactamase inhibitors againstmultidrug-resistant Gram-negative bacteria	WoS	Other (outside the scope of this review)
858	Permeation through the Cell Membrane of a Boron-Based beta-Lactamase Inhibitor	WoS	Other (outside the scope of this review)
859	The Triple Combination of Meropenem, Avibactam, and a Metallo-(3-Lactamase	WoS	Other (outside the scope of this review)

	Inhibitor Optimizes Antibacterial Coverage Against Different (3-Lactamase Producers		
860	Perspectives on Metallo-f3-Lactamase Inhibitors: Challenges and Implications of Metallo-f3-Lactamase Variants	WoS	General review or guidance (not boronate-focused)
861	trans-Haloboration of o-Alkynyl Phenols Generates Halogenated Bicyclic- Boronates Relevant to Broad Spectrum β-Lactamase Inhibitors	WoS	Other (outside the scope of this review)
862	Discovery of 3-methacylic acid substituted benzoxaboroles as dual metallo-and serine-(β-lactamase inhibitors	WoS	Other (outside the scope of this review)
863	In vitro pharmacokinetics/pharmacodynamics of the β-lactamase inhibitor, durlobactam, in combination with sulbactam against Acinetobacter baumannii-calcoaceticus complex	WoS	PK/PD or pharmaceutical-technology study
864	Overcoming inhibitor resistance in the SHV beta -lactamase	WoS	Other (outside the scope of this review)
865	Quantitative Insights Into β-Lactamase Inhibitor's Contribution in the Treatment of Carbapenemase-Producing Organisms With β-Lactams	WoS	Other (outside the scope of this review)
866	Boron-Containing Compounds as Antimicrobial Agents to Tackle Drug-Resistant Bacteria	WoS	Other (outside the scope of this review)
867	Diagnosis and Treatment of Carbapenemase-Producing Organisms-an Update	WoS	General review or guidance (not boronate-focused)
868	Innovative beta-lactam/beta-lactamase inhibitor combinations for carbapenem-resistant Gram-negative bacteria	WoS	Other (outside the scope of this review)
869	In Vitro Antimicrobial Activities of Carbapenem-beta Lactamase Inhibitor Combinations Against Carbapenem-resistant Gram-negative Pathogens Isolated from Turkiye	WoS	Other (outside the scope of this review)
870	3RXW: KPC-2 carbapenemase in complex with PSR3-226	WoS	Database record (non-research item)
871	Preserving the Dwindling β-lactams-Based Empiric Therapy Options for Gram-Negative Infections in Challenging Resistance Scenario: Lessons Learned and Way Forward	WoS	Other (outside the scope of this review)
872	Inhibition of class A and C β-lactamases: Challenges and promise	WoS	Other (outside the scope of this review)
873	Sulbactam-Durlobactam for Carbapenem-Resistant Acinetobacter baumannii-calcoaceticus Complex Infections	WoS	Other (outside the scope of this review)
874	Review of Ceftazidime-Avibactam, Meropenem-Vaborbactam, and Imipenem/Cilastatin-Relebactam to TargetKlebsiella pneumoniaeCarbapenemase-Producing Enterobacterales	WoS	General review or guidance (not boronate-focused)

875	Susceptibility Assessment and Review of Novel Beta-Lactam/Beta-Lactamase Inhibitor against Carbapenem-Resistant Enterobacterales	WoS	In vitro activity or surveillance data
876	Carbapenemase inhibitors	WoS	Other (outside the scope of this review)
877	Structure-based optimization of cephalothin-analogue boronic acids as β-lactamase inhibitors	WoS	Structural or chemical-synthesis study
878	Activity of cefiderocol and innovative fi-lactam/ fi-lactamase inhibitor combinations against isogenic strains of Escherichia coli expressing single and double fi-lactamases under high and low permeability conditions	WoS	Focused on cefiderocol or other novel agents
879	Cefepime/enmetazobactam (Exblifep) for complicated urinary tract infections.	WoS	Focused on other β-lactamase inhibitor combinations
880	Recently developed synthetic compounds with anti-infective activity	WoS	Other (outside the scope of this review)
881	New antibiotics in clinical pipeline for treating infections caused by metallo-β-lactamases producing Gram-negative bacteria	WoS	General review or guidance (not boronate-focused)
882	3RXX: KPC-2 carbapenemase in complex with 3-NPBA	WoS	Database record (non-research item)
883	19F NMR Monitoring of Reversible Protein Post-Translational Modifications: Class D β-Lactamase Carbamylation and Inhibition	WoS	Structural or chemical-synthesis study
884	Present and future of resistance in Pseudomonas aeruginosa: implications for treatment	WoS	Other (outside the scope of this review)
885	Inhibition of the Class C β-Lactamase from Acinetobacter spp.: Insights into Effective Inhibitor Design	WoS	Other (outside the scope of this review)
886	218. A Phase 1 Study of the Single-Dose Safety, Tolerability, and Pharmacokinetics of the Beta-lactamase inhibitor Xeruborbactam Administered as the Isobutyryloxymethyl Oral Prodrug to Healthy Adult Subjects	WoS	Conference abstract without full primary data
887	Structure-based enhancement of boronic acid-based inhibitors of AmpC β-lactamase	WoS	Structural or chemical-synthesis study
888	Antimicrobial resistance among imipenem-non-susceptible Escherichia coli and Klebsiella pneumoniae isolates, with an emphasis on novel β-lactam/ β-lactamase inhibitor combinations and tetracycline derivatives: The Taiwan surveillance of antimicrobial resistance program, 2020-2022	WoS	In vitro activity or surveillance data
889	Structure-based design guides the improved efficacy of deacylation transition state analogue inhibitors of TEM-1 β-lactamase	WoS	Structural or chemical-synthesis study
890	In vitro activity of the novel β-	WoS	Combination-therapy

	lactamase inhibitor taniborbactam (VNRX-5133), in combination with cefepime or meropenem, against MDR Gram-negative bacterial isolates from China (vol 75, pg 1850, 2020)		study
891	Binding and Unbinding Pathways of a β-Lactamase Inhibitor	WoS	Other (outside the scope of this review)
892	Carbapenemase Mechanism Testing to Support Treatment, Infection Control, and Public Health The Role of the Clinical Laboratory	WoS	Detection or diagnostic methodology
893	Averting the post-antibiotic era: successful use of meropenem/vaborbactam for carbapenem-resistant Serratia marcescens and Enterobacter aerogenes bacteraemia in a haemodialysis patient	WoS	Other (outside the scope of this review)
894	4E3L: Crystal structure of AmpC beta-lactamase in complex with a 3-chloro-4-tetrazolyl benzene sulfonamide boronic acid inhibitor	WoS	Database record (non-research item)
895	Antifungal agent tavaborole as a potential broad-spectrum serine and metallo-(3-lactamases inhibitor	WoS	Other (outside the scope of this review)
896	Broth Microdilution Method To Detect Extended-Spectrum β-Lactamases and AmpC β-Lactamases in Enterobacteriaceae Isolates by Use of Clavulanic Acid and Boronic Acid as Inhibitors	WoS	Detection or diagnostic methodology
897	Molecular Docking and Molecular Dynamics Simulations to Introduce New Inhibitors for Ambler Class C of β-lactamase enzymes	WoS	Structural or chemical-synthesis study
898	1710. In Vitro Activity of Cefepime-Taniborbactam and Comparators Against a Global Collection of Carbapenem-Resistant Enterobacterales and Carbapenem-Resistant Pseudomonas aeruginosa With and Without Carbapenemases	WoS	Conference abstract without full primary data
899	P-1081. Activity of Aztreonam-Avibactam against Enterobacterales Isolated from Patients with Intra-Abbdominal Infection from United States Medical Centers (2019-2023)	WoS	Conference abstract without full primary data
900	X-ray crystal structure of the extended-spectrum class C β-lactamase, ADC-7, in apo form and in complex with a boronic acid transition state analog	WoS	Structural or chemical-synthesis study
901	Cyclic boronic acid beta-lactamase inhibitors	WoS	Other (outside the scope of this review)
902	New-generation antibiotics and nonantibiotic strategies against carbapenemase-producing Enterobacterales: More focus on metallo- β-lactamase producers	WoS	Other (outside the scope of this review)
903	Carbapenem-Resistant Enterobacteriaceae in Solid Organ	WoS	Other (outside the scope of this review)

	Transplantation: Management Principles		
904	614. Pharmacodynamics (PD) of the Beta-Lactamase Inhibitor Xeruborbactam When Administered as the Oral Prodrug in Combination with Ceftibuten	WoS	Conference abstract without full primary data
905	Exploring Additional Dimensions of Complexity in Inhibitor Design for Serine β-Lactamases: Mechanistic and Intra- and Inter-molecular Chemistry Approaches	WoS	Other (outside the scope of this review)
906	Synthesis and Mechanistic Analyses of Boron-Containing β-Lactamase Inhibitors	WoS	Structural or chemical-synthesis study
907	Promising Future of Novel Beta-Lactam Antibiotics Against Bacterial Resistance	WoS	Other (outside the scope of this review)
908	615. Pharmacodynamics (PD) of the Beta-Lactamase Inhibitor Xeruborbactam When Administered in Combination with Meropenem	WoS	Conference abstract without full primary data
909	New Antibiotics Against Multidrug-Resistant Gram-Negative Bacteria in Liver Transplantation: Clinical Perspectives, Toxicity, and PK/PD Properties	WoS	General review or guidance (not boronate-focused)
910	Activity of aztreonam in combination with novel ,B-lactamase inhibitors against metallo- ,B-lactamase-producing Enterobacterales from Spain	WoS	Combination-therapy study
911	4E3K: Crystal structure of AmpC beta-lactamase in complex with a designed 4-tetrazolyl pyridine sulfonamide boronic acid inhibitor	WoS	Database record (non-research item)
912	4E3I: Crystal structure of AmpC beta-lactamase in complex with a designed 3-carboxyl benzyl sulfonamide boronic acid inhibitor	WoS	Database record (non-research item)
913	1621. Novel Beta-lactam Beta-lactamase Inhibitors Against Alternative Antibiotics for the Treatment of Complicated Urinary Tract Infections and Pyelonephritis Caused by Carbapenem-resistant Enterobacterales	WoS	Conference abstract without full primary data
914	Present and Future of Carbapenem-resistant Enterobacteriaceae (CRE) Infections	WoS	Other (outside the scope of this review)
915	Development of a Continuous Flow Process for a Matteson Reaction: From Lab Scale to Full-Scale Production of a Pharmaceutical Intermediate	WoS	Other (outside the scope of this review)
916	Detection of extended-spectrum β-lactamases by using boronic acid as an AmpC β-lactamase inhibitor in clinical isolates of Klebsiella spp. and Escherichia coli	WoS	Detection or diagnostic methodology
917	In silico identification and experimental validation of hits active against KPC-2 β-lactamase	WoS	Other (outside the scope of this review)

918	Activity of biapenem (RPX2003) combined with the boronate -lactamase inhibitor RPX7009 against carbapenem-resistant Enterobacteriaceae	WoS	Combination-therapy study
919	Design, synthesis and characterization of peptidyl boronate analogues as effective antimicrobial agents	WoS	Structural or chemical-synthesis study
920	727. Potency of the β-Lactamase Inhibitor QPX7728 Is Minimally Affected by KPC Mutations that Reduce Potency of Ceftazidime–Avibactam	WoS	Conference abstract without full primary data
921	β-Lactamase Inhibitors in Overcoming Bacterial Antibiotic Resistance	WoS	Other (outside the scope of this review)
922	China's new national action plan to combat antimicrobial resistance (2022-25)	WoS	Other (outside the scope of this review)
923	4E3J: Crystal structure of AmpC beta-lactamase in complex with a designed 4-tetrazolyl benzene sulfonamide boronic acid inhibitor	WoS	Database record (non-research item)
924	2125. Activity of Novel β-Lactam/β-Lactamase Inhibitor Combinations against Serine-Carbapenemase producing Carbapenem-Resistant Pseudomonas aeruginosa	WoS	Conference abstract without full primary data
925	Perspectives on the use of ceftolozane/tazobactam: a review of clinical trial data and real-world evidence	WoS	Focused on other β-lactamase inhibitor combinations
926	In vitro Synergistic and Bactericidal Effects of Aztreonam in Combination with Ceftazidime/ Avibactam, Meropenem/Vaborbactam and Imipenem/ Relebactam Against Dual-Carbapenemase-Producing Enterobacterales	WoS	Focused on other β-lactamase inhibitor combinations
927	P-775. Unlocking Synergistic Potency: Dual Action of β-Lactam and β-Lactamase Inhibitor in Combating Mycobacterium abscessus through β-Lactamase Inhibition and Multi-Target Receptor Inactivation	WoS	Conference abstract without full primary data
928	In vitro activity and resistance mechanisms of sulbactam/ durlobactam against Acinetobacter baumannii clinical isolates in China (2019-2020)	WoS	In vitro activity or surveillance data
929	New Antibiotics for the Treatment of multidrug-resistant gram-negative Bacteria	WoS	Other (outside the scope of this review)
930	The role of PK/PD-based strategies to preserve new molecules against multi-drug resistant gram-negative strains	WoS	Other (outside the scope of this review)
931	4E3O: Crystal structure of AmpC beta-lactamase in complex with a small chloromethyl sulfonamide boronic acid inhibitor	WoS	Database record (non-research item)
932	Clinical trials that could change the management of severe multidrug-	WoS	Other (outside the scope of this review)

	resistant Gram-negative infections		
933	The Novel β-Lactamase Inhibitor, ETX-2514, in Combination with Sulbactam Effectively Inhibits Acinetobacter baumannii	WoS	Combination-therapy study
934	RETRACTED: Phenotypes, genotypes and breakpoints: an assessment of β-lactam/β-lactamase inhibitor combinations against OXA-48 (Retracted article. See JAN, 2023)	WoS	Detection or diagnostic methodology
935	2784. Rates of Susceptibility and Heteroresistance to Novel Antibiotic Combinations in Carbapenem-Resistant Enterobacterales Isolates - Emory Healthcare, 2016-2021	WoS	Conference abstract without full primary data
936	Genome-driven identification of patient and bacterial drivers of resistance to novel beta-lactam/beta-lactamase inhibitor combination therapies	WoS	Other (outside the scope of this review)
937	3O86: Crystal structure of AmpC beta-lactamase in complex with a sulfonamide boronic acid inhibitor	WoS	Database record (non-research item)
938	P-1082. Antimicrobial Susceptibility of Enterobacterales Causing Infection in the Elderly: Focus on Aztreonam-avibactam and Recently Approved β-Lactamase Inhibitor Combinations	WoS	Conference abstract without full primary data
939	Probing active site chemistry in SHV β-lactamase variants at ambler position 244 -: Understanding unique properties of inhibitor resistance	WoS	Other (outside the scope of this review)
940	Differentiated treatment of multi-resistant Gram-negative pathogens in intensive care units	WoS	Other (outside the scope of this review)
941	Structure and dynamics of Burkholderia pseudomallei OXA-57, a distinctive low-efficiency class D β-lactamase with carbapenem-hydrolyzing activity	WoS	Structural or chemical-synthesis study
942	166. Activity of a Novel β-lactamase Inhibitor QPX7728 Combined With β-lactams Against st258 klebsiella Pneumoniae and st131 escherchia Coli Isolates Producing β-lactamases	WoS	Conference abstract without full primary data
943	709. In Vitro Antibacterial Activity and In Vivo Efficacy of Sulbactam–Durlobactam (ETX2514SUL) Against Pathogenic Burkholderia Species	WoS	Conference abstract without full primary data
944	4E3M: Crystal structure of AmpC beta-lactamase in complex with a 2-chloro-4-tetrazolyl benzene sulfonamide boronic acid inhibitor	WoS	Database record (non-research item)
945	4E3N: Crystal structure of AmpC beta-lactamase in complex with a 2-trifluoromethyl-4-tetrazolyl benzene sulfonamide boronic acid inhibitor	WoS	Database record (non-research item)
946	1395. Defining the Magnitude of AUC:MIC Driver for Efficacy of the β-Lactamase Inhibitor VNRX-5133 When Combined with Cefepime Against KPC-	WoS	Conference abstract without full primary data

	and VIM/NDM-Producing Enterobacteriaceae and P. aeruginosa		
947	Highly Sensitive In Vivo Imaging of Bacterial Infections with a Hydrophilicity-Switching, Self-Immobilizing, Near-Infrared Fluorogenic β-Lactamase Probe Enriched within Bacteria	WoS	Other (outside the scope of this review)
948	NCY-I beta-lactamase activity correlates with antimicrobial susceptibility of a clinical strain of Nocardia cyriacigeorgica	WoS	In vitro activity or surveillance data
949	Combinatorial Therapeutic Strategies to Combat Multidrug-Resistant Acinetobacter baumannii	WoS	Other (outside the scope of this review)
950	Penam Sulfones and β-Lactamase Inhibition: SA2-13 and the Importance of the C2 Side Chain Length and Composition	WoS	Other (outside the scope of this review)
951	1360. Antimicrobial Activity of Cefepime in Combination with VNRX-5133 Against a Global Collection of Enterobacteriaceae Including Resistant Phenotypes	WoS	Conference abstract without full primary data
952	216. A Phase 1 Study of the Safety, Tolerability, and Pharmacokinetics of Multiple Doses of the Beta-lactamase inhibitor Xeruborbactam Alone and in Combination Meropenem in Healthy Adult Subjects	WoS	Conference abstract without full primary data
953	1642. A Novel β-lactamase Inhibitor (Durlobactam, DUR) and β-Lactams Enhance Susceptibility Against Multidrug-Resistant (MDR) Mycobacterium abscessus (Mab)	WoS	Conference abstract without full primary data
954	3O87: Crystal structure of AmpC beta-lactamase in complex with a sulfonamide boronic acid inhibitor	WoS	Database record (non-research item)
955	3O88: Crystal structure of AmpC beta-lactamase in complex with a sulfonamide boronic acid inhibitor	WoS	Database record (non-research item)
956	P-1084. Antimicrobial Activity of Aztreonam-Avibactam and Other β-Lactamase Inhibitor Combinations Tested against Enterobacterales Isolates from Pediatric Patients from United States Medical Centers (2019–2023)	WoS	Conference abstract without full primary data
957	Editorial: Structural and Biochemical Aspects of the Interaction of β-Lactamases With State-of-the-Art Inhibitors	WoS	Other (outside the scope of this review)
958	Design and synthesis of metallo-β-lactamase inhibitors	WoS	Structural or chemical-synthesis study
959	The complexed structure and antimicrobial activity of a non-β-lactam inhibitor of AmpC β-lactamase	WoS	In vitro activity or surveillance data
960	1949. Activity of Aztreonam-Avibactam against Enterobacterales Resistant to Recently Approved Beta-Lactamase	WoS	Conference abstract without full primary data

	Inhibitor Combinations Collected Worldwide (ex-US; 2020–2022)		
961	700. Identification and Whole-Genome Sequencing (WGS) of Meropenem-Vaborbactam (MV) Resistant Klebsiella pneumoniae (MVRKP) Among Patients Without Prior Exposure to MV: Collateral Damage	WoS	Conference abstract without full primary data
962	Novel Insights into the Mode of Inhibition of Class A SHV-1 β-Lactamases Revealed by Boronic Acid Transition State Inhibitors	WoS	Other (outside the scope of this review)
963	Recent Advancements in the Diversification and Applications of Boron-Containing Compounds in Medicinal Chemistry	WoS	Other (outside the scope of this review)
964	656. Comparative Activity of Newer β-Lactam/β-Lactamase Inhibitor Combinations against Pseudomonas aeruginosa isolates from United States Medical Centers (2020-2021)	WoS	Conference abstract without full primary data
965	Meropenem/Vaborbactam, the First Carbapenem/-Lactamase Inhibitor Combination	WoS	Other (outside the scope of this review)
966	Antimicrobial and KPC/AmpC inhibitory activity of functionalized benzosiloxaboroles	WoS	In vitro activity or surveillance data
967	Oral Metallo-Beta-Lactamase Inhibitors: Exploiting Reaction Mechanisms	WoS	Other (outside the scope of this review)
968	Comparative activity of newer ?-lactam/?-lactamase inhibitor combinations against Pseudomonas aeruginosa isolates from US medical centres (2020-2021)	WoS	In vitro activity or surveillance data
969	Genomic and molecular characterisation of a Klebsiella pneumoniae clinical isolate resistant to meropenem-vaborbactam, imipenem-relebactam, and ceftazidime-avibactam	WoS	Focused on other β-lactamase inhibitor combinations
970	The safety and efficacy of relebactam/imipenem/cilastatin in Japanese patients with complicated intra-abdominal infection or complicated urinary tract infection: A multicenter, open-label, noncomparative phase 3 study	WoS	Other (outside the scope of this review)
971	DETECTION OF AMPC BETA LACTAMASES IN CEFOXITIN-RESISTANT GRAM-NEGATIVE CLINICAL ISOLATES USING PHENYLBORONIC ACID	WoS	Detection or diagnostic methodology
972	Evaluation of Ceftolozane/Tazobactam, Ceftazidime/Avibactam and Meropenem/Vaborbactam Against Multidrug-Resistant (MDR) Pseudomonas	WoS	Focused on other β-lactamase inhibitor combinations
973	681. In vitro Activity of the β-Lactamase Inhibitor QPX7728 in Combination with Several β-Lactams	WoS	Conference abstract without full primary data

	Against Acinetobacter baumannii (AB) and Pseudomonas aeruginosa (PSA)		
974	Structural Determinants for Reversible β-Lactamase Inhibition	WoS	Other (outside the scope of this review)
975	STABILITY INDICATING RP-HPLC METHOD DEVELOPMENT AND VALIDATION OF MEROPENEM AND VABORBACTAM IN PHARMACEUTICAL DOSAGE FORM	WoS	PK/PD or pharmaceutical-technology study
976	2598. Activity of Aztreonam-Avibactam and Other β-Lactamase Inhibitor Combinations against Gram-negative Bacteria Isolated from Patients Hospitalized with Pneumonia in US Medical Centers (2020–2022)	WoS	Conference abstract without full primary data
977	Alternative and Experimental Therapies ofMycobacterium abscessusInfections	WoS	Mycobacterium-focused
978	610. Meropenem-vaborbactam (MV) In Vitro Activity Against Carbapenem-Resistant Klebsiella pneumoniae (CRKP) Isolates with Outer Membrane Porin Gene Mutations	WoS	Conference abstract without full primary data
979	1254. In vitro activity of sulbactam-durlobactam against recent global clinical Acinetobacter baumannii-calcoaceticus complex isolates	WoS	Conference abstract without full primary data
980	1676. PK, Safety and Tolerability of VABOMERE® (meropenem-vaborbactam) in Infants, Children and Adolescents	WoS	Conference abstract without full primary data
981	P-1521. Susceptibility of Carbapenem-Resistant Enterobacterales (CRE) and Carbapenem-Resistant Pseudomonas aeruginosa (CRPA) With and Without Carbapenemases to Cefepime-Taniborbactam and Comparators: GEARS Antimicrobial Surveillance Program, United States, 2018-2022	WoS	Conference abstract without full primary data
982	1693. Meropenem-Xeruborbactam: In Vitro Potency against Gram-Negative Bacteria in Comparison with Marketed and Investigational Beta-lactam (BL)/Beta-lactamase Inhibitor (BLI) Combinations	WoS	Conference abstract without full primary data
983	700. Identification and Whole-Genome Sequencing (WGS) of Meropenem-Vaborbactam (MV) Resistant (MVRKP) Among Patients Without Prior Exposure to MV: Collateral Damage.	WoS	Conference abstract without full primary data
984	Multicenter Clinical Evaluation of Etest Meropenem-Vaborbactam (bioMerieux) for Susceptibility Testing of Enterobacterales (Enterobacteriaceae) and Pseudomonas aeruginosa	WoS	In vitro activity or surveillance data
985	In vitro evaluation of sulbactam-durlobactam as a therapeutic alternative for multidrug-resistant Acinetobacter baumannii infections:	WoS	Other (outside the scope of this review)

	insights from a tertiary care hospital		
986	2150. In Vitro Activity of Cefepime-Taniborbactam and Comparators Against Genotypically Characterized Carbapenem-Resistant Enterobacterales (CRE) and Carbapenem-Resistant Pseudomonas aeruginosa (CRPA) from the United States, 2018-2021	WoS	Conference abstract without full primary data
987	1692. Ceftibuten-Xeruborbactam: In vitro Potency against Enterobacterales in Comparison with Other Oral Beta-lactams (BL) and Beta-lactamase Inhibitor (BLI) Combinations	WoS	Conference abstract without full primary data
988	Novel Pathways and Targets for Combating Antimicrobial Resistance	WoS	Other (outside the scope of this review)
989	Drug Repurposing for Mycobacterium Abscessus: Investigating Drug Combinations	WoS	Mycobacterium-focused
990	2122. In Vitro Activity of Cefepime-Taniborbactam Against Clinically Significant Gram-Negative Bacteria Isolated from Patients with Cancer	WoS	Conference abstract without full primary data
991	Activity of cefiderocol and innovative β-lactam/ β-lactamase inhibitor combinations against isogenic strains of Escherichia coli expressing single and double β-lactamases under high and low permeability conditions (vol 63, 107150, 2024)	WoS	Focused on cefiderocol or other novel agents
992	1253. Antimicrobial Activity of Cefepime in Combination with Taniborbactam Against Clinical Isolates of Enterobacterales from 2018-2020 Global Surveillance	WoS	Conference abstract without full primary data
993	1619. Meta-analysis of Randomized Control Trials Evaluating New Beta-Lactamase Combination Antibiotics	WoS	Conference abstract without full primary data
994	656. Sulbactam-Durlobactam MIC Determination: Comparative Evaluation of the New ETEST® SUD to the CLSI 2021 Broth Microdilution Method	WoS	Conference abstract without full primary data
995	203. Activity of Cefepime in Combination with Taniborbactam (formerly VNRX-5133) Against Pseudomonas aeruginosa from a Global 2018-2020 Surveillance Collection	WoS	Conference abstract without full primary data
996	521. Comparative In Vitro Activity of Meropenem/Vaborbactam and Meropenem Against a Collection of Real-World Clinical Isolates of Pseudomonas aeruginosa	WoS	Conference abstract without full primary data
997	Indian Society of Critical Care Medicine Guidelines on Evidence-based Recommendations for the Treatment of Carbapenem-resistant Enterobacterales, Carbapenem-resistant Acinetobacter baumannii, and Carbapenem-resistant Pseudomonas	WoS	General review or guidance (not boronate-focused)

	aeruginosa in Critically Ill Patients: A GRADE guided Approach		
998	2515. Evaluation of the Equivalency of the Oxoid Sulbactam-Durlobactam (10/10µg) Antimicrobial Disc to the CLSI M07 Broth Microdilution Frozen Reference Method	WoS	Conference abstract without full primary data
999	1672. Antimicrobial Activity of Cefepime-Taniborbactam and Comparators Against Clinical Isolates from ICU and Non-ICU Patients; 2018-2020 Global Surveillance	WoS	Conference abstract without full primary data
1000	694. In vitro Antibacterial Activity of Sulbactam–Durlobactam (ETX2514SUL) Against 121 Recent Acinetobacter baumannii Isolated from Patients in India	WoS	Conference abstract without full primary data
1001	Development of an Antibiotic Resistance Breaker to Resensitize Drug-Resistant Staphylococcus aureus: In Silico and In Vitro Approach	WoS	Other (outside the scope of this review)
1002	1293. Sulbactam-durlobactam is active against recent, multi-drug resistant Acinetobacter baumannii clinical isolates from the Middle East	WoS	Conference abstract without full primary data
1003	1370. Cefepime/VNRX-5133 Broad-Spectrum Activity Is Maintained Against Emerging KPC- and PDC-Variants in Multidrug-Resistant K. pneumoniae and P. aeruginosa	WoS	Conference abstract without full primary data
1004	Reversible inhibitors of penicillinases.	WoS	Other (outside the scope of this review)
1005	1390. Durlobactam, a Diazabicyclooctane (DBO) β-lactamase Inhibitor (BLI), Inhibits BlaC and Peptidoglycan (PG) Transpeptidases of Mycobacterium tuberculosis (Mtb): A Novel Approach to Therapeutics for Tuberculosis (TB)?	WoS	Conference abstract without full primary data
1006	The beta-lactamases of rapidly growing mycobacteria	WoS	Other (outside the scope of this review)
1007	2178. Resistance to Cefepime/Taniborbactam in NDM-1 - Are We Losing the Battle against MDROs?	WoS	Conference abstract without full primary data
1008	Using Molecular Diagnostics to Develop Therapeutic Strategies for Carbapenem-Resistant Gram-Negative Infections	WoS	Other (outside the scope of this review)
1009	P-1218. Assessment of Cefepime-Taniborbactam (FEP-TAN) Transmembrane Clearance (CLTM) in an Ex vivo Continuous Renal Replacement Therapy (CRRT) Model	WoS	Conference abstract without full primary data
1010	1263. Assessment of Cefepime (FEP)-Taniborbactam (TAN) Human Exposures to Suppress the Emergence of Resistance among Serine (SBL)- and Metallo-β-Lactamase (MBL)-Producing Gram-Negative Bacteria (GNB) in a	WoS	Conference abstract without full primary data

	Hollow Fiber Infection Model (HFIM)		
1011	Reassessment of the cooperativity between efflux system MexAB-OprM and cephalosporinase AmpC in the resistance of Pseudomonas aeruginosa to β-lactams	WoS	Other (outside the scope of this review)
1012	Will Snake Venoms Help Overcome Antimicrobial Resistance?	WoS	Other (outside the scope of this review)
1013	514. The Impact of Xeruborbactam on in vitro Activity of Cefiderocol against a Panel of Acinetobacter baumannii Enriched in Isolates with Reduced Cefiderocol Susceptibility	WoS	Conference abstract without full primary data
1014	P-1514. Cefepime-taniborbactam Exhibits Limited Cross-resistance with Ceftazidime-avibactam and Ceftolozane-tazobactam against Carbapenem-nonsusceptible Enterobacterales and Multidrug-resistant Pseudomonas aeruginosa from the United States 2018-2022	WoS	Conference abstract without full primary data
1015	Exploring Covalent Docking Mechanisms of Boron-Based Inhibitors to Class A, C and D β-Lactamases Using Time-dependent Hybrid QM/MM Simulations	WoS	Structural or chemical-synthesis study
1016	P-650. In vitro Activity of Cefiderocol and Comparator Agents Against Global Enterobacterales Isolates From Hospitalized Patients with Pneumonia Collected as Part of the SENTRY Surveillance Program (2020-2022)	WoS	Conference abstract without full primary data
1017	142. Evaluation of Meropenem-Vaborbactam Susceptibility and Underlying Resistance Mechanisms among Clinical KPC-producing Klebsiella pneumoniae	WoS	Conference abstract without full primary data
1018	P-1080. Aztreonam-Avibactam Activity Against Gram-negative Bacteria Isolated from Patients with Pneumonia from Europe, Asia, and Latin America (2021–2023)	WoS	Conference abstract without full primary data
1019	1737. Xeruborbactam (QPX) Potentiates the Activity of β-Lactam (BL) Antibiotics Against a Diverse Group of Highly Drug-Resistant Enterobacterales (ENT)	WoS	Conference abstract without full primary data
1020	Structure, function, and inhibition along the reaction coordinate of CTX-M β-lactamases	WoS	Structural or chemical-synthesis study
1021	2185. In vitro activity of aztreonam-avibactam (ATM-AVI), cefiderocol (FDC), and cefepime-taniborbactam (FEP-TAN) against multi-species, NDM-producing Enterobacterales causing a local outbreak	WoS	Conference abstract without full primary data
1022	1055. ARGONAUT-IV: Susceptibility of Carbapenem-resistant Klebsiellae to Ceftibuten/VNRX-5236	WoS	Conference abstract without full primary data
1023	P-1360. Activity of Cefiderocol against	WoS	Conference abstract

	Carbapenem Non-susceptible Enterobacterales, Including Molecularly Characterized Multidrug-resistant Clinical Isolates, Causing Infections in United States Hospitals (2020–2023)		without full primary data
1024	Inhibition of Klebsiella β-Lactamases (SHV-1 and KPC-2) by Avibactam: A Structural Study	WoS	Other (outside the scope of this review)
1025	644. Activity of Meropenem-Vaborbactam and Comparators Against Isolates from Patients in Hematology/Oncology and Transplant Units in the United States	WoS	Conference abstract without full primary data
1026	Introducing CRAFT: The Center for Research and Advancement in Fragments and molecular Targets	WoS	Structural or chemical-synthesis study
1027	Multidrug Resistant Pseudomonas aeruginosa in Clinical Settings: A Review of Resistance Mechanisms and Treatment Strategies	WoS	General review or guidance (not boronate-focused)
1028	An Update on Advancements in Treatment Options for Managing Klebsiella pneumoniae Infections	WoS	General review or guidance (not boronate-focused)
1029	2129. Antimicrobial Activity of Cefepime in Combination with Taniborbactam Against Resistant Clinical Isolates from the United States 2018-2021	WoS	Conference abstract without full primary data
1030	Global strategies to fight carbapenem-resistant Acinetobacter baumannii (CRAB) infections	WoS	Other (outside the scope of this review)
1031	731. CERTAIN-1: A Phase 3 Study of Cefepime-Taniborbactam Efficacy and Safety in the Treatment of Complicated Urinary Tract Infections (cUTI), including Acute Pyelonephritis (AP)	WoS	Conference abstract without full primary data
1032	Antimicrobial Treatment Options for Difficult-to-Treat Resistant Gram-Negative Bacteria Causing Cystitis, Pyelonephritis, and Prostatitis: A Narrative Review	WoS	General review or guidance (not boronate-focused)
1033	Ceftazidime/Avibactam, Meropenem/Vaborbactam, or Both? Clinical and Formulary Considerations	WoS	Other (outside the scope of this review)
1034	2172. Activity of Cefiderocol and Comparator Agents Against Molecularly Characterized Multidrug-resistant Enterobacterales Clinical Isolates from United States Hospitals (2020–2022)	WoS	Conference abstract without full primary data
1035	Amino Acid Based Antimicrobial Agents - Synthesis and Properties	WoS	Structural or chemical-synthesis study
1036	1593. Antimicrobial Activity of Ceftazidime-Avibactam and Comparator Agents Against OXA-48 β-lactamase–Producing Enterobacterales Collected in International Medical Centers, Including the United States, in 2017–2018	WoS	Conference abstract without full primary data

1037	Structure-Based Development of (1-(3'-Mercaptopropanamido)methyl)boronic Acid Derived Broad Spectrum, Dual-Action Inhibitors of Metallo- and Serine-β-lactamases	WoS	Structural or chemical-synthesis study
1038	Use of novel boronic acid transition state inhibitors to probe substrate affinity in SHV-type extended-spectrum β-lactamases	WoS	Other (outside the scope of this review)
1039	732. Microbiologic and clinical outcome concordance in the global phase 3 ATTACK trial: Sulbactam-durlobactam (SUL-DUR) versus colistin therapy in patients with Acinetobacter baumannii-calcoaceticus complex (ABC) infections	WoS	Conference abstract without full primary data
1040	675. Sulbactam-durlobactam (SUL-DUR) versus colistin therapy in patients with Acinetobacter baumannii-calcoaceticus complex (ABC) infections: A detailed safety review from the pivotal phase 3, global, randomized, active-controlled trial (ATTACK)	WoS	Conference abstract without full primary data
1041	133. ARGONAUT-III: Susceptibility of Carbapenem-resistant Klebsiellae to Cefepime-Taniborbactam	WoS	Conference abstract without full primary data
1042	511. Activity of Sulbactam-durlobactam and Standard-of-Care Antibiotics against Acinetobacter baumannii-calcoaceticus complex isolates acquired from Hospitalized Patients in the United States (2023 – 2024)	WoS	Conference abstract without full primary data
1043	P-1241. Cefepime Pharmacodynamics Against Pseudomonas aeruginosa Evaluated in a Chemostat Infection Model: Do Generalized Cephalosporin Targets Translate?	WoS	Conference abstract without full primary data
1044	Newly developed antibiotics against multidrug-resistant and carbapenem-resistant Gram-negative bacteria: action and resistance mechanisms	WoS	Other (outside the scope of this review)
1045	THE EUROPEAN MEDICINES AGENCY APPROVED THE NEW ANTIBACTERIAL DRUGS-RESPONSE TO THE 2017 WHO REPORT ON THE GLOBAL PROBLEM OF MULTI-DRUG RESISTANCE	WoS	Other (outside the scope of this review)
1046	1734. Susceptibility of Cefiderocol between US Census Regions against Gram-Negative Organisms collected from the SENTRY Surveillance Program: 2020-2021	WoS	Conference abstract without full primary data
1047	2802. Frequency, Antimicrobial Susceptibility, and Molecular Characterization of Carbapenem-Resistant Enterobacterales Stratified by United States Census Divisions: Results from the INFORM Program (2018-2022)	WoS	Conference abstract without full primary data

1048	642. In Vitro Activity of Cefiderocol and Comparator Agents Against Molecularly Characterized Enterobacterales Clinical Isolates Causing Infection in United States Hospitals (2020-2021)	WoS	Conference abstract without full primary data
1049	Analyses of Structure and Functional Dynamics of Penicillin-Binding Proteins in Staphylococci	WoS	Structural or chemical-synthesis study
1050	Practical methods using boronic acid compounds for identification of class C β-lactamase-producing Klebsiella pneumoniae and Escherichia coli	WoS	Other (outside the scope of this review)
1051	Infections by multidrug- resistant Gram-negative Bacteria: What's new in our arsenal and what's in the pipeline?	WoS	General review or guidance (not boronate-focused)
1052	2138. In Vitro Susceptibility of Recent Clinical Isolates of P. aeruginosa and Enterobacterales to Imipenem or Meropenem Alone or in Combination with Sulbactam-Durlobactam	WoS	Conference abstract without full primary data
1053	1063. ARGONAUT-V: Susceptibility of Multidrug-Resistant (MDR) Pseudomonas aeruginosa to Cefepime-Taniborbactam	WoS	Conference abstract without full primary data
1054	The Synthesis of Boronic Acid Carbapenem Analogues to Serve as β-Lactamase Inhibitors	WoS	Structural or chemical-synthesis study
1055	1053. The β-Lactamase Inhibitor QPX7728 Restores the Activity of β-Lactam Agents against Contemporary Extended-Spectrum β-lactamase (ESBL)-Producing and Carbapenem-Resistant Enterobacterales (CRE) Isolates, Including Isolates Producing Metallo-β-lactamases	WoS	Conference abstract without full primary data
1056	2146. Antimicrobial Activity of Aztreonam-avibactam, Ceftazidime-avibactam, and Meropenem-vaborbactam against Enterobacterales causing Bloodstream Infection in US Medical Centers (2020–2022)	WoS	Conference abstract without full primary data
1057	1688. Aztreonam-Avibactam Activity against a Large Collection of Carbapenem-Resistant Enterobacterales (CRE) collected in Hospitals from Europe, Asia, and Latin America (2019-2021)	WoS	Conference abstract without full primary data
1058	865. Same Shift Susceptibility Testing of Cefepime-Taniborbactam and Cefiderocol Using The Selux Next-Generation Phenotyping System	WoS	Conference abstract without full primary data
1059	Activity of Meropenem-Vaborbactam against Pseudomonas aeruginosa and Acinetobacter baumannii in a Neutropenic Mouse Thigh Infection Model	WoS	In vitro activity or surveillance data
1060	Therapeutic options for difficult-to-treat Acinetobacter baumanniiinfections: a 2020	WoS	General review or guidance (not boronate-focused)

	perspective		
1061	Aza-boronic acids as non-β-lactam inhibitors of AmpC-β-lactamase	WoS	Other (outside the scope of this review)
1062	P-1499. Patient Characteristics and Clinical Outcomes Associated with Meropenem/Vaborbactam Treatment in Carbapenem-Resistant Enterobacterales Pneumonia	WoS	Conference abstract without full primary data
1063	1686. Antimicrobial Activity of Aztreonam-avibactam, Ceftazidime-Avibactam, and Comparator Agents against Pseudomonas aeruginosa from Cystic Fibrosis Patients	WoS	Conference abstract without full primary data
1064	1736. Xeruborbactam (QPX) Potentiates the Activity of Multiple β-Lactams Against Highly Resistant Pseudomonas aeruginosa to a Greater Degree than Other β-Lactamase Inhibitors	WoS	Conference abstract without full primary data
1065	2513. CERTAIN-1 Subgroup Analysis: A Phase 3 Study of Cefepime-Taniborbactam Efficacy in the Treatment of Complicated Urinary Tract Infections (cUTI)	WoS	Conference abstract without full primary data
1066	Structure Based Inhibitor Discovery Targeting Multiple β-Lactamases Involved in Antibiotic Resistance	WoS	Structural or chemical-synthesis study
1067	2112. In vitro Activity of Cefiderocol and Comparator Agents Against Enterobacterales From United States Hospitals Stratified by Infection Type (2020-2022)	WoS	Conference abstract without full primary data
1068	2761. Activity of Cefiderocol and Comparator Agents Against Achromobacter and Burkholderia Isolates, Collected During 2020-2022 as Part of SENTRY Antimicrobial Surveillance Program	WoS	Conference abstract without full primary data
1069	1310. Antimicrobial Activity of Ceftazidime-Avibactam and Other New β-Lactamase Inhibitor Combinations Tested against Bacterial Isolates from Pediatric Patients from US Medical Centers (2020-2021)	WoS	Conference abstract without full primary data
1070	2755. Activity of Aztreonam-avibactam and Carbapenem-resistant Enterobacterales (CRE) Isolates Collected in a Six-year Period (2017–2022)	WoS	Conference abstract without full primary data
1071	Drug Regimens of Novel Antibiotics in Critically Ill Patients with Varying Renal Functions: A Rapid Review	WoS	General review or guidance (not boronate-focused)
1072	Therapies in preclinical and in early clinical development for the treatment of urinary tract infections: from pathogens to therapies	WoS	Other (outside the scope of this review)
1073	Carbapenem-resistant Enterobacterales susceptibility patterns to new antimicrobials: A single-center analysis	WoS	In vitro activity or surveillance data

1074	2517. Activity of Sulbactam-durlobactam, Antibacterial Combinations, and Comparators Against a Challenge Set of 66 Acinetobacter baumannii-calcoaceticus Species Complex Isolates	WoS	Conference abstract without full primary data
1075	P-1110. The Impact of Xeruborbactam on in vitro Activity of Cefiderocol against a Panel of Enterobacterales Enriched with Isolates with Reduced Cefiderocol Susceptibility	WoS	Conference abstract without full primary data
1076	Antibiotics for multidrug-resistant gram-negative bacteria	WoS	Other (outside the scope of this review)
1077	1286. Taniborbactam Inhibits Cefepime-Hydrolyzing Variants of Pseudomonas-derived Cephalosporinase (PDC)	WoS	Conference abstract without full primary data
1078	Investigational drugs for the treatment of infections caused by multidrug-resistant Gram-negative bacteria	WoS	Other (outside the scope of this review)
1079	What to Do with the New Antibiotics?	WoS	Other (outside the scope of this review)
1080	2790. Activity of Cefiderocol and Comparator Agents Against Pediatric Isolates of Enterobacterales, Pseudomonas aeruginosa, Acinetobacter baumannii-calcoaceticus species complex, and Stenotrophomonas maltophilia from the SENTRY Surveillance Program (2020-2022)	WoS	Conference abstract without full primary data
1081	Novel antibiotics: Are we still in the pre-post-antibiotic era?	WoS	Other (outside the scope of this review)
1082	1836. Frequency and Antimicrobial Susceptibility of Gram-negative Bacteria Isolated from Patients with Bloodstream Infections in United States (US) Medical Centers (2020-2021)	WoS	Conference abstract without full primary data
1083	Investigation of In Vitro Susceptibility to Ceftazidime-Avibactam and Ceftolozane-Tazobactam in Multidrug-Resistant Escherichia coli, Klebsiella pneumoniae and Pseudomonas aeruginosa	WoS	Focused on other β-lactamase inhibitor combinations
1084	Antibiotics and non-traditional antimicrobial agents for carbapenem-resistant Acinetobacter baumannii in Phase 1, 2, and 3 clinical trials	WoS	Other (outside the scope of this review)
1085	Treatment of Multi-Drug Resistant Gram-Negative Bacterial Pathogenic Infections	WoS	Other (outside the scope of this review)
1086	Unexpected Tricovalent Binding Mode of Boronic Acids within the Active Site of a Penicillin-Binding Protein	WoS	Other (outside the scope of this review)
1087	Treatment Approaches for Carbapenem-Resistant Acinetobacter baumannii Infections	WoS	Other (outside the scope of this review)
1088	Three-dimensional structure of AmpC β-lactamase from Escherichia coli bound to a transition-state analogue:	WoS	Structural or chemical-synthesis study

	Possible implications for the oxyanion hypothesis and for inhibitor design		
1089	Activity of Meropenem-Vaborbactam Against Enterobacteriaceae Isolates Carrying blaKPC Collected Worldwide	WoS	In vitro activity or surveillance data
1090	Multidrug-Resistant Gram-Negative Bacterial Infections in the Hospital Setting: Overview, Implications for Clinical Practice, and Emerging Treatment Options	WoS	General review or guidance (not boronate-focused)
1091	Role of newer and re-emerging older agents in the treatment of infections caused by carbapenem-resistant Enterobacteriaceae	WoS	Other (outside the scope of this review)
1092	In vitro activity of imipenem/relebactam, meropenem/vaborbactam and comparators against Enterobacterales from patients with intra-abdominal infections: Results of the study for Monitoring Antimicrobial Resistance Trends (SMART) in Taiwan, 2020	WoS	Focused on other β-lactamase inhibitor combinations
1093	Potentiation of cefotaxime against methicillin-resistant Staphylococcus aureus (MRSA) with the application of a novel adjuvant	WoS	Other (outside the scope of this review)
1094	Meropenem-Vaborbactam versus Ceftazidime-Avibactam for Treatment of Carbapenem-Resistant Enterobacteriaceae Infections	WoS	Other (outside the scope of this review)
1095	Site-protected fixation and immobilization of Escherichia coli cells displaying surface-anchored β-lactamase	WoS	Other (outside the scope of this review)
1096	Use of Boronic Acid Disk Tests To Detect Extended-Spectrum β-Lactamases in Clinical Isolates of KPC Carbapenemase-Possessing Enterobacteriaceae	WoS	Detection or diagnostic methodology
1097	New antibiotics for ventilator-associated pneumonia	WoS	Other (outside the scope of this review)
1098	Structures of ceftazidime and its transition-state analogue in complex with AmpC β-lactamase:: Implications for resistance mutations and inhibitor design	WoS	Structural or chemical-synthesis study
1099	1232. In Vitro Activity of Cefiderocol and Comparator Agents against Molecularly characterized Carbapenem-resistant Enterobacterales Clinical Isolates Causing Infection in United States Hospitals (2020)	WoS	Conference abstract without full primary data
1100	2549. Pharmacometric Analyses to Support Sulbactam-Durlobactam (SUL-DUR) Dosing Regimens in Patients with Altered Renal Function	WoS	Conference abstract without full primary data
1101	Functional analyses of AmpC β-lactamase through differential stability	WoS	PK/PD or pharmaceutical-technology study

1102	Characterization of β-lactamases responsible for resistance to extended-spectrum cephalosporins in Escherichia coli and Salmonella enterica strains from food-producing animals in the United Kingdom	WoS	Other (outside the scope of this review)
1103	Heteroresistance to cefepime-taniborbactam in metallo-β-lactamase-encoding Enterobacterales	WoS	Resistance-mechanism or variant study
1104	Therapeutic options for carbapenem-resistant Acinetobacter baumannii infections	WoS	Other (outside the scope of this review)
1105	LB2306. Population Pharmacokinetic (PPK), Pharmacokinetic/Pharmacodynamic attainment (PTA), and Clinical Pharmacokinetic/Pharmacodynamic (PK/PD) Analyses for Sulbactam-Durlobactam (SUL-DUR) to Support Dose Selection for the Treatment of Acinetobacter baumannii-calcoaceticus Complex (ABC) Infections	WoS	PK/PD or pharmaceutical-technology study
1106	Mechanisms of sulbactam-durlobactam resistance in Acinetobacter baumannii.	WoS	Other (outside the scope of this review)
1107	Meropenem plus vaborbactam - addition - EML	WoS	Other (outside the scope of this review)
1108	In vitro and in vivo activity of meropenem plus avibactam against MBL-producing carbapenem-resistant Klebsiella pneumoniae	WoS	In vitro activity or surveillance data
1109	2443. Searching for the Optimal Treatment Regimen for Metallo-β-Lactamase Producing Enterobacteriaceae: Aztreonam Plus Ceftazidime–Avibactam vs. Aztreonam Plus Meropenem–Vaborbactam	WoS	Conference abstract without full primary data
1110	Exploring Aztreonam in Combination with Ceftazidime-Avibactam or Meropenem-Vaborbactam as Potential Treatments for Metallo- and Serine-β-Lactamase-Producing Enterobacteriaceae	WoS	Combination-therapy study
1111	Activity of aztreonam-avibactam, cefiderocol, and cefepime-taniborbactam against a global collection of genetically characterized metallo-beta-lactamase-producing Enterobacterales.	WoS	Focused on cefiderocol or other novel agents
1112	Cefepime-zidebactam therapy for extensively drug-resistant Pseudomonas aeruginosa and Klebsiella pneumoniae infection as a bridge to liver transplantation	WoS	Focused on other β-lactamase inhibitor combinations
1113	Macrocyclic peptides: up-and-coming weapons to combat antimicrobial resistance	WoS	Other (outside the scope of this review)
1114	The Battle Is on: New Beta-Lactams for the Treatment of Multidrug-Resistant Gram-Negative Organisms	WoS	Other (outside the scope of this review)
1115	Targeting Nucleases via Transition	WoS	Other (outside the

	State Mimicry		scope of this review)
1116	1250. Novel Boronic Acid Transition State Analogs (BATSI) with in vitro inhibitory activity against class A, B and C β-lactamases	WoS	Conference abstract without full primary data
1117	Rapid evolution of pan- β-lactam resistance in Enterobacterales co-producing KPC and NDM: insights from global genomic analysis after the COVID-19 pandemic	WoS	Other (outside the scope of this review)
1118	Xeruborbactam	WoS	Other (outside the scope of this review)
1119	Carbapenem-Resistant Enterobacteriaceae	WoS	Other (outside the scope of this review)
1120	126. Frequency of Carbapenemases in United States Hospitals (2016-2020) and Activity of Meropenem-Vaborbactam and Comparator Agents Tested Against These Isolates	WoS	Conference abstract without full primary data
1121	1685. Antimicrobial Activity of Aztreonam-Avibactam Against Enterobacterales Causing Infection in United States Hospitals (2019-2021)	WoS	Conference abstract without full primary data
1122	Aztreonam-avibactam Activity against Enterobacterales from Asia Pacific and Latin American Medical Centres: Summary of 5 years of Surveillance prior to Clinical Use (2020-2024).	WoS	Focused on other β-lactamase inhibitor combinations
1123	P-1369. Impact of the E152K Mutation in Resistance to Taniborbactam and Evolutionary Advantages of NDM-9	WoS	Conference abstract without full primary data
1124	The Antibacterial Research and Development Pipeline Needs Urgent Solutions	WoS	General review or guidance (not boronate-focused)
1125	685. An In Vitro Investigation of WCK 5222 (Cefepime/Zidebactam) and Currently Available Combination Antibiotic Regimens Against Enterobacteriaceae That Co-express Serine-β-Lactamase (SBL) and Metallo-β-Lactamase (MBL) Enzymes	WoS	Conference abstract without full primary data
1126	Antibiotic dose-response curves can measure antibiotic activity against Mycobacterium abscessus and Mycobacterium peregrinum	WoS	Mycobacterium-focused
1127	P-1085. Characterization of Enterobacter cloacae and Citrobacter freundii Species Ccomplex Isolates with Decreased Susceptibility to Cephalosporins from United States Hospitals and Activity of Aztreonam-avibactam and Comparator Agents (2019-2023)	WoS	Conference abstract without full primary data
1128	New boronic acid compounds containing at least one heterocyclyl are beta lactamase inhibitors useful to treat infection caused by e.g. Staphylococcus aureus, Staphylococcus epidermidis, Streptococcus agalactiae and Streptococcus pneumoniae	WoS	Other (outside the scope of this review)

1129	New promising antibiotics for treatment of carbapenem resistant Enterobacteriaceae	WoS	Other (outside the scope of this review)
1130	New substituted boronic acid derivatives are beta lactamase inhibitors useful to treat or prevent bacterial infection caused by bacteria e.g. Pseudomonas acidovorans, Pseudomonas alcaligenes, Pseudomonas putida or Aeromonas hydrophilia	WoS	Other (outside the scope of this review)
1131	Mechanism Elucidation and Inhibitor Discovery against Serine and Metallo-β-Lactamases Involved in Bacterial Antibiotic Resistance	WoS	Other (outside the scope of this review)
1132	New heterocyclic boronic acid ester derivatives are beta-lactamase inhibitors useful to treat bacterial infection e.g. Mycobacterium tuberculosis, Haemophilus influenza, Pseudomonas aeruginosa, Helicobacter pylori and Campylobacter fetus	WoS	Mycobacterium-focused
1133	Novel Cyclic Boronate Cell Wall Inhibitors Targeting Carbapenem-Resistant Acinetobacter baumannii	WoS	Other (outside the scope of this review)
1134	1318. Pharmacokinetics and Safety of Cefepime-Taniborbactam (formerly Cefepime/VNRX-5133) in Subjects with Renal Impairment	WoS	Conference abstract without full primary data
1135	Antibiotic treatment of critically ill patients with sepsis: From FK/FD to novel drugs	WoS	Other (outside the scope of this review)
1136	Approaches to Testing Novel β-Lactam and β-Lactam Combination Agents in the Clinical Laboratory	WoS	Detection or diagnostic methodology
1137	What do We Know so Far about Ges Carbapenemases, and What Threat do They Pose?	WoS	Other (outside the scope of this review)
1138	Novel Antimicrobial Agents for Gram-Negative Pathogens	WoS	Other (outside the scope of this review)
1139	Outcomes and Predictors of Mortality in Patients With KPC-Kp Infections Treated With Meropenem Vaborbactam: An Observational Multicenter Study	WoS	Clinical-outcomes or pharmacoeconomic study
1140	Simple Disk-Based Method for Detection of Klebsiella pneumoniae Carbapenemase-Type β-Lactamase by Use of a Boronic Acid Compound	WoS	Detection or diagnostic methodology
1141	Case Report of Treatment of Polymicrobial Bacteremia Caused by Carbapenem-Resistant Klebsiella pneumoniae, and Bacillus Species in an Immunocompetent Patient With Meropenem-Vaborbactam	WoS	Case report
1142	Effect of Colistin, Fosfomycin and Meropenem/ Vaborbactam on Carbapenem-Resistant Enterobacterales in Egypt: A Cross-Sectional Study	WoS	Other (outside the scope of this review)

1143	P09 Activity of newer antibiotics against NDM- and OXA-48-producing organisms	WoS	In vitro activity or surveillance data
1144	Meropenem/Vaborbactam Approved in U.S. for Complicated Urinary Tract Infection	WoS	Other (outside the scope of this review)
1145	Ceftazidime/Avibactam and Meropenem/Vaborbactam for the Management of Enterobacterales Infections: A Narrative Review, Clinical Considerations, and Expert Opinion	WoS	General review or guidance (not boronate-focused)
1146	Epidemiology and in vitro activity of ceftazidime-avibactam, meropenem-vaborbactam, imipenem-relebactam, eravacycline, plazomicin, and comparators against Greek carbapenemase-producing Klebsiella pneumoniae isolates (vol 50, pg 467, 2022)	WoS	Focused on other β-lactamase inhibitor combinations
1147	In vitro Activity of “last chance” Antimicrobials against Klebsiella pneumoniae Complex	WoS	In vitro activity or surveillance data
1148	Validação do teste de inibição pelo ácido aminofenilborônico para triagem de Klebsiella pneumoniae carbapenemases (KPC)	WoS	Detection or diagnostic methodology
1149	Diverse Mechanisms of Resistance in Carbapenem-Resistant Enterobacteriaceae at a Health Care System in Silicon Valley, California	WoS	Other (outside the scope of this review)
1150	Novel Combination Product with Activity Against MRSA, MDR-Enterics and Pseudomonas	WoS	In vitro activity or surveillance data
1151	Effect of Meropenem-Vaborbactam vs. Piperacillin-Tazobactam on Microbial Eradication in UTI	WoS	Other (outside the scope of this review)
1152	In Vitro Activity of Old and New Antimicrobials against the Klebsiella pneumoniae Complex	WoS	In vitro activity or surveillance data
1153	P-1258. Unlocking Potentials: The Impact of Meropenem, Meropenem-vaborbactam, and Ceftazidime-avibactam in Combatting Carbapenem-Resistant Enterobacter cloacae in Epithelial Lining Fluid and Serum	WoS	Conference abstract without full primary data
1154	314. Ceftazidime-Avibactam Versus Meropenem-Vaborbactam for the Treatment of Carbapenem-Resistant Enterobacterales Infections	WoS	Conference abstract without full primary data
1155	Investigation of the Presence of Extended Spectrum Beta-Lactamase, KPC-Type Carbapenemase and Plasmid-Mediated AmpC Beta-Lactamase in E.coli and K.pneumoniae Strains Isolated from Blood Cultures	WoS	Other (outside the scope of this review)
1156	1378. Evaluation of the In vitro Activity of Meropenem-Vaborbactam Against Carbapenem-Resistant Enterobacteriaceae, Including Isolates Resistant to Ceftazidime–Avibactam	WoS	Conference abstract without full primary data

1157	Carbapenem-resistant Enterobacteriaceae: recent updates and treatment strategies	WoS	General review or guidance (not boronate-focused)
1158	Evolution of Klebsiella pneumoniae Sequence Type 512 during Ceftazidime/Avibactam, Meropenem/Vaborbactam, and Cefiderocol Treatment, Italy	WoS	Focused on cefiderocol or other novel agents
1159	In vitro activity of ceftazidime-avibactam and meropenem-vaborbactam against carbapenem-resistant Enterobacterales in Egyptian Hospitals: A challenge for clinical practice	WoS	In vitro activity or surveillance data
1160	High in vitro activity of Meropenem/RPX7009 against KPC-producing Enterobacteriaceae in China	WoS	In vitro activity or surveillance data
1161	Meropenem / Meropenem-vaborbactam	WoS	Other (outside the scope of this review)
1162	Review of In Vitro Efficacy of Ceftazidime-Avibactam, Meropenem-Vaborbactam and Imipenem-Relebactam in Carbapenemase Producing Klebsiella pneumoniae Isolates	WoS	Focused on other β-lactamase inhibitor combinations
1163	Evaluation of Boronic Acid Disk for Detection of Klebsiella pneumoniae Carbapenemase in Klebsiella pneumoniae	WoS	Detection or diagnostic methodology
1164	1114. Exploring bactericidal activity and emergence of resistance for carbapenem-resistant Enterobacterales (CRE) with and without Klebsiella pneumoniae carbapenemase (KPC) treated with optimized meropenem (MEM) and meropenem-vaborbactam (MEV) exposures	WoS	Conference abstract without full primary data
1165	The antibiotic arms race: current and emerging therapy for Klebsiella pneumoniae carbapenemase (KPC)-producing bacteria	WoS	Other (outside the scope of this review)
1166	Determination of Escherichia coli and Citrobacter koseri Isolates with OXA-48 Carbapenemase in Istanbul	WoS	Other (outside the scope of this review)
1167	2183. Porin Mutations Potentiate the in Vitro Synergy of Meropenem-Vaborbactam (MVB), but not Ceftazidime-Avibactam (CZA) in Combination with Other Antibiotics against KPC-producing Klebsiella Pneumoniae (KPC-Kp)	WoS	Conference abstract without full primary data
1168	Antibiotic susceptibility to new antibiotics and genetic characterisation of carbapenemase-producing Enterobacterales: Low activity of cefiderocol against NDM-producing isolates	WoS	Focused on cefiderocol or other novel agents
1169	Spontaneous bacterial peritonitis due to carbapenemase-producing	WoS	Other (outside the scope of this review)

	Enterobacteriaceae: Etiology and antibiotic treatment		
1170	Characterization of VIM-1-, NDM-1-and OXA-48-producing Citrobacter freundii in France	WoS	Other (outside the scope of this review)
1171	Evaluation of the Therapeutic Outcomes of Antibiotic Regimen Against Carbapenemase-Producing Klebsiella pneumoniae: A Systematic Review and Meta-Analysis	WoS	Clinical-outcomes or pharmacoeconomic study
1172	Updates in Management of Complicated Urinary Tract Infections: A Focus on Multidrug-Resistant Organisms	WoS	General review or guidance (not boronate-focused)
1173	1443. Activity of Ceftazidime-Avibactam against Carbapemenase-negative Carbapenem-resistant Enterobacterales (CRE) Isolates from US Hospitals	WoS	Conference abstract without full primary data
1174	Emerging Challenges and Innovations in the Management of Carbapenem-resistant Enterobacteriaceae Infections in Hospital Settings"	WoS	Other (outside the scope of this review)
1175	Challenges in beta-Lactamase Mediated Resistance	WoS	Other (outside the scope of this review)
1176	Primer aislamiento de una cepa de Klebsiella pneumoniae productora de carbapenemasa tipo KPC-1 en el Hospital de San Carlos durante el 2011	WoS	Other (outside the scope of this review)
1177	Recent advances in the pharmacological management of infections due to multidrug-resistant Gram-negative bacteria	WoS	Other (outside the scope of this review)
1178	Current and future perspectives in the treatment of multidrug-resistant Gram-negative infections	WoS	General review or guidance (not boronate-focused)
1179	High minimum inhibitory concentrations among derepressed AmpC-beta-lactamase-producing Enterobacter cloacae complex isolates for ceftolozane with tazobactam	WoS	Focused on other β-lactamase inhibitor combinations
1180	Targeting Enzymes Involved in Antimicrobial Resistance (Amr) in Gram-Negative Bacteria	WoS	Other (outside the scope of this review)
1181	1601. Combination Therapy versus Monotherapy for Carbapenem-resistant Organisms: Is More Really Better?	WoS	Conference abstract without full primary data
1182	1110. Why has Utilization of Novel Gram-negative Antibiotics been Sluggish in U.S. Hospitals? A Retrospective Cohort Study to Inform Future Antibiotic Development and Market Entry Rewards	WoS	Conference abstract without full primary data
1183	Defining the architecture of KPC-2 Carbapenemase: identifying allosteric networks to fight antibiotics resistance	WoS	Other (outside the scope of this review)
1184	1592. Antimicrobial Activity of Ceftazidime-Avibactam and Comparator Agents Against	WoS	Conference abstract without full primary data

	Enterobacterales and Pseudomonas aeruginosa With Overexpression of AmpC β-Lactamase From Phase 3 Clinical Trials		
1185	Relación clonal y detección del gen blaKPC en cepas de Klebsiella pneumoniae resistentes a carbapenémicos, en un hospital de Venezuela	WoS	Other (outside the scope of this review)
1186	Influence of substrates and inhibitors on the structure of Klebsiella pneumoniae carbapenemase-2	WoS	Structural or chemical-synthesis study
1187	115. Influence of Antimicrobial Stewardship and Molecular Rapid Diagnostic Test on Antimicrobial Prescribing for Extended-spectrum Beta-lactamase and Carbapenemase-producing Bacteria in Bloodstream Infections	WoS	Conference abstract without full primary data
1188	Phenylboronic Acids Probing Molecular Recognition against Class A and Class C β-lactamases	WoS	Other (outside the scope of this review)
1189	682. In Vivo Pharmacodynamics of VNRX-7145 in the Neutropenic Murine Thigh Infection Model When Administered in Combination with Humanized Exposures of Twice Daily Ceftibuten (CTB) Against Serine β-Lactamase-Producing Enterobacteriaceae (SBL-EB)	WoS	Conference abstract without full primary data
1190	1285. Identification of KPC Omega Loop Variant R163S Conferring Ceftazidime-Avibactam Resistance	WoS	Conference abstract without full primary data
1191	An effective two-stage key frame extraction method for speech-visual emotion recognition	WoS	Other (outside the scope of this review)
1192	Colonization of High-Risk Children with Carbapenemase-Producing Enterobacteriaceae in Greece	WoS	Other (outside the scope of this review)
1193	KPC-50 Confers Resistance to Ceftazidime-Avibactam Associated with Reduced Carbapenemase Activity	WoS	Other (outside the scope of this review)
1194	2756. Activity of Aztreonam-avibactam and Ceftazidime-avibactam against United States Isolates Producing β-lactamases (2020–2021)	WoS	Conference abstract without full primary data
1195	2798. Difficult to treat GES and KPC harboring Pseudomonas aeruginosa: Evaluation of in vivo efficacy of ß-lactam/ß-lactamase inhibitor humanized exposure vs. in vitro efficacy	WoS	Conference abstract without full primary data
1196	2174. Cefiderocol Activity Against Multidrug-resistant and Molecularly Characterized Pseudomonas aeruginosa and Acinetobacter baumannii-calcoaceticus complex Clinical Isolates Causing Infection in United States Hospitals (2020–2022)	WoS	Conference abstract without full primary data
1197	124. Activity of Novel b-Lactam/b-	WoS	Conference abstract

	Lactamase Inhibitor (BL/BLI) Combinations Against AmpC-Producing Species Collected in United States Hospitals		without full primary data
1198	643. Cefiderocol In vitro Activity Against Molecularly Characterized Pseudomonas aeruginosa and Acinetobacter baumannii-calcoaceticus complex Clinical Isolates Causing Infection in United States Hospitals (2020-2021)	WoS	Conference abstract without full primary data
1199	Analysis of the carbapenemase-producing mechanism of the enterobacteriaceae with decreased susceptibility to carbapenems and construction of the recombinant plasmid with blakpc	WoS	In vitro activity or surveillance data
1200	1218. Activity of Meropenem-Vaborbactam and Comparator Agents Against Carbapenemase-Negative, Carbapenem-Resistant Enterobacterales from US Hospitals	WoS	Conference abstract without full primary data
1201	202. Comparative Activity of Ceftazidime-Avibactam, Imipenem-Relebactam and Meropenem-Vaborbactam Tested Against Carbapenem-Nonsusceptible Enterobacterales that Are Carbapenemase-Negative	WoS	Conference abstract without full primary data
1202	Antibiotic resistance among hypermutator carbapenem resistant Klebsiella pneumoniae	WoS	Other (outside the scope of this review)
1203	Use of site-specific pharmacokinetics to optimize antibiotic combinations and prevent the emergence of resistance against CRE	WoS	PK/PD or pharmaceutical-technology study
1204	Characterization and suppression of resistance to new CRE agents	WoS	Other (outside the scope of this review)
1205	Understanding B-Lactam Resistance in Acinetobacter baumannii	WoS	Other (outside the scope of this review)
1206	New definitions of extended-spectrum ß-lactamase conferring worldwide emerging antibiotic resistance	WoS	Other (outside the scope of this review)
1207	Klebsiella pneumoniae carbapenemase variant 44 acquires ceftazidime-avibactam resistance by altering the conformation of active-site loops	WoS	Other (outside the scope of this review)
1208	E. coli y K. pneumoniae productores de BLEE, blaKPC, blaVIM y blaNDM en un hospital de referencia, Trujillo-Perú	WoS	Other (outside the scope of this review)
1209	Effects of empagliflozin and metformin on biofilm formation and pathogenicity factors of urinary Escherichia coli isolates	WoS	Other (outside the scope of this review)
1210	Clonal Spread of Extended-Spectrum Cephalosporin-Resistant Enterobacteriaceae Between Companion Animals and Humans in South Korea	WoS	Other (outside the scope of this review)
1211	Efficacy and safety of	WoS	Other (outside the

	doravirine/lamivudine/tenofovir as initial treatment for people living with HIV in China		scope of this review)
1212	The role of new antimicrobials for Gram-negative infections in daily clinical practice	WoS	Other (outside the scope of this review)
